# Redox‐Active Verdazyl Radical Polymers via Postpolymerization Modification of Active Ester Polymers

**DOI:** 10.1002/cplu.70269

**Published:** 2026-10-27

**Authors:** Theo A. Ellingsen, James D. Randall, Bhagya Dharmasiri, Wesley J. Olivier, Luke C. Henderson, Melissa K. Stanfield, Stuart C. Thickett, Rebecca O. Fuller

**Affiliations:** ^1^ School of Natural Sciences ‐ Chemistry University of Tasmania Hobart Tasmania Australia; ^2^ Institute for Frontier Materials Deakin University Waurn Ponds Victoria Australia

**Keywords:** organic radical polymers, polymers, radicals, stable organic radicals, verdazyls

## Abstract

Organic radical polymers (ORPs) are constructed with 1,5‐dimethyl‐6‐oxoverdazyl radicals for assessment as redox‐active materials. The ORPs were synthesized through the postpolymerization modification (PPM) of poly(pentafluorophenyl methacrylate) (**P1**) and poly(pentafluorophenyl acrylate) (**P2**) with either 1,5‐dimethyl‐3‐(*p‐*aniline)‐6‐oxoverdazyl (**1**), 1,5‐dimethyl‐3‐(4‐methylamine‐(1,1ʹ‐biphenyl))‐6‐oxoverdazyl (**2**) or 1,5‐dimethyl‐3‐(4‐methylamine‐(1,1ʹ‐biphenyl))‐6‐oxoverdazyl (3). To overcome the limited stability of **1**, radicals containing bulky C_3_ substituents and alkyl spacers to the heteroatom of the 4‐methylamine or 4‐hydroxymethyl moiety were synthesized from high yielding (78%–98%) Suzuki–Miyaura cross‐couplings. The large cell potential (E°_cell_(**3**) = 1.67 V) and radical stability ensure these are well suited to fundamental electrochemical proof of concept studies when incorporated into a polymer. Variable radical attachment (0%–75%) was observed in PPM reactions. The ester aminolysis of **P2** yielded the highest radical containing polymer (**P2‐2**; 75%). As a proof‐of‐concept electrochemical demonstration, **P2‐2** and **P2‐3** exhibited a reversible redox couple at 3.43 V (vs. Li/Li^+^), successfully validating the redox activity of the verdazyl‐functionalized polymers in a coin cell.

## Graphical abstract

Organic radical polymers were synthesized via postpolymerization modification (PPM) of poly(pentafluorophenyl (meth)acrylate) with diverse 6‐oxoverdazyl stable radicals. This work servs as an electrochemical proof‐of‐concept, exhibiting a chemically reversible redox couple at 3.43 V versus Li/Li^+^, validating the electrochemical accessibility of the redox‐active polymers in a coin cell.

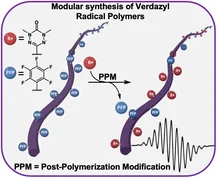

## 1 Introduction

Organic radical polymers (ORPs) have garnered increasing research attention across an array of evolving applications owing to their unique electrochemical and physical properties. These macromolecules consist of a traditional polymeric backbone functionalized with stable organic radical pendants. Careful construction of these architectures ensures the adaptability of chemicophysical behavior. Mechanical and thermal properties of ORPs can be largely determined by the main carbon chain, redox activity by the open shell species, and charge transport properties by both the polymer and radical species. This offers a myriad of design choices for the next generation of electrochemically active polymers suited to greener energy storage [1, 2, 3, 4, 5], catalysis [6, 7], and spintronic applications [8, 9, 10, 11]. Although radical chemistry is an essential part in the development of these systems, it is often overlooked, with most contemporary examples based on nitroxide radicals. The field is dominated by ORPs constructed with 2,2,6,6‐tetramethyl‐1‐piperidinyloxyl (TEMPO) pendants on a poly(methacrylate) backbone (PTMA) [2, 4, 12, 13] as well as other nitroxide radical and polymer pairings [14, 15]. The extensive use of this family is in part attributed to the ease of access and synthesis from commercial materials, including monomers. However, deficiencies regarding the long‐term stability of nitroxide ORPs warrant exploration of alternative systems to mitigate this [16].

Greener energy storage using ORPs has undergone a significant diversification of macromolecule motifs in the last 20 years [4, 11, 12, 17, 18, 19, 20, 21, 22, 23, 24, 25, 26, 27, 28, 29, 30, 31]. Although PTMA represents the most studied system, new synthetic methodologies have been driving the production of many innovative architectures containing verdazyl [11, 17, 18, 19, 20], Blatter [21, 22, 23], galvinoxyl [4, 24, 25, 26], and alternative nitroxide ORPs with a variety of polymer backbones [12, 27, 28, 29, 30, 31]. Verdazyl radicals were first synthesized in the early 1960s [32] and subsequently used in ORPs in the latter half of that decade [33, 34]. These macromolecules remained curiosities for nearly 50 years, with renewed interest involving the preparation of the first 6‐oxoverdazyl ORP [17]. Since then, a handful of further verdazyl‐ORPs have been synthesized (Figure 1) using: (i) free radical polymerization of a tetrazine‐functionalized monomer, then postoxidation [17]; (ii) direct ring‐opening metathesis polymerization of a monomer containing a verdazyl radical [18, 20, 35]; and (iii) postpolymerization modification (PPM) using copper‐assisted azide–alkyne cycloaddition reaction (CuAAC) [11, 19]. Verdazyl‐ORPs are predicted to exhibit superior charge transport capabilities relative to their nitroxide counterparts due to extensive radical delocalization [36]. While their capability to exhibit a two‐electron transfer process in aqueous flow batteries is established [37], the redox behavior of these ORPs remains untested in a coin‐cell configuration. ORPs such as PTMA are redox‐active materials [38], undergoing oxidation at a relatively high potential (3.5–4.5 V vs. Li/Li^+^). To date, none of the verdazyl‐ORPs have been assessed directly for their electrochemical properties in a coin cell architecture.

**FIGURE 1:**
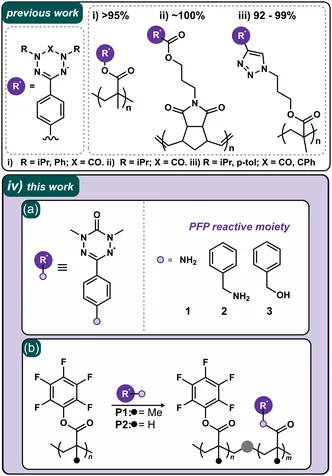
Examples of methods used to synthesize verdazyl ORPs including (i) postoxidation of a polymeric verdazyl precursor [17], (ii) direct polymerization of a radical containing monomer [18, 20], (iii) PPM via copper‐assisted azide alkyne cycloaddition [19], and (iv) the strategy developed in this work, where amine‐ and hydroxy‐functionalized verdazyls (a) are incorporated into activated PFP ester polymers (b) through PPM.

To address this, we report a proof‐of‐concept demonstration evaluating the electrochemical behavior of 6‐oxoverdazyl‐ORPs. PPM of activated pentafluorophenyl (PFP) ester polymers has been used as a new strategy to achieve the scalable synthesis of 6‍‐‍oxoverdazyl‐ORPs. Although PPM of activated ester polymers has found widespread adoption to synthesize highly functionalized macromolecules [39, 40, 41, 42], only one ORP example is reported [12]. Poly(pentafluorophenyl methacrylate) (**P1**) and poly(pentafluorophenyl acrylate) (**P2**) polymers represent the most commonly used PPM scaffolds owing to the reactivity of PFP ester with nucleophiles such as amines or alcohols via ester aminolysis or transesterification, respectively [41, 43, 44, 45, 46]. This approach afforded a route to nitroxide‐ORPs with high (96%) radical incorporation, surpassing the more commonly used postoxidation techniques which typically yield radical concentrations of 65%–81% [12]. Building upon previous work [47, 48, 49], we have synthesized three 6‐oxoverdazyl radicals bearing amine or hydroxyl groups suitable for PPM with **P1** and **P2**. These functional groups were installed onto the tetrazinanone precursors via catalytic hydrogenation or a Suzuki–Miyaura cross‐coupling prior to generation of the radical through oxidation. The PPM reaction conditions were optimized for each radical pairing with **P1** and **P2**, and the suitable 6‐oxoverdazyl‐ORPs were then evaluated to validate the fundamental redox activity of verdazyl‐functionalized polymers. Finally, we discuss the current limitations of these architectures, detailing the active material utilizations and performance thresholds required to transition these proof‐of‐concept materials toward viable applications.

## 2 Results and Discussion

PPM of activated PFP ester polymers was used to generate a small library of verdazyl‐ORPs (Figure 1iv). To evaluate the efficacy of both ester aminolysis and transesterification, three radical precursors (Figure 2) were synthesized: 1,5‐dimethyl‐3‐(*p‐*aniline)‐6‐oxoverdazyl (**1**)**,** 1,5‐dimethyl‐3‐(4‐methylamine‐(1,1′‐biphenyl))‐6‐oxoverdazyl (**2**), and 1,5‐dimethyl‐3‐(4‐hydroxymethyl‐(1,1′‐biphenyl))‐6‐oxoverdazyl (**3**). Reaction conditions for **P1** and **P2** were optimized on a small scale (~15 mg of PFP ester polymer). The Evans method was used to quantify radical incorporation (Supporting Information, Experimental) [50]. Larger‐scale syntheses yielded sufficient material for the characterization of the physical and electrochemical properties of the new ORPs.

**FIGURE 2:**
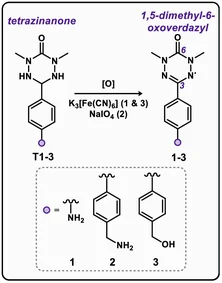
Compounds investigated in this work. 6‐Oxoverdazyl radicals **1–3** were synthesized by the oxidation of tetrazinanones **T1‐3**. Functional groups amenable to active ester PPM with **P1** and **P2** were incorporated at the C_3_ position.

### 2.1 Synthesis of 6‐Oxoverdazyl Radicals

Radicals **2** and **3** represent previously unreported 6‐oxoverdazyl derivatives, whereas radical **1** is a known compound described in the literature [51], prepared via an alternative pathway. All three radicals were prepared using a synthetic methodology that avoids the use of methylhydrazine and phosgene [47, 48] to help improve the safety and accessibility of these compounds. The synthesis of **1–** **3** (Figure 2) involves the oxidation of precursor tetrazinanone (**T1**‐**T3**). 6‐(4‐aminophenyl)‐2,4‐dimethyl‐1,2,4,5‐tetrazinan‐3‐one (**T1**) was synthesized in an overall yield of 59% over three steps (Scheme S1). The final step is a catalytic hydrogenation of 2,4‐dimethyl‐6‐(4‐nitrophenyl)‐1,2,4,5‐tetrazinan‐3‐one [52, 53], whereby the reduced nitro group afforded the aniline‐functionalizedd precursor **T1** in high yield (0.45 g, 94%). Oxidation of **T1** with K_3_[Fe(CN)_6_] [51] gave 1,5‐dimethyl‐3‐(*p‐*aniline)‐6‐oxoverdazyl **1** in 53% yield (Figure S27). During optimization, thin layer chromatography (TLC) revealed the formation of several decomposition compounds, necessitating very short reaction times (~10 min). Upon isolation, **1** decomposed rapidly under both ambient and inert atmospheres at −18 °C. Electron Paramagnetic Resonance (EPR) spectroscopy was employed to investigate the stability of **1**, showing that the peak amplitude decreased by 62% after 24 h, with only trace radical signal detected after 29 days (Figure S28). Aniline degradation is well known, with **1** previously reported to decompose at 75 °C [51]. As a reduction in the aniline groups reactivity is achievable through delocalization of the phenyl electrons, attachment to a polymer is anticipated to prevent further decomposition. EPR spectrum for **1** exhibited the characteristic 11 + 2 line group (Figure S27), with the three hyperfine coupling constants (*a*(N_2,4_) = 6.5 G, *a*(N_1,5_) = 5.2 G, and *a*(H) = 4.9 G) and g‍‐‍factor (*g* = 2.0030) in good agreement with the previously reported values [51]. In contrast to typical 6‐oxoverdazyls, which exhibit two fully reversible one‐electron redox couples (oxidation of the 7π verdazyl to the 6π cation and reduction 8π anion), the cyclic voltammogram of **1** did not contain two full reversible redox processes. Instead, a reversible oxidation electron process alongside an additional oxidation process likely associated with the amine functional group (Figure S42) was observed. The reduction of **1** at slower scan rates appeared to be irreversible, causing the stable oxidation process to decrease in current as the radical decomposed (Figure S43). The cell potential (1.58 V) of **1** is similar to that of other 6‐oxoverdazyls (c.f. ~1.55 V) [54]. Freshly prepared **1** was used in PPM reactions (see below).

Strategies to improve verdazyl stability include increasing steric bulk at the N_1,5_ positions (Figure 2) or ensuring that the C_3_ substituent is not susceptible to degradation [55]. The replacement of 1,5‐dimethyl groups with isopropyl or phenyl analogs is well established as a method to alleviate stability concerns [55, 56]. Although this represents the simplest approach, steric bulk at these positions (Figure 2) may hinder PPM and fails to address the inherent instability of the *p‐*aniline moiety. We note that a smaller N_1,5_ substituent, an ethyl c.f. methyl group in 1,5‐diethyl‐3‐(*p‐*aniline)‐6‐oxoverdazyl only exhibits marginal improvements in stability (~48% remaining overnight) relative to **1** (~38% remaining overnight) [57]. The insertion of an alkyl spacer serves to decouple the amine from the aromatic system and prevent the oxidative degradation pathways typical of anilines [58] and was targeted to improve stability and minimize bulk at the N_1,5_ positions.

Radical **2** (Figure 2) contains an alkyl spacer group between the *p*‐amino (─CH_2_NH_2_) group of the functionalized phenyl moiety attached to the verdazyl C_3_ atom. Radical **3** was also prepared and features a hydroxymethyl group (─CH_2_OH), providing an alternative nucleophile handle for transesterification‐based PPM (c.f. ester aminolysis with the ─NH_2_ group). The addition of the methylene group in **2** should also increase the nucleophilicity c.f. **1**, overcoming the limited reactivity of anilines toward **P2** (14%) compared to alkyl amines (e.g., 99% for hexylamine) [43]. Precursors (Figure 2) 6‐(4′‐(aminomethyl)‐[1,1′‐biphenyl]‐‍4‍‐‍yl)‐2,4‐dimethyl‐1,2,4,5‐tetrazinan‐3‐one (**T2**) and 6‐(4′‐(hydroxymethyl)‐[1,1′‐biphenyl]‐4‐yl)‐2,4‐dimethyl‐1,2,4,5‐tetrazinan‐3‐one (**T3**) were synthesized via Suzuki–‍Miyaura cross‐coupling of 6‐(4‐bromophenyl)‐2,4‐dimethyl‐1,2,4,5‐tetrazinan‐3‐one [49, 59], with (4‐(aminomethyl)phenyl)boronic acid and (4‐(hydroxymethyl)phenyl)boronic acid, respectively (Scheme S2). We employed a new, high‐yielding route to the (4‐(aminomethyl)phenyl)boronic acid precursor [60], achieving 64% yield over two steps, a significant improvement over established protocols (34% over two steps) [61]. The Suzuki–Miyaura coupling proceeded efficiently, affording the verdazyl precursors, **T2** and **T3,** in high yields (78% and 94%), with four‐step yields of 37% (0.74 g) and 45% (1.31 g) for **T2** and **T3,** respectively.

1,5‐Dimethyl‐3‐(4‐methylamine‐(1,1′‐biphenyl))‐6‐oxoverdazyl (**2**) was synthesized via the oxidation of **T2** with NaIO_4_. Alternative oxidants including K_3_[Fe(CN)_6_], *p*‐benzoquinone, PbO_2_, and FeCl_3_ were investigated; however, these conditions either failed to afford **2** or led to substantial side‐product formation as indicated by TLC. While the use of NaIO_4_ has been associated with radical decomposition in some verdazyl radicals [62], employing a 1:1 mixture of 0.05 M acetic acid and MeCN at 50 °C proved successful with **2** isolated as a bright‐red solid (81%, 0.084 g). Solution stability studies indicated that in solution within a sealed capillary, **2** remained stable for 3 days (<5% decomposition). Decomposition then occurred rapidly thereafter, reaching 87% loss of radical after 1 week (Figure S29). As a solid in ambient conditions, **2** exhibited similar short‐term stability (~5% decomposition over 3 days), with 88% decomposition observed over 30 days (Figure S30). For this reason, fresh samples of **2** were always prepared immediately prior to subsequent PPM reactions. The EPR spectrum of **2** exhibited a typical 11 + 2‐line group (Figure S31). The three hyperfine coupling constants used to fit the experimental data (*a*(N_2,4_) = 6.4 G (17.9 MHz); *a*(N_1,5_) = 5.3 G (14.9 MHz); and *a*(H) = 5.3 G (14.9 MHz)) are typical for 6‐oxoverdazyl radicals [51]. The cyclic voltammogram of **2** (Figure S44) showed a reversible reduction process (at scan‐rates 50–1000 mV/s), with the oxidation wave found to be irreversible (Figures S45 and S46). The reduction occurs at a similar potential, −1.2 V versus Fc/Fc^+^ c.f ~ −1.3 V, to other 6‐oxo‐verdazyls, and the cell potential, 1.54 V, is also typical for 6‐oxoverdazyl radicals [54].

1,5‐Dimethyl‐3‐(4‐hydroxymethyl‐(1,1′‐biphenyl))‐6‐oxoverdazyl (**3**) was synthesized as a red solid from the oxidation of **T3** with K_3_[Fe(CN)_6_] in aqueous methanol containing sodium carbonate. Radical **3** exhibited excellent stability; solution studies revealed limited decomposition (8%) over 21 days (Figure S32). The solid is bench‐stable after storage in ambient conditions, exposed to light for >1 year (Figure S33).

The EPR spectrum of **3** exhibited the characteristic 11 + 2 line pattern observed for 6‐oxoverdazyl radicals (Figure 3A) [47, 64], with hyperfine coupling constants of *a*(N_2,4_) = 6.4 G, *a*(N_1,5_) = 5.3 G, and *a*(H) = 5.0 G. In contrast to **1** and **2**, both the one‐electron oxidation and reduction processes were observed in **3** using cyclic voltammetry (CV) measurements in a standard potential window (Figure 3B) [54]. Electrochemical analysis confirmed that the reduction and oxidation processes are reversible at various scan rates between 50 and 1000 mV/s. We observe that **3** has an E^0^_cell_ of 1.67 V, representing, to the best of our knowledge, the largest potential exhibited by 6‐oxoverdazyl radicals in organic electrolytes with two reversible redox processes (Figure ‍3B) [54, 65, 66]. The combination of excellent stability in the solid state and in solution under ambient conditions, along with its high cell potential, makes this radical an attractive candidate for the development of verdazyl‐ORPs.

**FIGURE 3:**
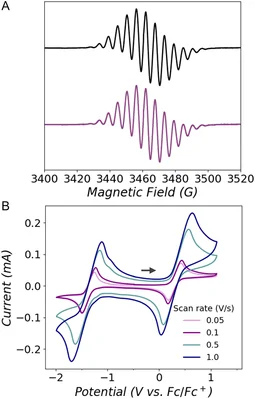
(A) The first derivative X‐band (CW) EPR spectrum (*ν *= 9.69 GHz) of **3** in degassed toluene at 298 K (upper) together with simulation (lower) using EasySpin [63] garlic package in MATLAB. A field modulation of 2.1 G and power of 40 mW (12 dB attenuation) was used. (B) Cyclic voltammogram of **3** (11.5 mM) at varying scan rates in dichloromethane using nBu_4_NPF_6_ (10 mM) as supporting electrolyte. Solutions are plotted (IUPAC) versus ferrocene/ferrocenium (*E*_1/2_ = 0 V) with a CH Instruments glassy carbon working electrode, Pt wire counter electrode and a leakless Ag/AgCl reference electrode.

### 2.2 Optimization of Verdazyl‐ORP Synthesis Using PPM

Poly(pentafluorophenyl methacrylate) (**P1**) and poly(pentafluorophenyl acrylate) (**P2**) were selected as polymer scaffolds because they combine the precise control afforded by reversible addition−fragmentation chain transfer (RAFT) polymerization with the versatility of activated ester chemistry. This approach allows for the controlled tuning of the polymer backbone while ensuring facile access to ORP motifs via ester aminolysis or transesterification. **P1** and **P2** were synthesized using the RAFT polymerization of pentafluorophenyl methacrylate and pentafluorophenyl acrylate, respectively [41, 43]. Gel permeation chromatography (GPC) was employed to determine the molecular weight and dispersity of the synthesized polymers (Figure S37), confirming the synthesis of well‐defined polymers with relatively low dispersity (**P1:** *Đ* = 1.38; **P2:** *Đ* = 1.22) and comparable number‐average molecular weights (**P1:** *M*_n_ = 11.0 kDa; **P2:** *M*_n_ = 8.6 kDa). Notably, while literature protocols typically employ lower initiator loadings (e.g., [CTA]:[Initiator] = 8:1) [44], in our hands, controlled polymerization was only achievable using a higher initiator concentration ([CTA]:[Initiator] = 2:1) [41].

The quantification of spin concentration in the synthesized ORPs was performed using the Evans NMR method (Supporting Information, Experimental) [50, 57, 67, 68]. Complementary quantitative ^19^F‐NMR spectroscopy was employed to determine the extent of PFP ester cleavage (Supporting Information, Experimental). Comparison of the total PFP ester consumption with the radical attachment efficiency (determined via the Evans method) allowed for the quantification of both successful functionalisation and side reactions, such as ester hydrolysis.

#### 2.2.1 Optimization of Ester Aminolysis PPM With Radical Nucleophiles 1 and 2

Given the diversity of conditions available for PFP ester modification, reaction parameters were independently optimized for each polymer–radical pair. In line with the broader field, key variables include solvent selection, Lewis base catalyst, and nucleophile molar excess [12]. Table S1 summarizes preliminary optimization studies for the coupling of radical **1** with **P1**. Given the established thermal instability of **1**, reaction parameters were restricted to a maximum of 50 °C and 5 h. **1** exhibited sufficient nucleophilicity to react with **P1** in the absence of a Lewis base catalyst, as evidenced by the observed cleavage of the PFP ester by ^19^F‐NMR (Table S1). Quantification via the Evans method was omitted for these screening reactions as the polymer isolation steps resulted in significant decomposition. Optimization focused on solvent selection and radical stoichiometry. Solvent choice proved critical, with 1,2‐dimethoxyethane (DME) yielding the highest PFP ester cleavage (53%, Table S1; entry 4). Conversely, increasing the loading of **1** from 1.5 to 2.0 equivalents led to a slight reduction in conversion. An inability to increase the scale of oxidation used to prepare **1** and the radicals limited stability rendered further optimization or large‐scale ORP preparation unfeasible. Consequently, our focus shifted to the more robust radical **2**.

Verdazyl‐ORP **P1‐2** was synthesized using an ester aminolysis of poly(pentafluorophenyl methacrylate), **P1**, with methylamino radical **2** (Table 1). Considerable optimization was required. Initial attempts did not utilize a Lewis base, relying on the intrinsic nucleophilicity of the amine functional group of the radical; however, reactivity was poor. Using DME yielded only 7% functionalisation (Table 1; entry 1). Alternative solvents did not improve this, with no detectable radical attachment and varying degrees of PFP cleavage observed (Table 1; entries 2–4). Attempts to use pyridine as both solvent and catalyst also proved unsuccessful with no radical incorporation detected, despite significant PFP ester cleavage (Table 1; entry 5). Catalyst (Lewis base) screening revealed further challenges. The addition of triethylamine (TEA) yielded ORPs that were either entirely insoluble or exhibited negligible radical incorporation (Table 1; entries 6–‍‍‍‍‍‍‍‍8). Use of 4‐dimethylaminopyridine (DMAP) as a catalyst was found to improve ester aminolysis. Lower equivalents of DMAP (0.2 equivalents) resulted in modest attachment (22%), with PFP cleavage corresponding solely to the radical attachment (Table 1; entry 9). When these conditions were extended overnight at 80 °C, there was no observable attachment of radical **2** (Table 1; entry 10). Radical disproportionation in DMF at elevated temperatures and longer reaction times are the likely cause. To minimize this, a 2.0 molar DMAP excess was used in the modest (50 ºC, 5 hrs) standard PPM conditions, yielding significantly higher radical attachment of 53% (Table 1; entry 11). For these conditions, near‐quantitative PFP ester cleavage was observed via ^19^F‐NMR (99%). This discrepancy could suggest that significant hydrolysis occurred or radical decomposition.

**TABLE 1:** Postpolymerization conditions for the optimization of the synthesis of **P1‐2**. Reactions were performed at 50 °C for 5 h unless otherwise stated.

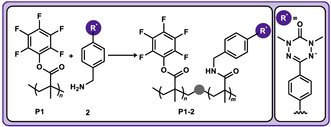					
Entry	Base	Solvent	**[Polymer]/** **[radical]/** **[base]**	PFP reacted, %	Evans method, %
1	—	DME	1.0/1.5/0.0	12	7
2	—	DMF	1.0/1.5/0.0	1	0
3	—	THF	1.0/1.5/0.0	1	0
4	—	MeCN	1.0/1.5/0.0	1	0
5	—	Pyridine	1.0/1.5/0.0	52	0
6	TEA	DME	1.0/1.5/2.0	28	0
7	TEA	DME	1.0/2.0/2.0	33	11
8	TEA	DMF	1.0/2.0/2.0^a^	37	—c
9	DMAP	DMF	1.0/1.2/0.2	22	22
10	DMAP	DMF	1.0/1.2/0.2^b^	79	0
11	DMAP	DMF	1.0/1.5/2.0	99	53

a Reaction performed at 80 °C.

b 18 h reaction.

c Product was insoluble and radical concentration could not be measured.

Optimization reactions for the synthesis of **P2‐2**, using ester aminolysis of poly(pentafluorophenyl acrylate) **P2** with methylamino radical **2**, are presented in Table 2. Initial attempts with radical **2** showed moderate reactivity toward **P2** in the absence of an additional Lewis base catalyst. The increased reactivity of the acrylate **P2** c.f. methacrylate **P1** toward PPM is well known and not unexpected [43]. When the reaction was conducted with 1.5 equivalents of **2** in DME, a polymer with 15% radical functionalisation (Table 2; entry 1) was observed; this increased to 27% with 2.0 molar equivalents of radical (Table 2; entry 2). The base catalyst was optimized in DME. The addition of triethylamine (TEA) (2 equivalents) was found to lower the **P2‐2** radical concentration (17%, Table 2; entry 3). Longer reaction times (72 h) revealed observable (by ^19^F‐NMR) side reactions (Table 2; entry 4). Decreasing TEA loading (0.2 equivalents) improved radical attachment (30%, Table 2; entry 5), and the alternative DMAP base catalyst (0.2 equivalents) provided further improvement (44%, Table 2; entry 6). Increasing the reaction temperature to 80 °C (c.f. 50 °C), and radical molar equivalents proved unsuccessful (Table 2; entry 7). Solvent selection proved to be a critical parameter as the substitution of DME with DMF led to a substantial increase in the ester aminolysis, achieving 79% radical incorporation (Table 2; entry 8).

**TABLE 2:** Postpolymerization conditions for the optimization of the synthesis of **P2‐2**. Reactions were performed at 50 °C unless otherwise stated.

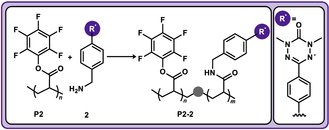					
Entry	Base	Solvent	**[Polymer]/** **[radical]/** **[base]**	Time, h	PFP reacted, %	Evans method, %
1	—	DME	1.0/1.5/0.0	5	38	15
2	—	DME	1.0/2.0/0.0	5	33	27
3	TEA	DME	1.0/2.0/2.0	5	39	17
4	TEA	DME	1.0/2.0/2.0	72	37^c^	—d
5	TEA	DME	1.0/1.5/0.2	18	57	30
6	DMAP	DME	1.0/1.5/0.2	18	61	44
7	DMAP	DME	1.0/2.0/0.2^b^	18	78	23
8	DMAP	DMF	1.0/1.5/0.2	18	78	79
9	DMAP	DMF	1.0/1.5/0.2	5	83	31
10	DMAP	NMP	1.0/1.5/0.2	18	99	—d
11	TZ/DBU^a^	DMF	1.0/2.0/2.0	5	99	28

a 1:1 molar ratio.

b Reaction performed at 80 °C.

c Side reaction evident in ^19^F‐NMR spectrum.

d Product was insoluble and radical concentration could not be measured.

Notably, **P1‐2** and **P2‐2** exhibited poor solubility under all reaction conditions, consistent with observations from PPM reactions involving nitroxide radicals and either **P1** or **P2** [12]. The radical attachment reported in Table 2 are for the isolated soluble fraction. **P2‐2** exhibited excellent radical stability in a sealed capillary (Figure S35A); however, when stored in ambient conditions in an unsealed capillary, ~45% decomposition was observed over 41 days (Figure S35B). Fully insoluble verdazyl‐ORPs were often generated during the optimization process. Radical incorporation was not possible with Evans method for these samples. The anecdotal observation that insoluble polymers tend to be bright red suggests a correlation between higher radical loading and decreased solubility of the system synthesized. This trend was substantiated by Hansen solubility parameter modeling using a group contribution model [69], which predicted a significant increase in the Hansen distance (*R*_*a*_) between the polymer and common solvents (DMSO, DMF, THF, chloroform, and DMAc) as the molar fraction of radical pendant groups increased (Table S4) [70, 71]. While unmodified **P2** had an estimated solubility parameter of 23.9 MPa^0.5^, this increased to 28.1 MPa^0.5^ when calculated for a statistical copolymer of **P2‐2** containing 75% radical pendant groups (Table S3). The reduced solubility observed in **P1‐2** and **P2‐2** is likely driven by intermolecular hydrogen bonding between the secondary amide N─H donors and carbonyl acceptors [72], potentially acting together with π–π stacking interactions to physically cross‐link the polymer chains [73]. Maintaining a degree of solubility was a key objective to enable electrochemical studies, and as such, higher contents were not pursued.

#### 2.2.2 Optimization of Transesterification PPM With Radical Nucleophile 3

Reaction conditions for the synthesis of **P1‐3** via transesterification of poly(pentafluorophenyl methacrylate) **P1** with hydroxymethyl radical **3** are summarized in Table 3. The modification of **P1** proved generally inefficient, with various reaction conditions yielding only 20%–28% radical incorporation regardless of the Lewis base catalyst, solvent, molar equivalents of catalyst, and radical nucleophile. Guided by literature precedents identifying DMF as an accelerant for PFP ester transesterification [45], solvent optimization was limited to DME and THF, which showed minimal change in polymer radical density (23%–24%), comparable to results obtained using DMF (20%, Table 3; entries 1, 5–6). Variations in radical molar equivalents (Table 3; entries 1–3) and Lewis base loading (Table 3; entry 4) resulted in no significant deviation in functionalisation. The only notable improvement (28%) was achieved using a stoichiometric equivalent of DMAP (1.0 equivalents, Table 3; entry 8). The reduction in reactivity relative to those observed in the aminolysis PPM used to synthesize the **P1‐2** and **P2‐2** analogs, is attributed to the lower nucleophilicity of the hydroxymethyl group (**3**) compared to the aminomethyl (**2**), combined with the steric hindrance of the polymer backbone, and follows trends seen in other PPM reactions using **P1** and **P2** [12, 43, 45]. DMAP promoted slightly higher rates of hydrolysis (as determined by ^19^F‐NMR) compared to TEA (35% vs. 25%, respectively, Table 3; entries 4 and 8). While no conditions yielded significantly higher radical density, all **P1‐3** polymers remained fully soluble, unlike the high‐loading ester aminolysis analogs (**P1‐2**). While maximizing the density of pendant radical groups is a primary objective in ORP design, this must be balanced against the requirement for control over polymer solubility. The superior solubility of **P1‐3** relative to the amide‐linked analogs (**P1‐2** and **P2‐2**) is likely attributable to two factors. First, the transesterification linkage in **P1‐3** lacks the secondary amide protons responsible for intermolecular hydrogen bonding, likely partially responsible for the insolubility of **P1‐2** and **P2‐2** [72]. In addition, the lower radical density in **P1‐3** reduces the frequency of interchain π–π stacking interactions [73], thereby suppressing physical cross‐linking.

**TABLE 3:** Postpolymerization conditions for the optimization of the synthesis of **P1‐3**. Reactions were performed at 50 °C unless otherwise stated.

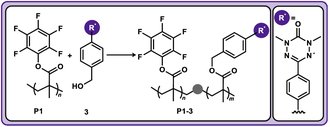					
Entry	Base	Solvent	**[Polymer]/** **[radical]/** **[base]**	PFP reacted, %	Evans method, %
1	TEA	DMF	1.0/1.5/2.0	64	20^a^
2	TEA	DMF	1.0/1.0/2.0	52	25^a^
3	TEA	DMF	1.0/2.0/2.0	65	20
4	TEA	DMF	1.0/1.5/1.0	49	24
5	TEA	DME	1.0/1.5/2.0	50	24
6	TEA	THF	1.0/1.5/2.0	38	23
7	DMAP	DMF	1.0/1.5/2.0	70	24
8	DMAP	DMF	1.0/1.5/1.0	63	28^a^

a Averaged from triplicate experiments.

Reaction conditions for the synthesis of **P2‐3** via transesterification of poly(pentafluorophenyl acrylate) **P2** with hydroxymethyl radical **3** are summarized in Table 4. Consistent with previous observations for **P2**, product precipitation was frequently observed. Replicating the optimized conditions for **P1‐3** yielded an insoluble polymer (Table 4; entry 1). Similarly, employing high DMAP loadings (2.0 equivalents) in DMF resulted in insolubility (Table 4; entry 2), while lower loadings (0.5 equivalents) yielded negligible functionalisation (3%, Table 4; entry 3). Attempts to drive the reaction with reduced DMAP (0.2 equivalents) at 80 °C improved radical concentration on the polymer to 19%, with significant PFP ester cleavage also observed via ^19^F‐NMR (Table 4; entry 4), indicating a competitive hydrolysis reaction is occurring. Using DME with DMAP (2.0 equivalents) yielded a soluble polymer, with low radical incorporation (14%, Table 4; entry 5). Alternative solvents (DME and THF) paired with TEA similarly yielded insoluble products (Table 4; entries 6–9). However, the specific combination of TEA as the base and DMF as the solvent proved critical for maintaining product solubility. Using 2.0 equivalents of TEA with 1.5 equivalents of **3** yielded a soluble polymer with 36% radical attachment (Table 4; entry 10). Optimization of radical molar excess revealed that decreasing the radical loading to 1.0 equivalent reduced functionalisation (28%, Table 4; entry 11), while unexpectedly, increasing the loading to 2.0 equivalents caused a sharp drop in radical concentration to 6% (Table 4; entry 12). Finally, catalyst optimization demonstrated that lowering the TEA loading was beneficial; while 1.0 equivalent resulted in a slight decrease (Table 4; entry 13), reducing the TEA loading to 0.5 equivalents achieved the highest radical concentration for **P2‐3** (39.0%), with minimal side reactions observed via ^19^F‐NMR (Table 4; entry 14). Preliminary stability studies indicated that **P2‐3** exhibits excellent stability. The EPR signal showed no decomposition over 40 days in a sealed capillary (Figure S36). Furthermore, the ability of **3** to undergo two reversible one‐electron processes supports that **P2‐3** may also be suited to bipolar redox polymers in flow systems [37].

**TABLE 4:** Postpolymerization conditions for the optimization of the synthesis of **P2‐3**. Reactions were performed at 50 °C unless otherwise stated.

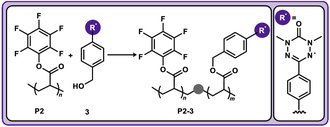					
Entry	Base	Solvent	**[Polymer]/** **[radical]/** **[base]**	PFP reacted, %	Evans method, %
1	DMAP	DMF	1.0/1.5/1.0	67	—b
2	DMAP	DMF	1.0/1.5/2.0	68	—b
3	DMAP	DMF	1.0/1.5/0.5	70	3
4	DMAP	DMF	1.0/1.2/0.2^a^	85	19
5	DMAP	DME	1.0/1.5/2.0	61	14
6	TEA	DME	1.0/1.5/2.0	34	—b
7	TEA	DME	1.0/2.0/2.0	75	—b
8	TEA	DME	1.0/1.5/1.0	48	—b
9	TEA	THF	1.0/1.5/2.0	31	—b
10	TEA	DMF	1.0/1.5/2.0	43	36^d^
11	TEA	DMF	1.0/1.0/2.0	36	28
12	TEA	DMF	1.0/2.0/2.0	56	6^c^
13	TEA	DMF	1.0/1.5/1.0	61	26
14	TEA	DMF	1.0/1.5/0.5	44	39^d^

a Reaction at 80 °C for 18 h.

b Product was insoluble and radical concentration could not be measured.

c Averaged from triplicate experiments.

d Averaged from duplicate experiments.

### 2.3 Synthesis of Verdazyl‐ORPs

Three previously unreported verdazyl‐ORPs were prepared using the optimized PPM reactions. The compounds synthesized encompass diverse properties including radical density, solubility, and accessible electrochemical processes (**P2‐2**, **P1‐3**, and **P2‐3**). To provide sufficient material for the physical and electrochemical characterization, both ester aminolysis and transesterification pathways were scaled up, affording approximately 100 mg of each target polymer.

The **P2‐2** polymer had a high radical functionalisation (75%, Evans method) and PFP ester consumption (85% by ^19^F‐NMR), consistent with the optimized conditions (Table 2). Solid‐state EPR spectroscopy confirmed the presence of the radical pendants, displaying a single broad signal at *g* = 2.0025 (Figure 4A). Room‐temperature solid‐state measurements for samples with weak hyperfine couplings are expected to exhibit broader line groups, as was observed [74]. When compared to the unbound radical **2**, the solid‐state spectra showed further signal broadening due to increased radical–radical interactions between the polymer pendants [12, 19, 34]. FTIR spectroscopy supports the efficient consumption of PFP groups, with a significant reduction in the **P2** ester carbonyl stretch at 1780 cm^−1^ observed (Figure 4B). Consistent with **P2‐2**, the FTIR spectra contained a new amide carbonyl stretch (1681 cm^−1^) and a single broad peak associated with the N─H stretching (~3500 cm^−1^). Minimal hydrolysis was observed in the FTIR with no stretches attributable to carboxylic acid formation. Thermogravimetric analysis (TGA) (Figure S38) revealed a decrease in thermal stability for **P2‐2** (*T*_onset_ = 127.5 °C) compared with the unmodified polymer **P2** (*T*_onset_ = 262.5 °C), consistent with the presence of thermally less stable pendant radical groups (*T*_onset_(**2**) = 227.5 °C) [75]. The derivative thermogravimetric curve (Figure S39) showed that the maximum rate of mass loss occurred at a higher temperature (*T*_max_ = 410.0 °C) relative to both **P2** (*T*_max_ = 365.0 °C) and **2** (*T*_max_ = 287.5 °C), with **P2‐2** exhibiting a moderate residual mass of 23.0% at 800 °C. A careful balance of higher radical loading and sufficient solubility for electrochemical characterization was required, and therefore our focus moved toward ORPs **P1‐3** and **P2‐3** due to the polymers enhanced solubility.

**FIGURE 4:**
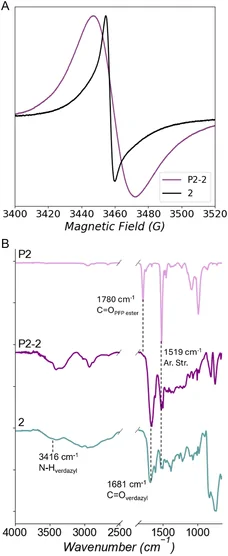
(A) The first derivative X‐band (CW) EPR spectrum (ν   =   9.69 GHz) of solid **P2‐2** within a glass capillary and solid **2** in a glass capillary at 298 K. A field modulation of 2.1 G and power of 15 mW (12 dB attenuation) were used. (B) The FTIR spectra of **P2‐2, P2,** and radical **2.**

The transesterification PPM of **P1** with **3** was conducted using the optimized conditions established for **P1‐3** (Table 3). Interestingly, while the total PFP ester cleavage (31% by ^19^F‐NMR) was significantly lower than that observed during optimization (63%), the radical functionalisation remained comparable (26.6% vs. 27.7%) and supported a reduction in hydrolysis upon scale‐up. The EPR spectrum of **P1‐3** at room temperature in degassed chloroform is shown in Figure 5A. The spectrum comprised an 11(+2) broadened line group, consistent with moderate radical–radical interactions between pendant groups [12, 19], and can be simulated using hyperfine coupling constants *a*(N_2,4_) = 6.1 G, *a*(N_1,5_) = 5.4 G, and *a*(H) = 5.2 G. This degree of broadening was less than that typically observed for highly functionalized ORPs, where a significant signal broadening is expected [12, 19]. FTIR analysis supported polymer functionalisation, through the reduction of the PFP ester carbonyl stretch (1780 cm^−1^) and the appearance of a new band at 1685 cm^−1^, attributed to the amide present in **3** (Figure 5B). Minimal hydrolysis was observed in the spectra and is consistent with ^19^F‐NMR, which confirmed the presence of residual PFP ester groups. The ^1^H‐NMR spectrum exhibited broad aromatic resonances consistent with the introduction of the new aromatic pendant groups of **3** (Figure S11). TGA demonstrated that **P1‐3** had reduced thermal stability when compared with **P1**. The onset of decomposition (*T*_onset_ = 207.5 °C) occurred earlier than that of the polymer **P1** (*T*_onset_ = 267.5 °C), which was attributed to the lower stability of the pendant radicals **3** (*T*_onset_ = 237.5 °C) (Figure S40). The derivative thermogravimetric curve revealed that the maximum rate of mass loss occurred at a higher temperature (*T*_max_ = 337.5 °C), approaching that of the unmodified **P1** (*T*_max_ = 385.0 °C), corresponding to decomposition of unmodified polymer segments within **P1‐3** (Figure S41).

**FIGURE 5:**
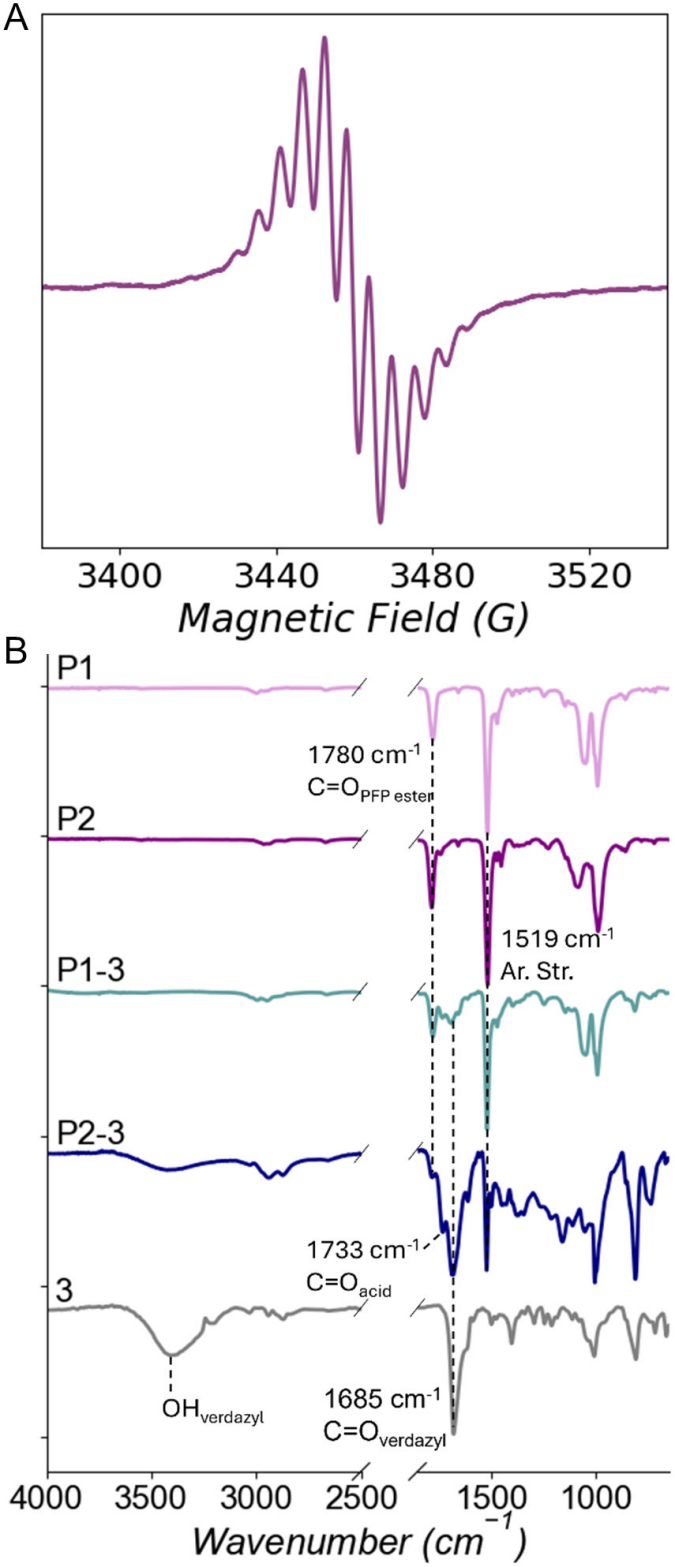
(A) The first derivative X‐band (CW) EPR spectrum (*ν *= 9.69 GHz) of the soluble portion of **P1‐3** in degassed chloroform within a glass capillary at 298 K. A field modulation of 2.1 G and power of 15 mW (12 dB attenuation) was used. (B) The FTIR spectra of **P1‐3** and **P2‐3** in comparison with **P1**, **P2** and radical **3.**

**P2** was reacted with **3** using the protocol identified for **P2‐3** in Table 4 (entry 14), enabling a direct comparison with **P2‐2,** which was synthesized via ester aminolysis. Replication of the optimal conditions at a larger scale gave **P2‐3** with radical functionalisation comparable to the small‐scale reaction (40% vs. 39% radical concentration). A greater amount of hydrolysis was observed, indicated by the difference between the Evans method and quantitative ^19^F‐NMR (61% of PFP ester groups reacted, determined using ^19^F‐NMR). The EPR spectrum contained the characteristic 11 + 2 line group of a verdazyl radical, with slight line broadening, consistent with the weak radical–radical interactions expected at low radical loading (Figure S34) [12, 19]. Spectral simulation have hyperfine coupling constants of *a*(N_2,4_) = 6.5 G, *a*(N_1,5_) = 5.7 G, and *a*(H_R_) = 4.8 G. The FTIR spectrum of **P2‐3** shows the expected reduction in intensity of the PFP ester carbonyl stretch at 1780 cm^−1^, and addition of carbonyl stretches associated with attachment of **3** (Figure 5B). This observation was more pronounced than that seen in **P1‐3**, consistent with the higher radical incorporation achieved in **P2‐3**. The **P2‐3** spectrum also exhibits a carbonyl stretch at 1733 cm^−1^, associated with the carboxylic acid group formed during the hydrolysis side reaction in the PPM, as also observed by quantitative ^19^F‐NMR. The thermal stability of **P2‐3** was compared with that of **P2** and **3** using TGA (Figure S40). The modified polymer exhibited an earlier onset of decomposition (*T*_onset_(**P2‐3**) = 205.0 °C) than the unmodified **P2** (*T*_onset_(**P2**) = 262.5 °C), consistent with the presence of thermally less stable radical pendant groups (*T*_onset_(**3**) = 237.5 °C). The thermal decomposition was much more gradual in **P2‐3** when compared to **P1‐3** (Figure S41), likely attributed to the greater radical concentration of **P2‐3**.

## 3 Electrochemical Measurements of Verdazyl‐ORPs

Verdazyl‐ORP composite films were prepared from a slurry consisting of **P2‐3** (50 mg), a conductive carbon black additive (200 mg), and polyvinylidene fluoride (PVDF) (50 mg). Subsequently, the mixture was blade‐coated from NMP and assembled in coin cells using ethylene carbonate:diethylene carbonate (EC:DEC, 1:1, v/v) and LiPF_6_ (1 M) as the electrolyte. A low verdazyl content (16.7 wt%) combined with a high carbon black loading (66.7 wt%) was intentionally employed to maximize electronic percolation and ensure accurate electrochemical characterization of the radical processes. The 7π verdazyl redox chemistry in a nonaqueous electrolyte is well established (Figure ‍6) [54], with the observed cathode (p‐type) electrode behavior likely underpinning the performance of the fabricated coin cell.

**FIGURE 6:**
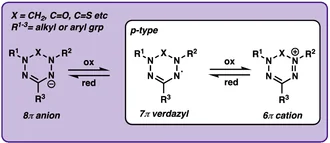
The bipolar redox behavior of a 7π verdazyl in a nonaqueous electrolyte [54]. The neutral radical can be oxidized to the 6π cation and reduced to the 8π anion. Pendant radicals in this work likely support the cathode (p‐type) process.

A reversible redox process in the fabricated coin cell was observable using CV at ~3.43 V versus Li/Li^+^ (Figure 7A). This is likely associated with the oxidation of the 7π verdazyl to the 6π cation, as in radical **3,** the one‐electron oxidation process occurs at ~0.25 V versus Fc/Fc^+^ (Figure 3B), which corresponds to ~3.50 V versus Li/Li^+^. While the large carbon black loading leads to a dominant non‐Faradaic capacitive background, giving the CV a quasi‐rectangular shape, a distinct and reversible faradaic redox process was still observable. This confirms the fundamental electrochemical accessibility of the polymer‐bound verdazyl radicals despite the highly resistive nature of the coin cell. The one‐electron reduction of the 7π radical to the 8π radical anion, expected at approximately 2.0 V versus Li/Li^+^ (corresponding to the −1.2 V versus Fc/Fc^+^ wave observed in the solution state CV, Figure 3B), was not observed in this coin cell configuration. This is consistent with previous studies on verdazyl radicals, which demonstrate that the reduction process exhibits vastly different voltammetry depending on the solvent environment. Specifically, the electron‐rich 8π verdazyl anion is inherently much less stable than the 6π cation and displays notably sluggish electrochemical kinetics [66, 76]. While the 8π anion is stable in the noncoordinating dichloromethane environment used for solution‐state testing, generating this highly nucleophilic species in the presence of electrophilic carbonate solvents (EC:DEC) may induce irreversible side reactions. Furthermore, any faint faradaic signal from this kinetically sluggish reduction is completely obscured at these low potentials by the dominant capacitive background current of the carbon additive. The peak current did not increase linearly with the square root of the scan rate (Figure 7B), indicating that the kinetic mechanism at the surface of the modified electrode is not a diffusion‐controlled process. Broadening of the redox wave shape was observed as scan rates increased, indicating sluggish electrochemical kinetics despite the chemical reversibility of the redox process. The observed Δ*E*_*p*_ (~170 mV at 20 mV/s) deviated significantly from the 59 mV expected for an ideal one‐electron process [77]. The reduced charge transport capabilities are likely a result of the low density of the redox‐active pendant groups and the use of an unconjugated polymer backbone, resulting in significant site‐isolation and an increase in the average electron hopping distance between redox centers [78].

**FIGURE 7:**
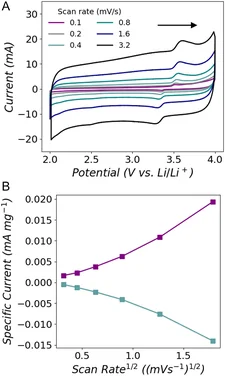
(A) A cyclic voltammogram of the **P2‐3** fabricated coin cell at different scan rates. Voltammograms are plotted (IUPAC) versus Li/Li^+^ (*E*_1/2_ = 0 V). (B) The corresponding plot of peak current at different scan rates for the oxidation (purple) and reduction (blue).

To evaluate the electrochemical behavior at higher active‐material mass fractions, additional cells were prepared using **P2‐2** with an increased verdazyl active material content (60 wt%). The **P2‐2** sample used in these cells was synthesized with slightly lower radical functionalisation (57% vs. 75% maximum achieved for **P2‐2**) to increase solubility for ease of electrode preparation. Electrodes were formulated in a 6:3:1 mass ratio of **P2‐2** (100 mg), carbon black (50 mg), and PVDF binder (15 mg). Interestingly, an additional redox feature centered at ~3.2 V was observed (Figure S50A), which may reflect increased polarization associated with the higher active material loading and reduced carbon black content (30 wt% compared with 66.7 wt%). As with **P2‐3**, peak current analysis indicates that the surface redox process is not diffusion‐controlled (Figure S50B). This increased polarization is further supported by the EIS response (Figure S50C), which exhibits a pronounced low‐frequency impedance rise extending to ~10 kΩ in the imaginary component (−Z″), consistent with distributed interfacial kinetics and transport limitations within the composite electrode. This effect is likely exacerbated by the predominantly insulating nature of the polymer matrix as fewer than 57% of the copolymer units consists of redox‐active verdazyl units (due to likely decomposition). The theoretical specific capacity of the **P2‐2** electrode is calculated to be 34 mAhg^−1^, calculated using the known radical concentration of **P2‐2**, 57%, assuming no decomposition occurred. The initial discharge capacity was found to be 4.0 mAhg^−1^ at 0.1 C (10 h charge), 12% of the theoretical maximum capacity of the electrode material. The polymer loading was 0.71 mg cm^−2^, resulting in a low areal capacity of 2.8 μAh cm^−2^. This low utilization reflects several compounding factors, including partial decomposition of the verdazyl pendant groups prior to electrode preparation, the intrinsically insulating nature of the copolymer, the low fraction of electrochemically active radical sites, and dilution of the active material by the conductive carbon additive. Because the theoretical capacity assumes complete radical retention and full electrochemical accessibility of all verdazyl sites, any degradation or electrically isolated radicals inherently reduce the achievable capacity. Furthermore, GCD profiles exhibit a sloping voltage response, consistent with a substantial double‐layer capacitive contribution from the carbon additive superimposed on the observed molecular redox process. However, when subjected to extended cycling (Figure S50D), the material demonstrated robust stability. After 500 cycles, the discharge capacity retained 80% of its initial value. At a lower charge rate (C/50, Figure S50E), the cell delivered a stable capacity of ~4 mAh g^−1^ over 200 cycles with minimal fade (~2%). This indicates that both the stable capacitive background and the fraction of electronically accessible active material are highly robust during repeated cycling. For this class of materials to transition from a fundamental proof‐of‐concept to practical viability, future architectures must demonstrate near‐quantitative radical incorporation and utilization to achieve specific capacities exceeding typical thresholds of ~100 mAh g^−1^. Furthermore, optimization of the composite matrix is required to permit active material mass loadings above 70 wt% without restricting charge transport.

## 4 Conclusions

In this study, a series of newly developed verdazyl‐based ORPs were synthesized via the PPM of activated PFP ester polymers **P1** and **P2** using functionalized 1,5‐dimethyl‐6‐oxoverdazyl radicals. To overcome the inherent instability of the dimethyl *p*‐aniline‐functionalized 6‐oxoverdazyl (**1**), **2** and **3** were designed with alkyl spacers that eliminate the conjugation between the radical and the nucleophile. Previously unreported verdazyl radical precursors **T2** and **T3** were synthesized using Suzuki–Miyaura palladium‐catalyzed cross‐coupling reactions in high yields (78%–98%). Fundamental electrochemical analysis showed that radical **3** exhibits a cell potential of 1.67 V in conventional organic solvents, representing the highest value reported for a verdazyl radical. The structure not only enhanced radical stability but also facilitated coupling to the polymer backbone through ester aminolysis and transesterification reactions.

The PPM strategy proved versatile, and optimization studies revealed a strong dependence of reaction efficiency on both polymer‐radical pairing and the reaction conditions used. Consistent with nucleophilicity trends, the ester aminolysis pathway (**P2‐2**) achieved the highest functionalisation (75%), whereas the transesterification pathway (**P2‐3**) yielded lower radical loadings. Crucially, this work highlighted a significant trade‐off between polymer radical density and solubility, with insolubility prevalent in polymers supporting high radical attachment, likely from strong π–π stacking of the verdazyl pendant groups or intramolecular hydrogen bonding in the case of **2**. As a fundamental proof‐of‐concept, electrochemical testing was performed on **P2‐2** and **P2‐3**, revealing a chemically reversible redox couple at *E°* = 3.43 V versus Li/Li^+^. While radical degradation and the highly resistive polymer matrix inherently limit the Faradaic capacity, the emergence of this redox couple successfully validates the electrochemical accessibility of the polymer‐bound verdazyl radical. Ultimately, this work establishes a synthetic framework for accessing verdazyl ORPs, providing a foundation for the future design of verdazyl‐based redox‐active polymers.

## 5 Experimental

### 5.1 General Experimental

All reactions were carried out under aerobic conditions with magnetic stirring unless otherwise stated. All reaction temperatures refer to bath temperatures. All reactions involving heating were placed in a room temperature oil bath. All reagents were purchased from Sigma–Aldrich, Combi‐Blocks, AK Scientific, Oakwood, or Alfa Aesar and used without further purification, unless otherwise stated. Solvents were purified using standard laboratory procedures. TLC was performed on Silicycle silica gel 60 F254 precoated aluminum sheets. Visualization of developed plates was achieved through the use of a 254 or 365 nm UV lamp or 2,2‐dihydroxyindane‐1,3‐dione (Ninhydrin) stain. Flash column chromatography was performed using silica gel as supplied by Merck (chromatographic silica particle size: 40–63 μm). Lithium disks (15.6 mm diameter × 0.45 mm thickness), aluminum foil (16 µm thickness), polypropylene (PP) separator (Celgard, 29 µm thickness), conductive Super P carbon black (CB, ≈40 nm), 2032 coin cell casings, springs, and spacers were purchased from mKube Enterprise Pty Ltd.

### 5.2 Physical Measurements

Nuclear magnetic resonance spectra were recorded using either a Bruker Avance III NMR spectrometer operating at 400 MHz (^1^H) or 100 MHz (^13^C) or 376 MHz (^19^F) or on a Bruker Avance III NMR spectrometer operating at 600 MHz (^1^H), 150 MHz (^13^C), or 565 MHz (^19^F) at 300 K. All NMR spectra were calibrated to residual solvent signals. Data are reported as follows: chemical shift (ppm), integration, multiplicity (s = singlet, d = doublet, t = triplet, q = quartet, m = multiplet, and br = broad), and coupling constant (Hz). Infrared spectra were recorded using an attenuated total reflectance PerkinElmer Spectrum 100 FT‐IR with a diamond stage. EPR spectra of the resultant molecules were recorded on a Bruker MicroESR Standard V3.0 (high modulation amplitude) and a Bruker E500 X‐band spectrometer equipped with an ER 4103TM resonator (low modulation amplitude measurements) at room temperature (298 ± 1 K). For all EPR measurements, the radicals were prepared as 1–5 mM degassed (nitrogen) chloroform, tetrahydrofuran, or toluene solutions. Spectra were simulated using the EasySpin package in MATLAB [63]. The garlic function was used to simulate an isotropic fast‐motion cw EPR spectrum with a Nelder–Mead minimization algorithm used to optimize. CV of unbound radicals was recorded at room temperature using a Metrohm Autolab PGSTAT101 and data acquired with Metrohm Autolab, Nova 2.0 software. Experiments involved a CH Instruments glassy carbon working electrode, Pt wire counter electrode, and leakless Ag/AgCl reference electrode. HRMS was performed by electrospray ionization and direct infusion into an LTQ‐Orbitrap XL mass spectrometer using a syringe pump. The accurate mass was calibrated and measured at a 30,000 resolution. Prior to sample analysis, mass accuracy was assessed using a standard compound (Formoterol, MW 345.1814) with a mass accuracy acceptance limit of <2.5 ppm. Mass analyses were carried out in positive ionization mode on a Waters Xevo TQ triple quadrupole mass spectrometer (Waters Corporation, Manchester, United Kingdom) or by infusion into an Orbitrap Q‐Exactive HF mass spectrometer equipped with a heated electrospray ionization (HESI) source, where data were acquired over the scan range of 150–2000 *m*/*z* in positive ionization mode at a resolution of 240,000. Analyses were performed by Dr. David Nichols or Dr. Richard Wilson of the Central Science Laboratory at the University of Tasmania. GPC analyses were performed on an Agilent Infinity 1260 GPC system, with a triple detector suite (refractive index, light scattering, and viscosity), two PLgel MIXED‐C columns in a column oven set to 30 °C, and tetrahydrofuran as eluent (flow rate 0.8 mL min^–1^). All polymer samples were dissolved for at least 12 h prior to analysis in the eluent at approximately 5 mg mL^–1^ and then filtered through a 450 nm nylon filter. The system was calibrated with poly(methyl methacrylate) (PMMA) standards with molecular weights of 400‍–‍1,944,000 g mol^–1^. TGA was performed using a NETZSCH STA 449 F5 Jupiter. Samples (~5 mg) were loaded in an alumina crucible and heated from 25 to 800 °C at a rate of 10 °C per min in a nitrogen atmosphere.

### 5.3 Fabrication of Verdazyl Polymer Electrodes

Working electrodes were prepared by first dry‐mixing the active material (100 mg) with carbon black (50 mg) using a mortar and pestle for 15 min. The powder blend was then combined with PVDF binder (15 mg) in NMP (3 mL) and mixed using a high‐shear mixer (Ultra Turrax T18, 5000 rpm, 1 h) to obtain a homogeneous slurry (active material:carbon black:PVDF = 6:3:1 by mass). The slurry was cast onto an aluminum foil current collector (9 µm thickness, mKube Enterprise Pty Ltd.) using a blade coater with a 100 µm gap and dried in air at room temperature overnight. The areal loading of active material on the final dried electrode was 0.71 mg cm^−2^. Electrode disks (9 mm diameter) were punched from the dried films and vacuum‐dried at 120 °C prior to cell assembly.

### 5.4 Glovebox Assembly of Verdazyl Half Coin Cells

Electrode disks were transferred into an argon‐filled glovebox with O_2_ and H_2_O levels <0.1 ppm. The electrolyte solution was a solution of 1 M LiPF6 in EC and DEC. Starting with the positive terminal case, the electrode disk was placed and electrolyte (70 µL) was added dropwise onto the electrode surface. Following this, a polypropylene separator (mKube Enterprise Pty Ltd, 29 µm thickness) was placed gently on top, followed by a 15.6 mm Li metal chip (mKube Enterprise Pty Ltd, 450 µm thickness), spacer, and spring were then also placed in order. Lastly, the negative terminal case was placed, and the cell subsequently crimped to ensure structural integrity. Following an overnight stabilization period, the assembled cell was then used for electrochemical testing.

### 5.5 Electrochemical Testing of Verdazyl Half Cells

Battery tester (Neware A211) and potentiostat (Biologic VMP‐300) were used to examine the specific capacity at various C‐rates, long‐term cycling, voltage versus specific capacity, CV curves, and electrochemical impedance spectroscopy (EIS) data. Galvanostatic charge/discharge (GCD) was conducted using a Neware battery tester (BTS8000), with cycling stability monitored for upto 500 cycles at C/10. The voltage range was between 2.0 and 4.0 V. The selected current for GCD was based on the theoretical capacity of verdazyl portion (74 mAh g^−1^). Specific capacity of the samples was calculated from the discharge curves using the formula Q=∫Idt/m, where *Q* is the specific capacity (in mAh g^−1^), *I* is the current applied and *m* is the mass of the active material [79, 80]. The areal capacity (*Q*_a_, mAh cm^−2^) was calculated as $Q_{a}=Q*L$, where *Q* is the specific capacity (mAh g^−1^) and *L* is the active material loading (g cm^−2^). Moreover, the Coulombic efficiency (CE) is known as ratio between the discharge and charge capacities of the same cycle. Hence, CE provides the ratio between total Li‐ion insertion into anode material and total Li‐ions back to cathode material. CV and EIS were conducted on a Bio‐Logic SP‐300 station. CV of half cells was performed at various scan rates (0.1–6.4 mV/s) with a voltage range of 2.0–4.3 V for 3 cycles each. EIS measurements were performed over a frequency range of 100 kHz–100 mHz using a single sine alternative current (AC).

### 5.6 Syntheses

**6‐(4‐Aminophenyl)‐2,4‐dimethyl‐1,2,4,5‐tetrazinan‐3‐one (T1)** **.**Under a nitrogen atmosphere, 2,4‐dimethyl‐6‐(4‐nitrophenyl)‐1,2,4,5‐tetrazinan‐3‐one (0.51 g, 2.04 mmol) was added to a two‐necked 250 mL flask. Ethanol (~60 mL) was added to a flask and sparged with nitrogen gas for 10 min. PtO_2_ (0.02 g, 0.07 mmol, 3%) was added, and the reaction mixture was placed under a hydrogen balloon for 5 h. Then, the mixture was filtered through a plug of celite, and all solvents were removed from the filtrate under reduced pressure to give **T1** as a crude brown solid. Yield: 94% (0.45 g, 2.03 mmol). IR (ATR *ν_max_*/cm^−1^): 3343 (NH str.), 3218 (NH str.), and 1591 (C═O str.). ^1^H‐NMR(600 MHz, DMSO*‐d*_6_): *δ* 7.13 (2H, d, *J = *8.4 Hz), 6.52 (2H, d, *J* = 8.4 Hz), 5.44 (2H, d, *J = *9.1 Hz), 5.06 (2H, s, 2H), 4.67 (1H, t, *J* = 9.1 Hz), 2.93 (6H, s) ppm. ^13^C‐NMR(150 MHz, DMSO*‐d*_6_): *δ* 154.6, 148.4, 127.5, 123.3, 113.4, 68.9, 37.7 ppm. HRMS(ESI): [M + H]^+^ calcd. for C_10_H_16_N_5_O 222.1349; found 222.1348 [51].

5.7

**1,5‐Dimethyl‐3‐(*p*‐aniline)‐6‐oxoverdazyl (1). T1** (0.21 g, 0.95 mmol) was dissolved in MeOH (~20 mL) containing Na_2_CO_3_ (2 M, 0.8 mL) in a 50 mL flask. The mixture was stirred, and a solution of K_3_[(Fe(CN)_6_)] (0.8944 g, 2.72 mmol) in H_2_O (4.4 mL) was added dropwise. The reaction mixture was stirred for 5 min at room temperature. A solution of brine (20 mL) and water (20 mL) were added to the reaction mixture and were immediately extracted with Et_2_O (3 × 40 mL). The organic extracts were combined and washed with a brine solution (50 mL), dried over MgSO_4,_ and filtered. The solvent was removed under pressure to give a red‐brown oil. Yield: 53% (0.11 g, 0.50 mmol). IR (ATR *ν_max_*/cm^−1^): 3355 (NH str.) and 1669 (C═O). UV‐vis (MeCN): *λ*_max_ = 284 nm (*ε*_max_ = 20,500 Lmol^−1^cm^−1^, π → π^∗^), *λ*_1_ = ∼520 nm. Retention factor (RF) (1:1 EtOAc:hexanes) = 0.50. ESR (toluene, 298 K): 11 + 2 line group *a*(N_2,4_) = 6.5 G; *a*(N_1,5_) = 5.2 G; *a*(H) = 4.9 G (6H); *g* = 2.0030. HRMS (ESI): [M + H]^+^ calcd. C_10_H_12_N_5_O 218.1042; found 218.1042 [51].

5.8

**6‐(4'‐(Aminomethyl)‐[1,1′‐biphenyl]‐4‐yl)‐2,4‐dimethyl‐1,2,4,5‐tetrazinan‐3‐one (T2).** Under a nitrogen atmosphere, 6‐(4‐bromophenyl)‐2,4‐dimethyl‐1,2,4,5‐tetrazinan‐3‐one (0.87 g, 3.04 mmol) was added to a two‐necked 100 mL flask connected to a condenser. n‐Propanol (~45 mL), Na_2_CO_3_ (1 M, 30 mL, 10 equivalents), and (4‐(aminomethyl)phenyl)boronic acid hydrochloride (0.68 g, 3.65 mmol) were added, and the solution was sparged with nitrogen gas for 10 min. Pd(PPh_3_)_4_ (22 mg, 0.63%) was added to the solution, and the reaction was heated at reflux. After ~18 h, the solution was cooled to room temperature, and the aqueous and organic phases were separated. The organic phase was passed through a Celite plug and concentrated. The crude product was dissolved in HCl (0.04 M, 100 mL) and extracted with 3 × 20 mL of DCM. The aqueous phase was basified with ~1 mL of saturated Na_2_CO_3_ and re‐extracted with 3 × 30 mL of n‐butanol. The organic extracts were combined, dried with MgSO_4,_ and filtered. The solvent was evaporated under reduced pressure, giving the product as an off‐white solid. Yield: 78% (0.74 g, 2.36 mmol). IR (ATR *ν_max_*/cm*^−^* ^1^): 3358 (br, NH), 3217 (NH), 2928, 1586 (C═O).^1^H‐NMR(600 MHz, DMSO*‐d*_6_) *δ* 7.68–7.61 (6H, m) 7.46 (2H, d, *J = *7.6 Hz), 5.79 (2H, d, *J = *7.5 Hz), 4.96 (1H, t, *J = *7.4 Hz), 3.83 (2H, s), and 2.97 (6H, s) ppm. ^13^C‐NMR(150 MHz, DMSO*‐d*_6_) *δ* 154.5, 143.5, 139.7, 137.7, 135.7, 131.5, 128.7, 127.7, 126.4, 68.4, 45.4, and 38.2 ppm. HRMS (ESI): [M + H]^+^ calcd. for C_17_H_21_N_5_O, 312.1819; found 312.1815.

5.9

**1,5‐Dimethyl‐3‐(4‐methylamine‐(1,1′‐biphenyl))‐6‐oxoverdazyl (2). T2** (0.11 g, 0.34 mmol) was dissolved in 1:1 MeCN/0.05 2M acetic acid (~10 mL) in a 20 mL flask and heated to 50 °C while stirring. NaIO_4_ (0.81 g, 3.77 mmol) was added, and the reaction mixture was heated to 50 °C for 10 min. The solution was extracted with 1 × 20 mL DCM. The organic phase was washed with a saturated Na_2_CO_3_ solution (1 × 10 mL), dried over MgSO_4,_ and filtered. The solvent was removed from the filtrate under reduced pressure, yielding a bright‐red solid. Yield: 81% (0.084 g, 2.74 mmol). IR (ATR *ν_max_*/cm^−1^): 3441 (br, NH), 3070, 2939, and 1677 (C═O). ESR (CHCl_3_, 298 K): 11 + 2 line group *a*(N_2,4_) = 6.4 G; *a*(N_1,5_) = 5.3 G; *a*(H) = 5.3 G (6H); *g* = 2.0019. RF (10% MeOH/EtOAc + 5% NH_3_) = 0.41. HRMS (ESI‐TOF) *m*/*z* [M• – NH_2_]^+^ calcd. for C_17_H_16_N_4_O: 292.1324; found 292.1321.

5.10

**6‐(4′‐(Hydroxymethyl)‐[1,1′‐biphe** **nyl]‐4‐yl)‐2,4‐dimethyl‐1,2,4,5‐tetrazinan‐3‐one (T3).** Under a nitrogen atmosphere, 6‐(4‐bromophenyl)‐2,4‐dimethyl‐1,2,4,5‐tetrazinan‐3‐one (1.27 g, 4.46 mmol) was added to a two‐necked 100 mL flask connected to a condenser. n‐Propanol (~60 mL), (4‐(hydroxymethyl)phenyl)boronic acid (0.76 g, 5.00 mmol), Na_2_CO_3_ (1 M, 20 mL, 20.00 mmol), and Pd(PPh_3_)_4_ (0.023 g, 0.45%) were added to the solution. The solution was heated at reflux for 18 h and, upon completion, cooled to room temperature before filtering through a celite plug. The solvent was evaporated under reduced pressure, and the resulting solid was triturated with water. The resulting off‐white powder was collected by vacuum filtration and used without further purification. Yield: 94% (1.31 g, 4.19 mmol). IR (ATR *ν_max_/*cm^−1^): 3319 (br, OH), 3198 (NH), 2881, 1613 (C═O). ^1^H‐NMR(600 MHz, DMSO*‐d*_6_) *δ* 7.70–7.60 (6H, m) 7.42 (2H, d, *J = *7.9 Hz), 5.76 (2H, d, *J = *7.6 Hz), 5.25 (1H, bs), 4.97 (1H, t, *J = *7.4 Hz), 4.55 (2H, d, *J = *4.4 Hz), 2.98 (6H, s) ppm. ^13^C‐NMR(150 MHz, DMSO*‐d*_6_) *δ* 155.0, 142.3, 140.1, 138.6, 136.3, 128.1, 127.5, 126.8, 126.8, 68.8, 63.1, and 38.2 ppm. HRMS (ESI): [M + Na]^+^ calcd. for C_17_H_20_N_4_O_2_ 335.1478; found 335.1466.

5.11

**1,5‐Dimethyl‐3‐(4‐hydroxymethyl‐(1,1′‐biphenyl))‐6‐oxoverdazyl (3). T3** (0.46 g, 1.46 mmol) was dissolved in MeOH (45 mL) and stirred at room temperature in a 250 mL flask, followed by the dropwise addition of a solution containing K_3_[Fe(CN)_6_] (2.56 g, 7.77 mmol) in water (16 mL) and a saturated Na_2_CO_3_ solution (1.0 mL). The mixture was stirred at room temperature for 10 min. H_2_O (45 mL) and brine (45 mL) were added to the reaction mixture, and the resultant red precipitate was filtered out. The crude precipitate was washed with water and hexane. The red solid was passed through a silica plug using 4:6 hexanes:EtOAc as eluent (RF = 0.4) and concentrated under reduced pressure, giving a bright red solid. Yield: 44% (0.20 g, 0.65 mmol). IR (ATR *ν_max_* */*cm^−1^): 3412 (br, OH) and 1679 (C═O). ESR (toluene, 298 K): 11 + 2 line group *a*(N_2,4_) = 6.4 G; *a*(N_1,5_) = 5.3 G; *a*(H) = 5.0 G (6H); *g* = 2.0025. HRMS (ESI‐TOF) *m*/*z* [M + H]^+^ calcd. for C_17_H_18_N_4_O_2_: 310.1430; found 310.1422.

5.12

**Poly(pentafluorophenyl methacrylate) (P1).** Under a nitrogen atmosphere, pentafluorophenyl methacrylate (4.18 g, 16.59 mmol) was added to a 50 mL flask containing 1,4‐dioxane (16 mL), 4‐cyano‐4‐(phenylcarbonothioylthio)pentanoic acid (0.047 g, 0.17 mmol), and 4,4‐azobis(4‐cyanovaleric acid) (0.023 g, 0.082 mmol). DMF (100 μL) was added as an internal ^1^H‍‐‍NMR standard. The solution was sparged with nitrogen for 30 min and heated to 90 °C for 6 h. The reaction mixture was concentrated to ~50% of its volume and precipitated from ice cold MeOH (10× excess). The resulting pink‐white precipitate was centrifuged, collected, and reprecipitated twice from DCM/MeOH. The pink‐white solid was centrifuged and collected. Yield: 42% (1.74 g). The molecular weight distribution was obtained by GPC in triple‐filtered THF. PD = 1.38, *M*_n_ = 10,993 g/mol. ^1^H‐NMR(600 MHz, CDCl_3_): *δ* 2.44 (2H, br), 1.48 (3H, br). ^19^F‍‐‍NMR(376 MHz, CDCl_3_): *δ* −151 (2F, br), −157 (1F, br), and −162 (2F, br) ppm [43].

5.13

**Poly(pentafluorophenyl acrylate) (P2).** Under a nitrogen atmosphere, pentafluorophenyl acrylate (2.00 g, 8.40 mmol) was added to a 20 mL flask containing 1,4‐dioxane (8 mL), 2‐(butylthiocarbonothioylthio)propanoic acid (0.020 g, 0.084 mmol), and 4,4‐azobis(4‐cyanovaleric acid) (0.012 g, 0.044 mmol). DMF (65 μL) was added as an internal ^1^H‐NMR standard. The solution was sparged with nitrogen for 20 min and heated to 90 °C for 100 min. The reaction mixture was concentrated to ~50% of its volume and precipitated from ice cold MeOH (10× excess). The resulting white precipitate was centrifuged, collected, and reprecipitated twice from DCM/MeOH. The white solid was centrifuged and collected. Yield: 65% (1.29 g). The molecular weight distribution was obtained by GPC in triple‐filtered THF. PD = 1.22 and *M*_n_ = 8,610 g/mol. ^1^H‐NMR(600 MHz, CDCl_3_): *δ* 3.09 (1H, br) and 2.11 (2H, br) ppm. ^19^F‐NMR(376 MHz, CDCl_3_): *δ* −153 (2F, br), −157 (1F, br), −162 (2F, br) ppm [43].

5.14

**General Method for PPM Reactions.** Under a nitrogen atmosphere, **P1** or **P2** (~18 mg) was added to solvent (~0.5 mL) with radical and catalyst at various loadings. The reaction was stirred for varying times and temperatures. Upon completion, trifluorotoluene (2 μL) was added to the solution as a ^19^F‐NMR standard, and ^19^F‐NMR of the reaction mixture was collected using a CDCl_3_ capillary. The reaction mixture was then precipitated in methanol (~15 mL), centrifuged, and the polymer was collected. Once dried, the sample was dissolved, and the radical concentration was determined using the Evans method [57].

5.15

**P2‐2.** Under a nitrogen atmosphere, **P2** (0.124 g) was added to a flask containing DMF (1.2 mL), **2** (0.25 g, 0.81 mmol), and DMAP (0.014 g, 0.11 mmol). The reaction mixture was stirred at 50 °C for 18 h. Upon completion, the solution was cooled to room temperature, and trifluorotoluene (6 μL) was added as a ^19^F‐NMR standard. The reaction mixture was precipitated in methanol (~40 mL), centrifigued, and the supernatant discarded. The polymer was reprecipitated from DCM and methanol twice and subsequently dried under reduced pressure to give a bright red solid. Yield 0.13 g. 75% radical concentration (Evans method: 0.6 mg **P2‐2** in 0.25 mL of DMF). 85% PFP cleavage (^19^F‐NMR).

5.16

**P1‐3.** Under a nitrogen atmosphere, **P1** (0.10 g) was added to a flask containing DMF (1.0 mL), **3** (0.19 g, 0.61 mmol), and DMAP (0.05 g, 0.41 mmol). The reaction mixture was stirred at 50 °C for 18 h. Upon completion, the solution was cooled to room temperature, and trifluorotoluene (6 μL) was added as a ^19^F‐NMR standard. The reaction mixture was precipitated in methanol (~40 mL) and centrifuged, and the supernatant was discarded. The polymer was reprecipitated from DCM and methanol twice and subsequently dried under reduced pressure to give a brown solid. Yield: 0.067 g. 27% radical concentration (Evans method: 3.2 mg **P1‐3** in 0.25 mL THF). 29% PFP cleavage (^19^F‐NMR). ^1^H‍‐‍NMR(600 MHz, CDCl_3_): *δ* 7.36 (br), 4.68 (br), 3.65 (br), 3.42 (br), 2.34 (br), and 1.31 (br). ^19^F‐NMR(376 MHz, CDCl_3_): *δ* −151 (2F, br), −157 (1F, br), −162 (2F, br) ppm. ESR (CHCl_3_, 298 K): 11 + 2 line group *a*(N_2,4_) = 6.1 G; *a*(N_1,5_) = 5.4 G; *a*(H) = 5.2 G; *g* = 2.0028.

5.17

**P2** **‐** **3.** Under a nitrogen atmosphere, **P2** (0.10 g) was added to a flask containing DMF (1.0 mL), **3** (0.21 g, 0.68 mmol), and TEA (30 μL, 0.22 mmol). The reaction mixture was stirred at 50 °C for 18 h. Upon completion, the solution was cooled to room temperature, and trifluorotoluene (6 μL) was added as a standard prior to ^19^F‐NMR studies. The reaction mixture was precipitated in methanol (~40 mL) and centrifuged, and the supernatant discarded. The polymer was reprecipitated from DCM and methanol twice and subsequently dried under reduced pressure to give a red solid. Yield: 0.095 g. 41% radical concentration (Evans method: 3.2 mg **P2‐3** in 0.25 mL of acetone). 61% PFP cleavage (^19^F‐NMR). ESR (THF, 298 K): 11 + 2 line group *a*(N_2,4_) = 6.5 G; *a*(N_1,5_) = 5.7 G; *a*(H) = 4.8 G; *g* = 2.0017.

## Author Contributions

**Theo A. Ellingsen:** writing – original draft, validation, investigation, methodology, data curation, visualization, formal analysis. **James D. Randall** **:** writing – review and editing, investigation, formal analysis, data curation. **Bhagya Dharmasiri** **:** methodology, data curation; **Wesley J. Olivier** **:** investigation. **Luke C. Henderson** **:** supervision, funding acquisition. **Melissa K. Stanfield:** writing – review and editing, formal analysis, supervision. **Stuart C. Thickett:** writing – review and editing, methodology, formal analysis, supervision, conceptualization. **Rebecca O. Fuller** **:** writing – review and editing, methodology, formal analysis, supervision, funding acquisition, project administration, conceptualization.

## Acknowledgments

T.A.E. acknowledges the provision of a University of Tasmania Graduate Research Scholarship (TGRS). S.C.T. acknowledges the Australian Research Council for the provision of a Future Fellowship (FT220100096). The authors gratefully acknowledge Deakin University, this research was conducted with support from the Australian Research Council Industry Transformation Research Hub (IH210100023) and an ARC Mid‐Career Industry fellowship (IM230100048) for L.C.H. We gratefully acknowledge the University of Tasmania Central Science laboratory for providing access to mass spectrometry and NMR spectroscopy services.

Open access publishing facilitated by University of Tasmania, as part of the Wiley ‐ University of Tasmania agreement via the Council of Australasian University Librarians.

## Funding

This work was supported by Australian Research Council (FT220100096, IH210100023, IM230100048).

## Conflicts of Interest

The authors declare no conflicts of interest.

## Supporting information

The authors have cited additional references within the Supporting Information [49, 51, 53, 57, 61, 63, 69, 70, 81].

## Data Availability

The data supporting this article have been included as part of the Supporting Information.
