# Supplementary material for: Redox‐Active Verdazyl Radical Polymers via Postpolymerization Modification of Active Ester Polymers
Source: Chempluschem. 2026 Oct 27;91(11):e70269. doi: 10.1002/cplu.70269 (PMC13713106; doi:10.1002/cplu.70269)
Supplement: Supplementary file 1 [file CPLU-91-e70269-s001.pdf]

Redox-Active Verdazyl Radical Polymers via Post-Polymerisation Modification of Active Ester Polymers

Theo A. Ellingsen,[a] James D. Randall,[b] Bhagya Dharmasiri, Wesley J. Olivier,[a] Luke C. Henderson,[b] Melissa K. Stanfield,[a] Stuart C. Thickett,[a] and Rebecca O. Fuller,[a†]

[a] School of Natural Sciences – Chemistry, University of Tasmania, Hobart, Tasmania, Australia.
[b] Institute for Frontier Materials, Deakin University, Waurn Ponds, Victoria, Australia
[†]Corresponding Author Email: rebecca.fuller@utas.edu.au

Supporting Information

Table of Contents

**I.**	**Experimental**	**S-2**
**II.**	**NMR Spectra**	**S–6**
**III.**	**EPR spectra**	**S–19**
**IV.**	**Additional Characterisation**	**S–24**
**V.**	**Verdazyl ORP Electrode Characterisation**	**S–32**

I. Experimental

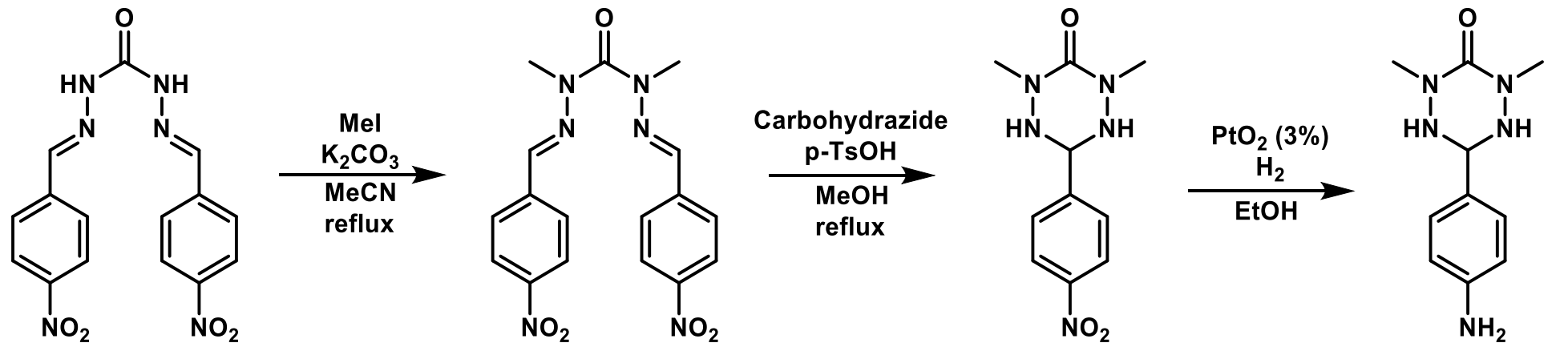

T1

Scheme S1. Synthesis of tetrazinanone **T1**

Carbo-di(*N′*-4-nitrobenzylidene-*N*-methylhydrazide)

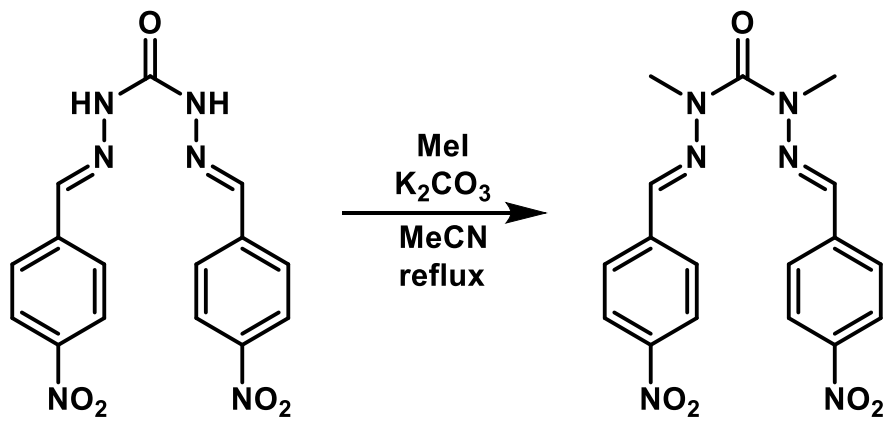

Under a nitrogen atmosphere, Carbo-di-(*N′*-4-nitrophenylhydrazide) (2.09 g, 5.86 mmol) and K_2CO_3 (7.33 g, 53.02 mmol) were added to a two-neck 500 mL flask connected to a condenser. MeCN (~400 mL) was added and the mixture stirred then heated at reflux to aid dissolution. After 1h, the reaction was cooled (30 min) then iodomethane (4.00 mL, 64.3 mmol) was added to the flask dropwise, and the mixture was heated at reflux. After ~18 h, the solution was cooled and H_2O (400 mL) added to precipitate the crude product. The yellow solid was filtered and washed with further H_2O (~400 mL) and Et_2O (~100 mL) then filtered and dried. Yield 82% (1.84 g, 4.79 mmol). IR (ATR n /cm^{-1}): 1679 (C=O str.), 1515 (NO str.), 1334 (NO str.). ^{1}H-NMR(600 MHz, DMSO-d_6) δ 8.20 (4H, d, J = 8.2 Hz), 8.08 (2H, s), 7.85 (4H, d, J = 8.1 Hz), 3.45 (6H, s) ppm. ^{13}C-NMR(150 MHz, DMSO-d_6) δ 157.2, 147.1, 141.6, 136.3, 127.4, 124.0, 33.4 ppm. HRMS (ESI): $[M + H]^+$ calcd. for $C_{17}H_{17}N_6O_5$ 385.1255; found 385.1253.[1]

2,4-dimethyl-6-(4-nitrophenyl)-1,2,4,5-tetrazinan-3-one

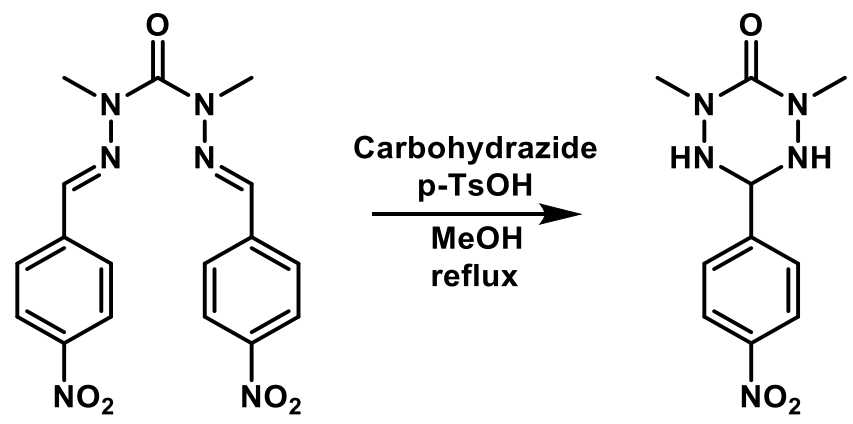

Carbo-di(*N′*-4-nitrobenzylidene-*N*-methylhydrazide) (3.49 g, 9.09 mmol), *p*-toluenesulfonic acid monohydrate (1.84 g, 10.71 mmol) and carbohydrazide (1.31 g, 14.59 mmol) were added to MeOH (90 mL) in a 250 mL flask, and the reaction mixture was heated at reflux for ~17 h. After being removed from the heat, the reaction solution was left undisturbed for 3 hours, and the resulting precipitate was filtered. A saturated solution $NaHCO_3$ (100 mL) diluted with H_2O (100 mL) was added to the filtrate, then the crude extracted with ethyl acetate (EtOAc) (3 x 40 mL). The organic layer was further washed with a brine (100 mL), dried over $MgSO_4$ and all solvents removed from the filtrate under reduced pressure to give the product as a yellow powder. Yield 77% (1.77 g, 7.05 mmol). IR (ATR n /cm^{-1}): 3229 (NH str.), 1631 (C=O str.), 1521 (NO str.), 1345 (NO Str.). ^{1}H-NMR(400 MHz, DMSO-d_6): δ 8.25 (2H, d, J = 8.8 Hz), 7.82 (2H, d, J = 8.7 Hz), 5.96 (2H, d, J = 5.9 Hz), 5.11 (1H, t, J = 5.8 Hz), 2.93 (6H, s) ppm. ^{13}C NMR (150 MHz, DMSO-d_6) δ 154.2, 147.1, 145.0, 128.7, 123.4, 67.7, 37.5 ppm. [2,3]

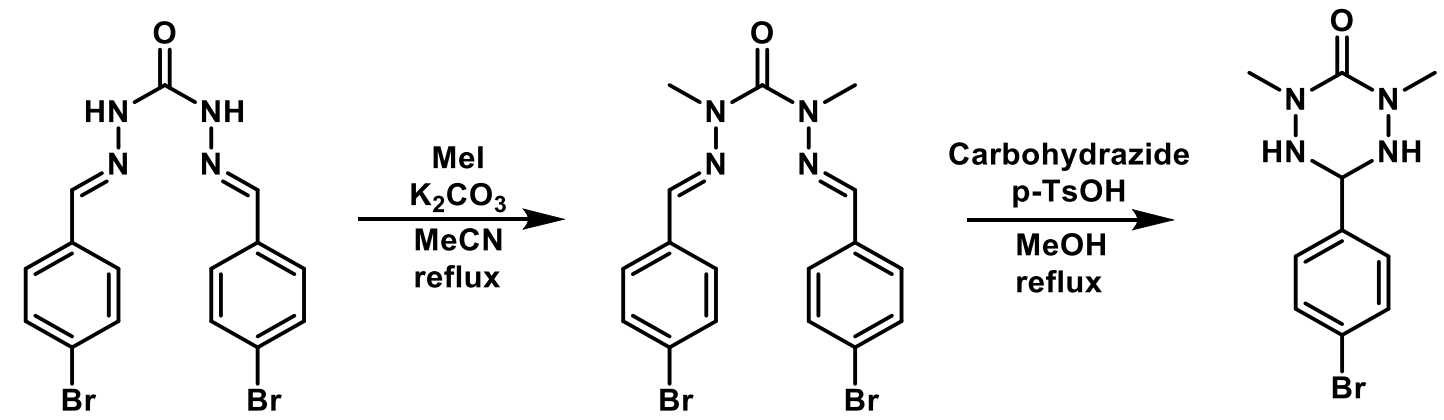

Scheme S2. Synthesis of 6-(4-bromophenyl)-2,4-dimethyl-1,2,4,5-tetrazinan-3-one substrate for cross coupling reactions to synthesise tetrazinanones **T2** and **T3**.

Carbo-di-(*N'*-4-bromophenylhydrazide)

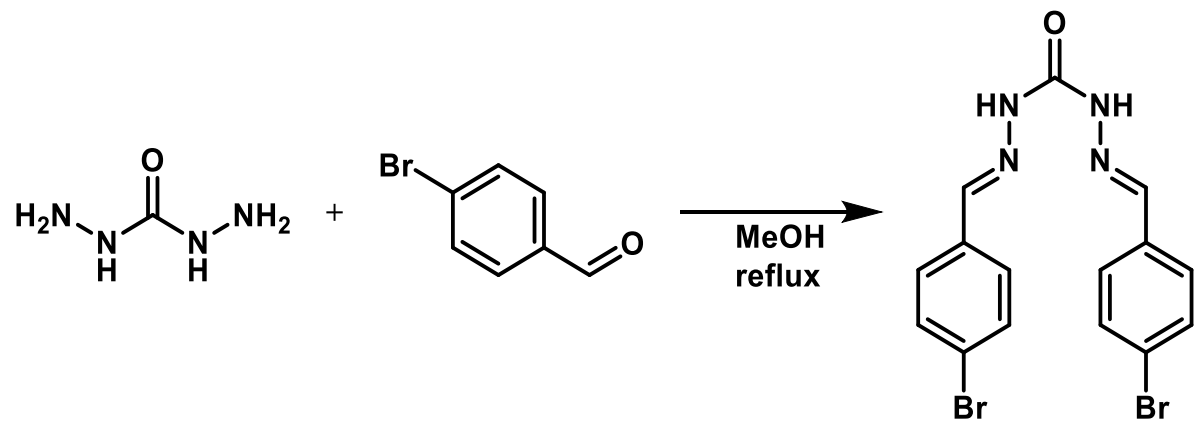

Carbohydrazide (4.49 g, 4.98 mmol) and *p*-bromobenzaldehyde (16.28 g, 8.80 mmol) were combined in MeOH (400 mL) then heated at reflux in a 500mL flask. After 4 h, the mixture was cooled, and the precipitate was filtered and washed with Et_2O (~100 mL). Carbo-di-(*N'*-4-bromophenylhydrazide) was collected as a pure white solid. Yield 87% (16.27 g, 3.83 mmol). ^1H NMR (600 MHz, DMSO-d_6): δ 10.83 (2H, s), 8.16 (2H, bs), 7.71 (4H, m) 7.64 (2H, d, J = 7.7 Hz) ppm. $^{13}C\{^1H\}$ NMR (150 MHz, DMSO-d_6): δ 152.4, 142.3, 134.4, 132.1, 129.2, 123.0 ppm.[1,4]

Carbo-di(*N'*-4-bromophenylidene-*N*-methylhydrazide)

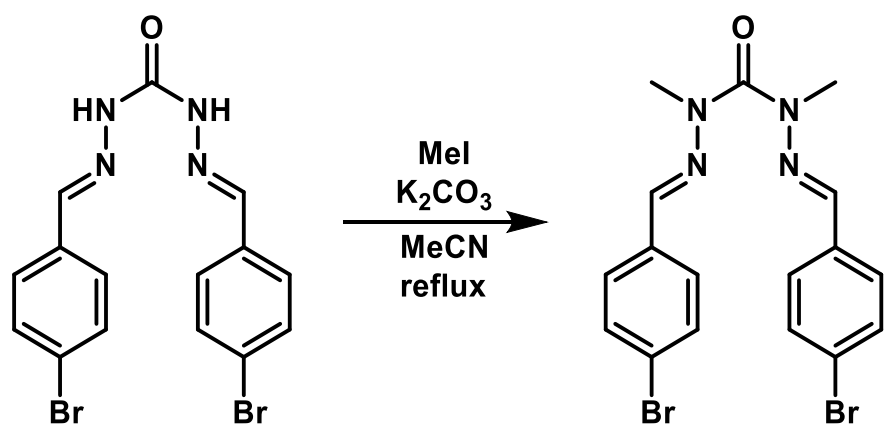

Under a nitrogen atmosphere, carbo-di-(*N'*-4-bromophenylhydrazide (5.19 g, 12.24 mmol) and K_2CO_3 (20.98 g, 0.15 mol) were added to MeCN (400 mL) in a 500 mL two-necked flask connected to a condenser. The solution was stirred at room temperature for 1h. Iodomethane (5 mL, 80.32 mmol) was added dropwise, then the mixture was heated at reflux for ~18 hours. The reaction mixture was filtered while hot, and the filtrate then stored overnight at -18 °C. The white crystalline solid was filtered and washed with hexanes, and subsequently dissolved in CH_2Cl_2. Excess base could be filtered prior to all solvents being removed from the filtrate under reduced pressure and the product was collected as a white powder. Yield 69% (3.84 g, 8.50 mmol). ^1H NMR (600 MHz, DMSO-d_6): δ 7.91 (2H, s), 7.58-7.53 (8H, m), 3.38 (6H, s) ppm. $^{13}C\{^1H\}$ NMR (150 MHz, DMSO-d_6): δ 157.9, 137.5, 135.1, 132.1, 128.9, 122.5, 33.6 ppm.[1]

6-(4-Bromophenyl)-2,4-dimethyl-1,2,4,5-tetrazinan-3-one

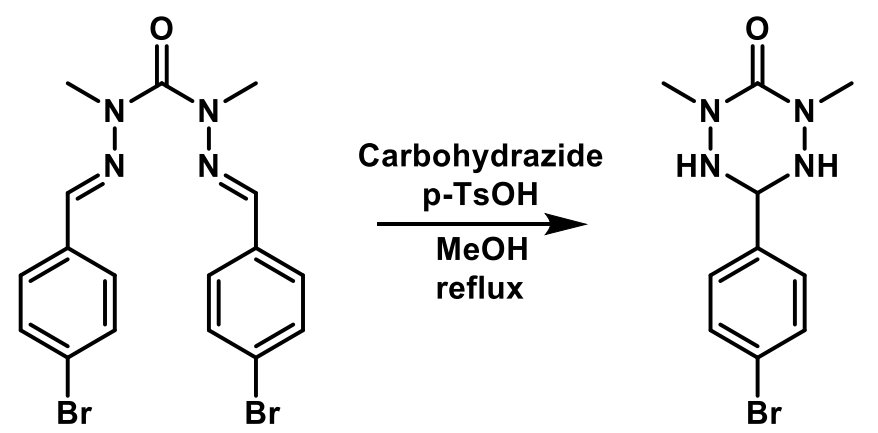

Carbo-di(*N'*-4-bromobenzylidene-*N*-methylhydrazide) (3.71 g, 8.21 mmol), *p*-toluenesulfonic acid monohydrate (2.36 g, 12.41 mmol) and carbohydrazide (0.87 g, 9.69 mmol) were added to MeOH (160 mL) in a 250 mL flask, and the reaction was heated at reflux for ~18 h. After being removed from the heat, the solution was cooled, and the precipitate filtered. The filtrate was concentrated under reduced pressure to ~5 mL, diluted with CH_2Cl_2 (100 mL) and filtered. The filtrate was washed in a separatory funnel with saturated Na_2CO_3 (3 x 50 mL), and brine (1 x 50 mL), before drying over $MgSO_4$ and filtering. The solvent was removed under reduced pressure, and the product collected as a white powder. Yield 80 % (1.87 g, 6.56 mmol) IR (ATR *n* /cm^{-1}): 3243 (NH), 2974, 2924, 2871, 1592 (C=O). ^{1}H-NMR(600 MHz, DMSO-d_6) δ 7.58 (2H, d, J = 7.3 Hz), 7.49 (2H, d, J = 7.9 Hz), 5.77 (2H, d, J = 6.8 Hz), 4.93 (1H, t, J = 6.6 Hz), 2.93 (6H, s) ppm. ^{13}C{^{1}H} NMR (150 MHz, DMSO-d_6): δ 154.8, 136.9, 131.6, 129.9, 121.6, 68.3, 38.1 ppm. HRMS (ESI-TOF) m/z [M + Na]$^+$ calcd. for $C_{10}H_{13}BrN_4ONa$: 309.0144; found 309.0143.

(4-(Pivalamidomethyl)phenyl)boronic acid

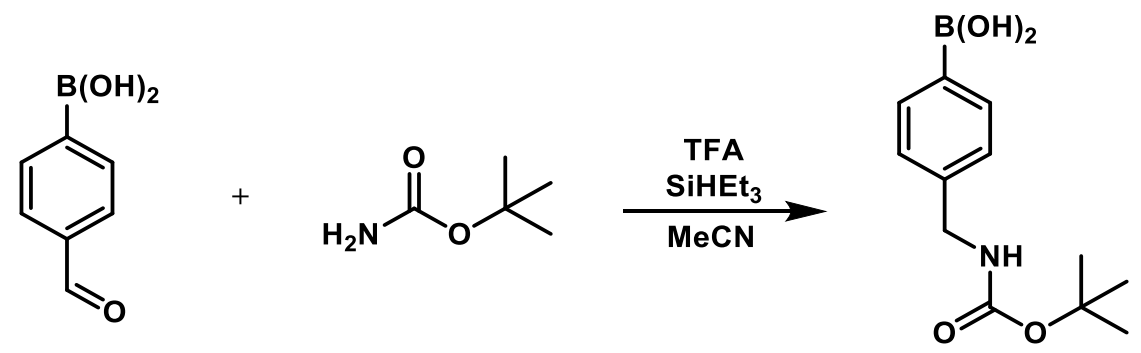

4-Formylphenylboronic acid (6.09 g, 40.6 mmol), tert-butyl carbamate (14.57 g, 124.4 mmol), triethylsilane (16.8 mL, 105.2 mmol), and trifluoroacetic acid (5.2 mL, 68.0 mmol) were stirred in MeCN (140 mL) in a 250 mL flask at 30 ºC for 72 h. The white precipitate was collected, washed with hexanes, and dried yielding the pure product. Yield 65% (6.67 g, 26.6 mmol).). ^{1}H-NMR(600 MHz, DMSO-d_6) δ 8.01 (2H, s), 7.73 (2H, d, J = 6.9 Hz), 7.39 (1H, br), 7.20 (2H, d, J = 7.0 Hz), 4.13 (2H, d, J = 5.2 Hz), 1.40 (9H, s) ppm. ^{13}C-NMR(150 MHz, DMSO-d_6) δ 155.87, 147.07, 134.13, 132.29, 125.95, 77.83, 43.44, 28.29 ppm.

(4-(Aminomethyl)phenyl)boronic acid hydrochloride

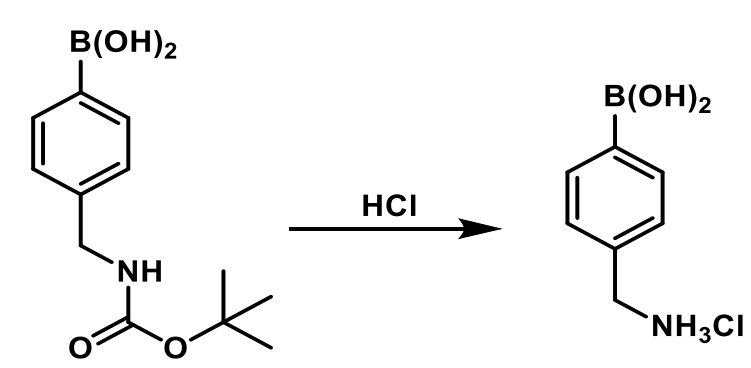

(4-(pivalamidomethyl)phenyl)boronic acid (1.58 g, 6.28 mmol) was stirred in HCl (30 mL, 36%) for 1.5 h. The white precipitate was removed via vacuum filtration, and all solvent was removed from the filtrate under reduced pressure to give the product as free-flowing white solid. Yield 99% (1.16 g, 6.20 mmol). ^{1}H-NMR(600 MHz, DMSO-d_6) δ 8.15 (2H, bs), 7.82 (2H, d, J = 7.1 Hz), 7.45 (2H, d, J = 7.3 Hz), 4.01 (2H, s) ppm. ^{13}C-NMR(150 MHz, D_2O) δ 135.0, 134.3, 132.9, 128.1, 42.9 ppm.[5]

Table S1. Post polymerization conditions for the synthesis of **P1-1**

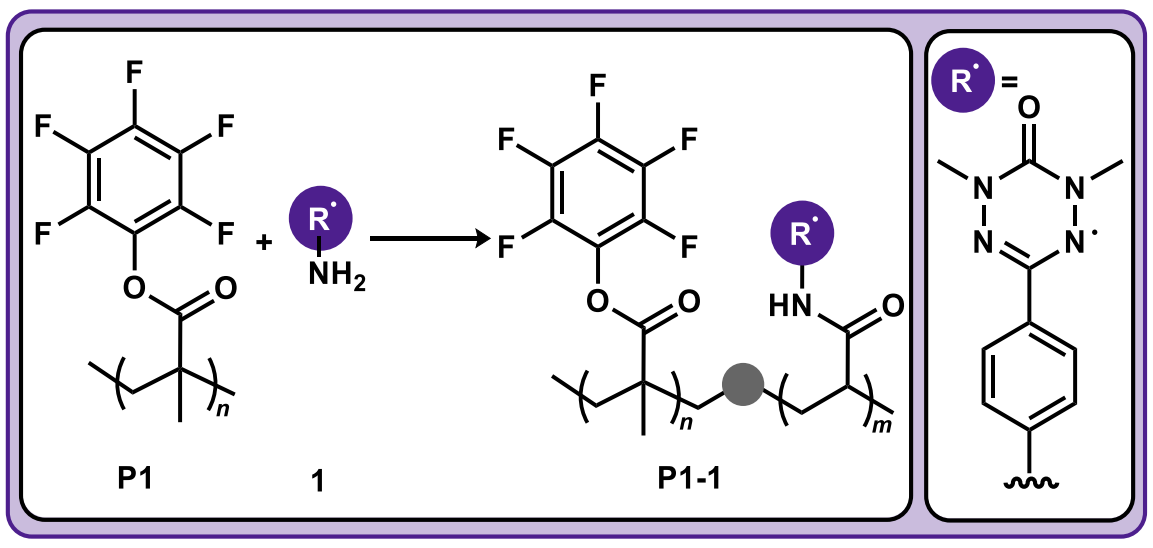

entry	solvent	[polymer]/[radical]	PFP reacted (%)[a]
**1**	THF	1.0/1.5	11
**2**	DMF	1.0/1.5	1
**3**	NMP	1.0/1.5	2
**4**	DME	1.0/1.5	53
**5**	DME	1.0/0.5	5
**6**	DME	1.0/2.0	45

[a] Determined from triplicate experiments

Quantitative ^{19}F NMR

The concentration of polymer pentafluorophenol groups remaining following PPM is determined by quantitative NMR. Briefly, on completion of PPM trifluorotoluene (2 µL) was added to the reaction mixture as a ^{19}F-NMR standard. An aliquot of the reaction mixture was placed in an NMR tube containing a sealed capillary with $CDCl_3$.

Evans Method

Verdazyl-ORP (~5 mg) dissolved in DMF, THF or acetone (250-500 µL) was sealed in a capillary. The capillary was placed in an NMR tube containing $CDCl_3$. The standard was prepared by sealing the same solvent (DMF or THF) within a capillary, and placing this in a second NMR tube also containing $CDCl_3$. Relative chemical shift of the solvent (DMF or THF) is used to determine radical concentration. Full calculations can be found in previous work.[6]

II NMR Spectra

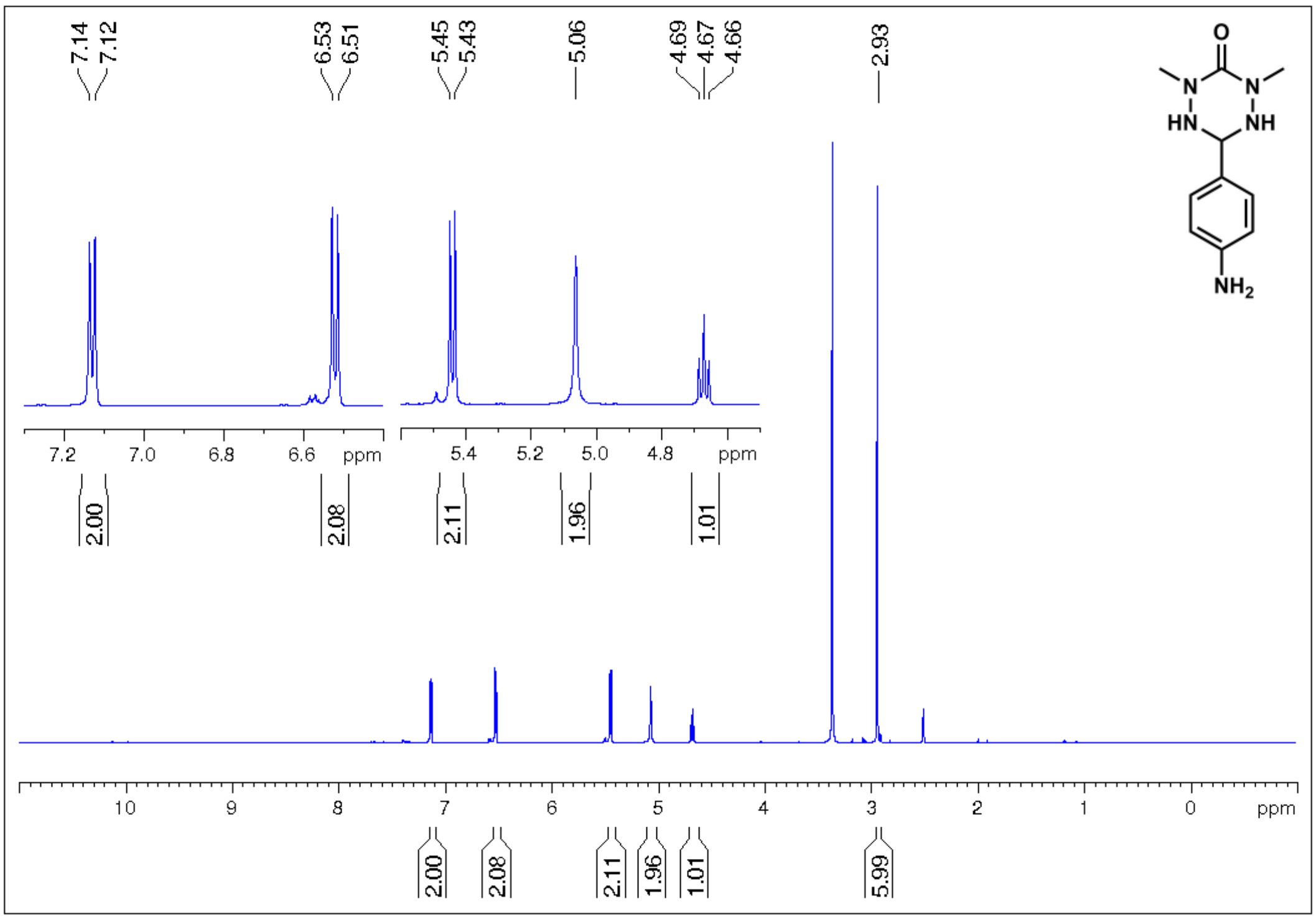

Figure S1: ^{1}H NMR (600 MHz, DMSO-d_6) of **T1**.

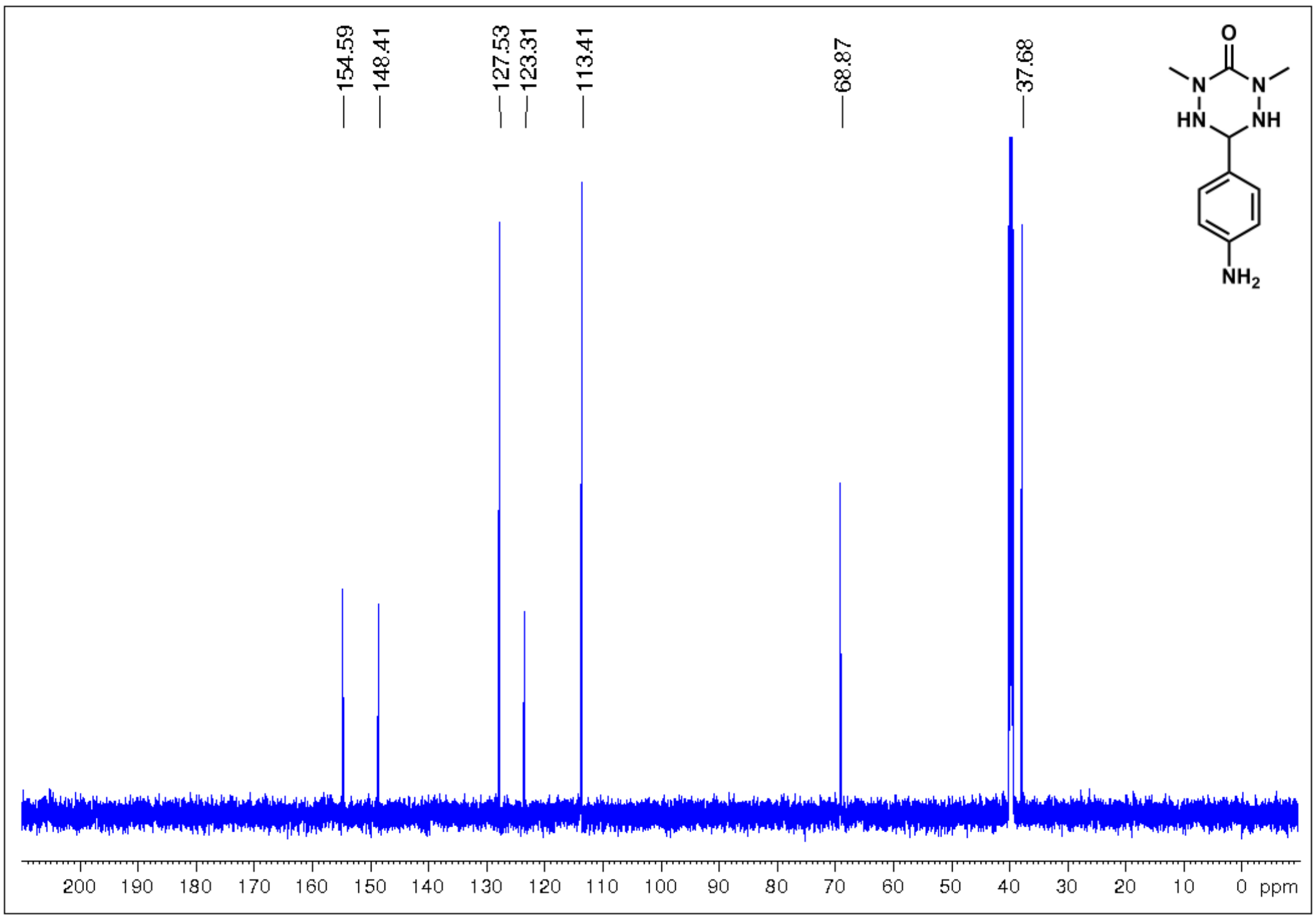

Figure S2: ^{13}C{^{1}H} NMR (150 MHz, DMSO-d_6) of **T1**.

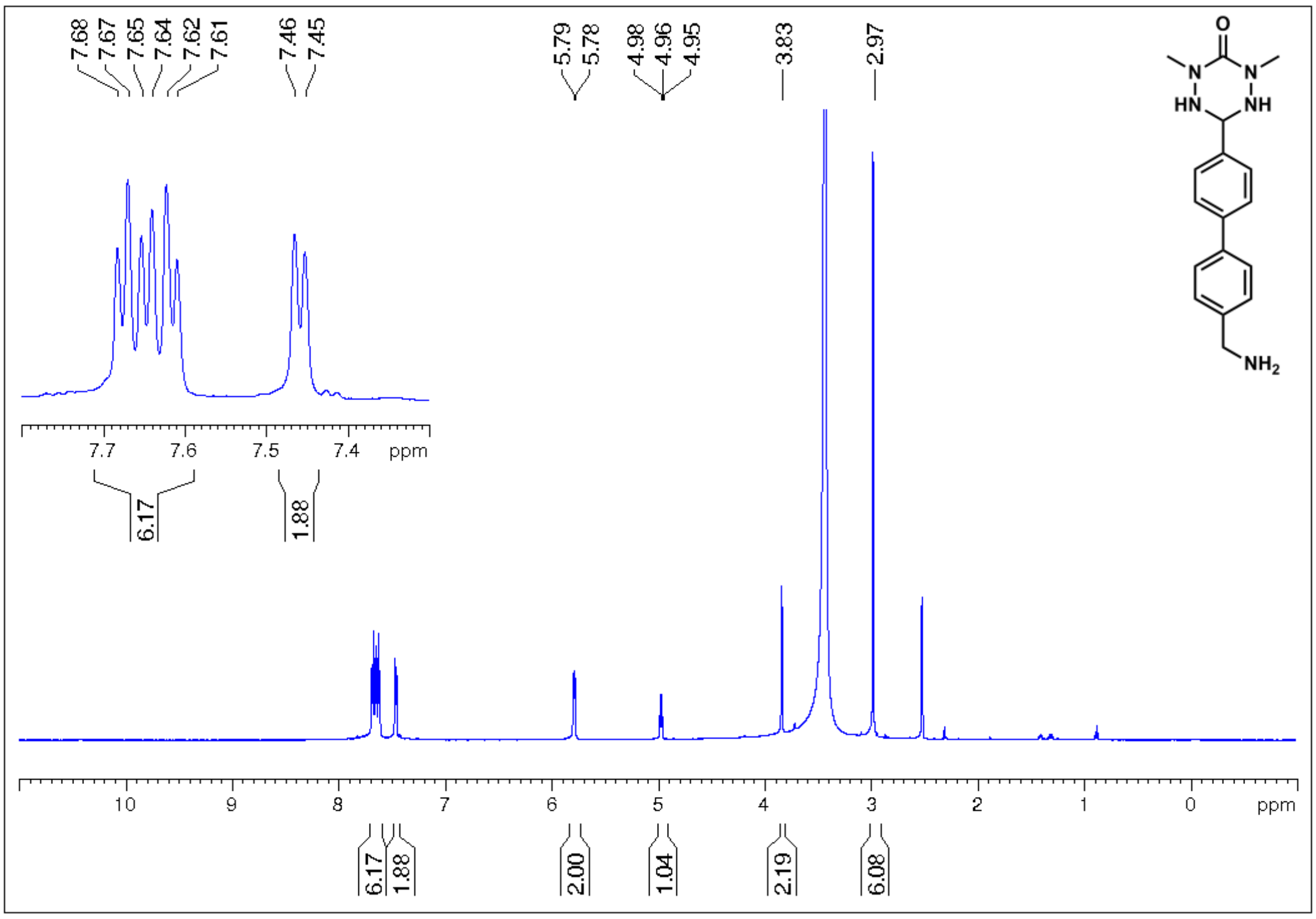

Figure S3: ^{1}H NMR (600 MHz, DMSO-d_6) of **T2**.

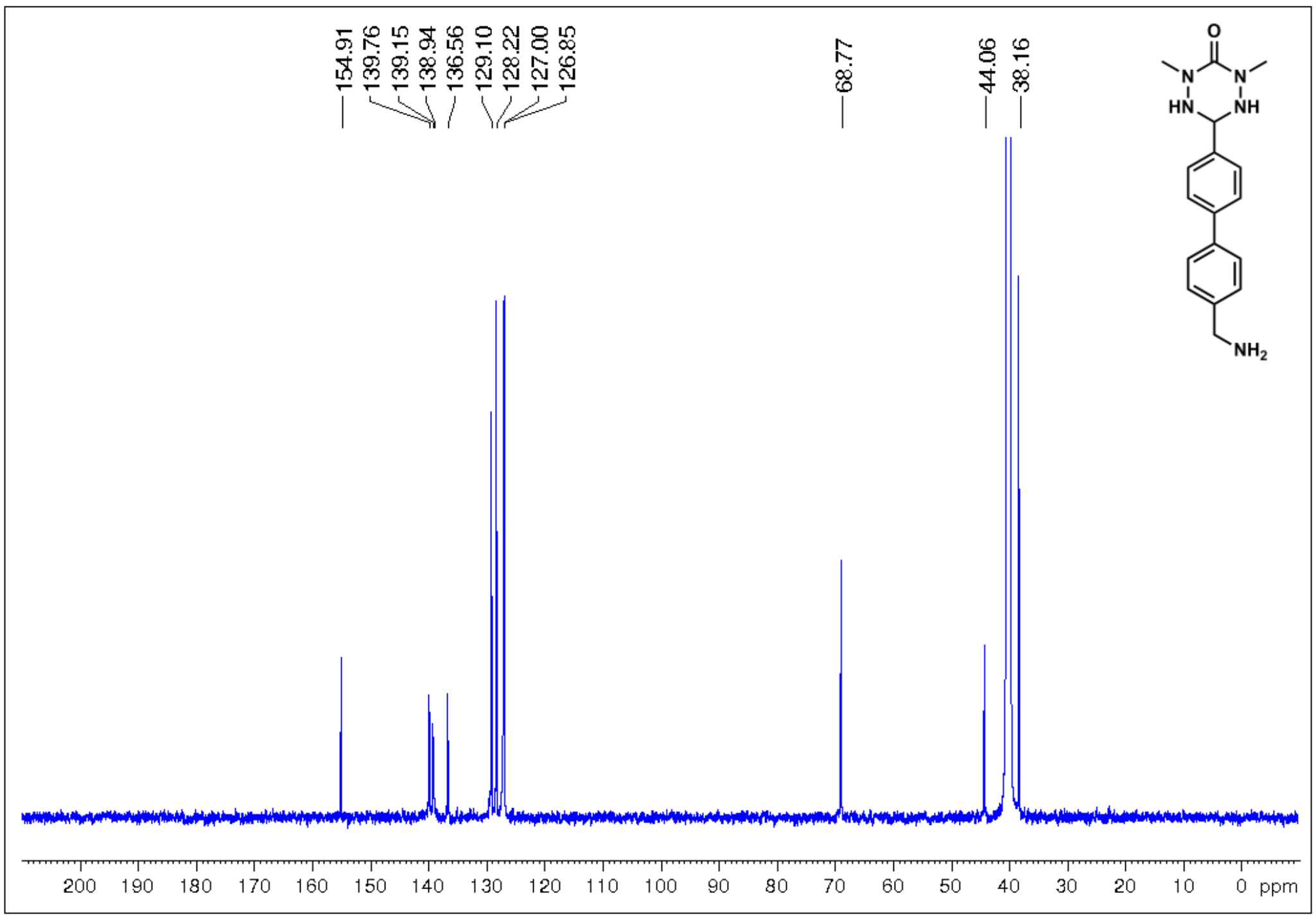

Figure S4: $^{13}C\{^{1}H\}$ NMR (150 MHz, DMSO-d_6) of **T2**.

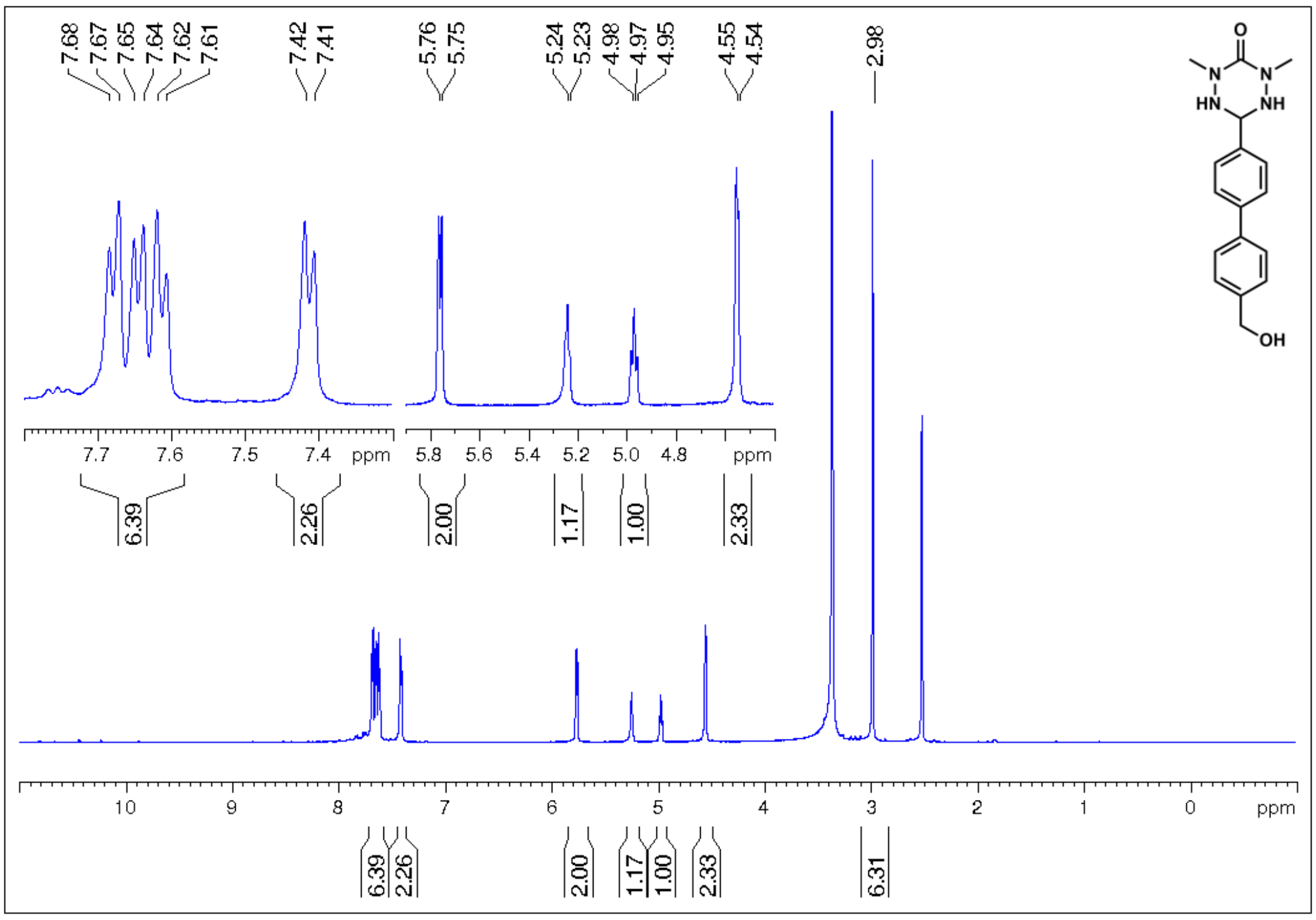

Figure S5: ^{1}H NMR (600 MHz, DMSO-d_6) of **T3**.

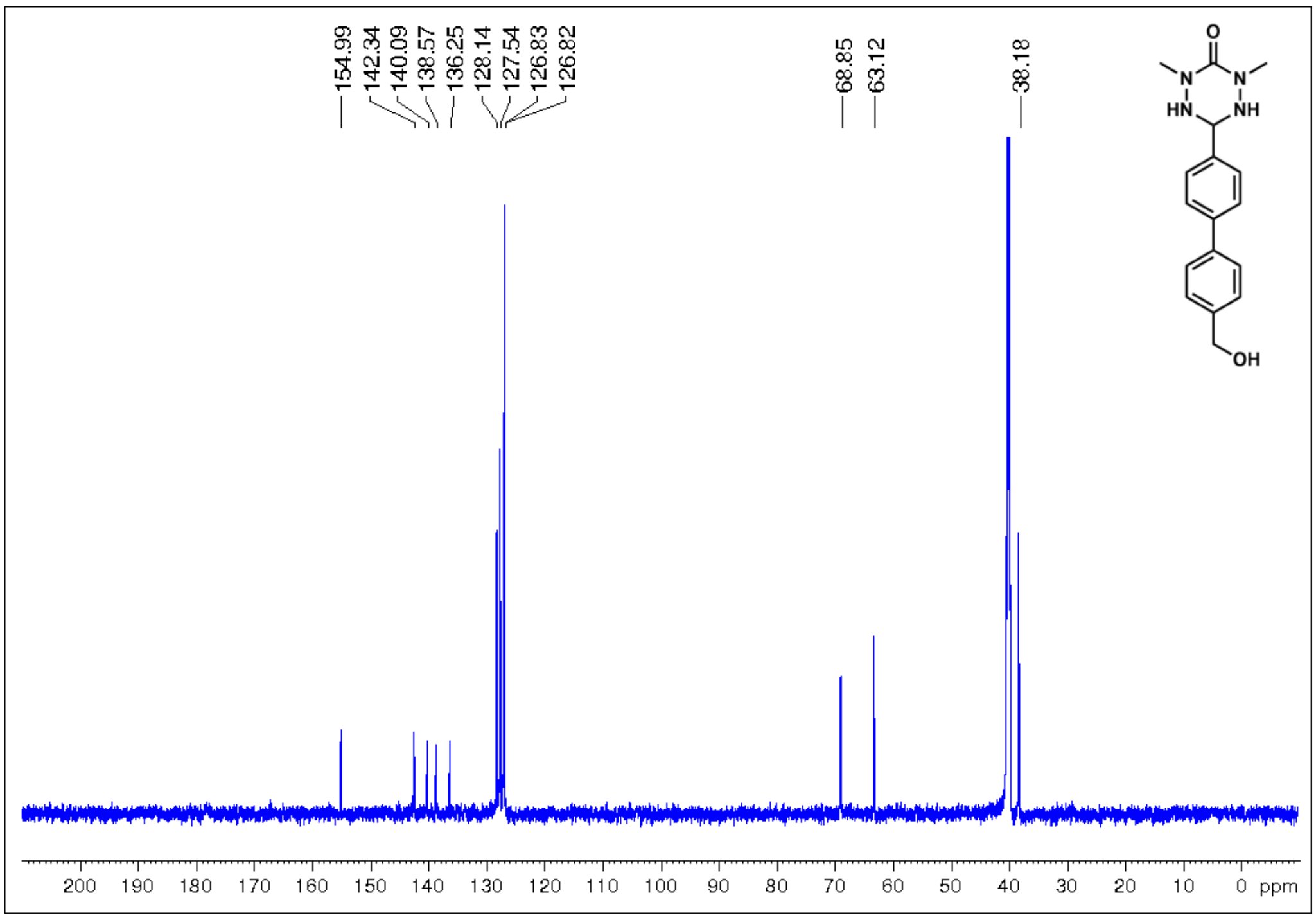

Figure S6: ^{13}C{^{1}H} NMR (150 MHz, DMSO-d_6) of **T3**.

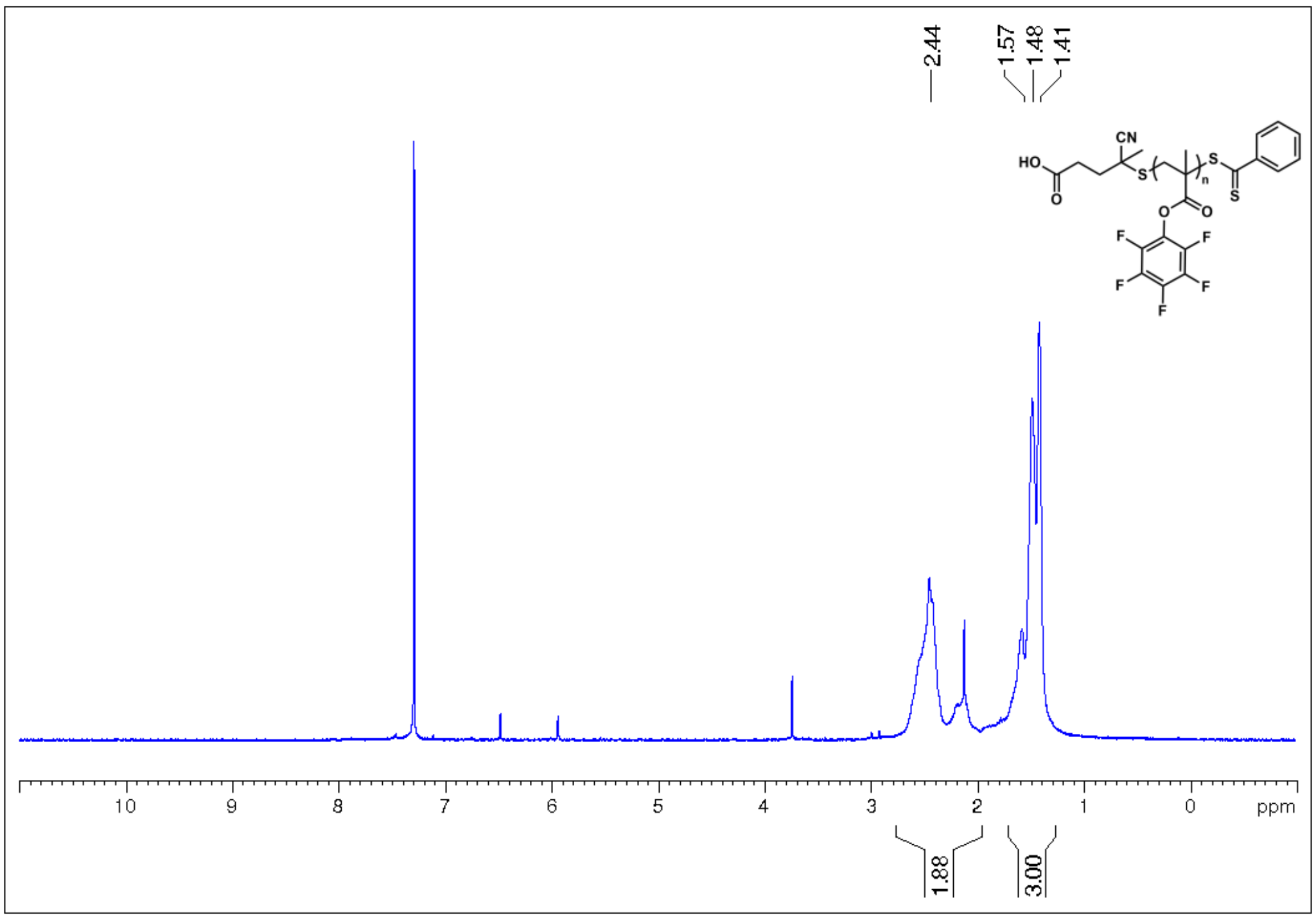

Figure S7: ^{1}H NMR (600 MHz, $CDCl_3$) of **P1**.

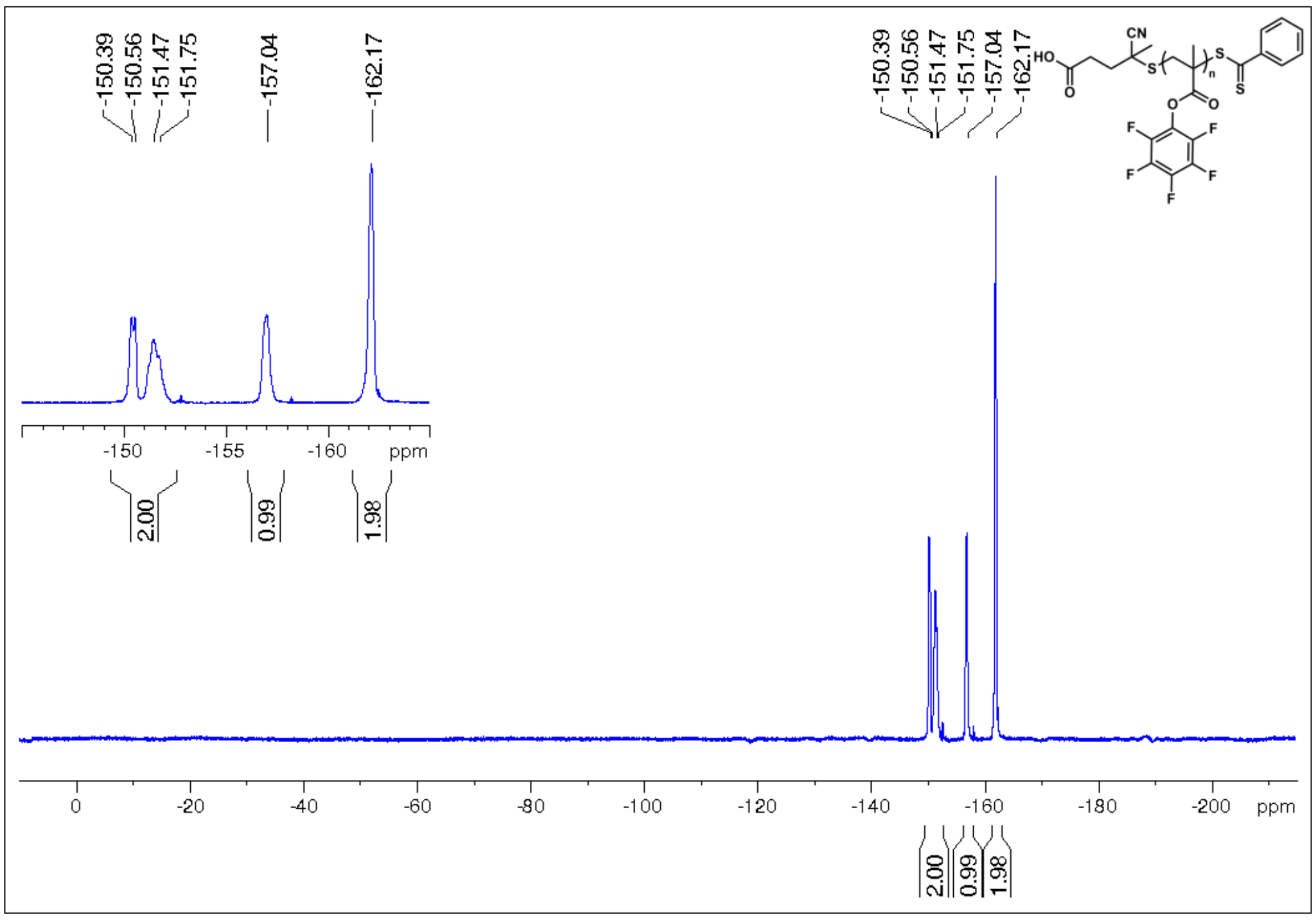

Figure S8: ^{19}F NMR (565 MHz, $CDCl_3$) of **P1**.

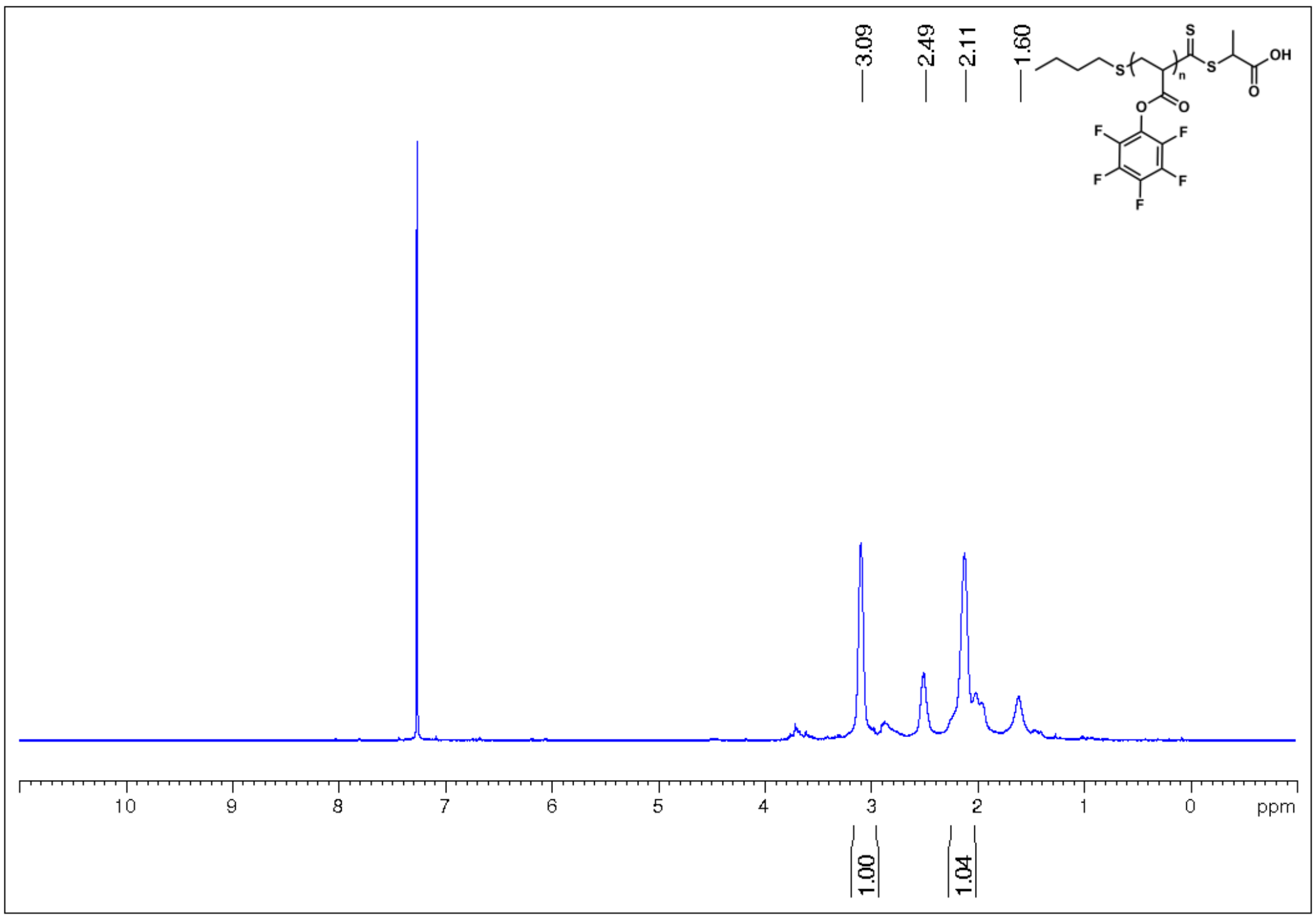

Figure S9: ^{1}H NMR (600 MHz, $CDCl_3$) of **P2**.

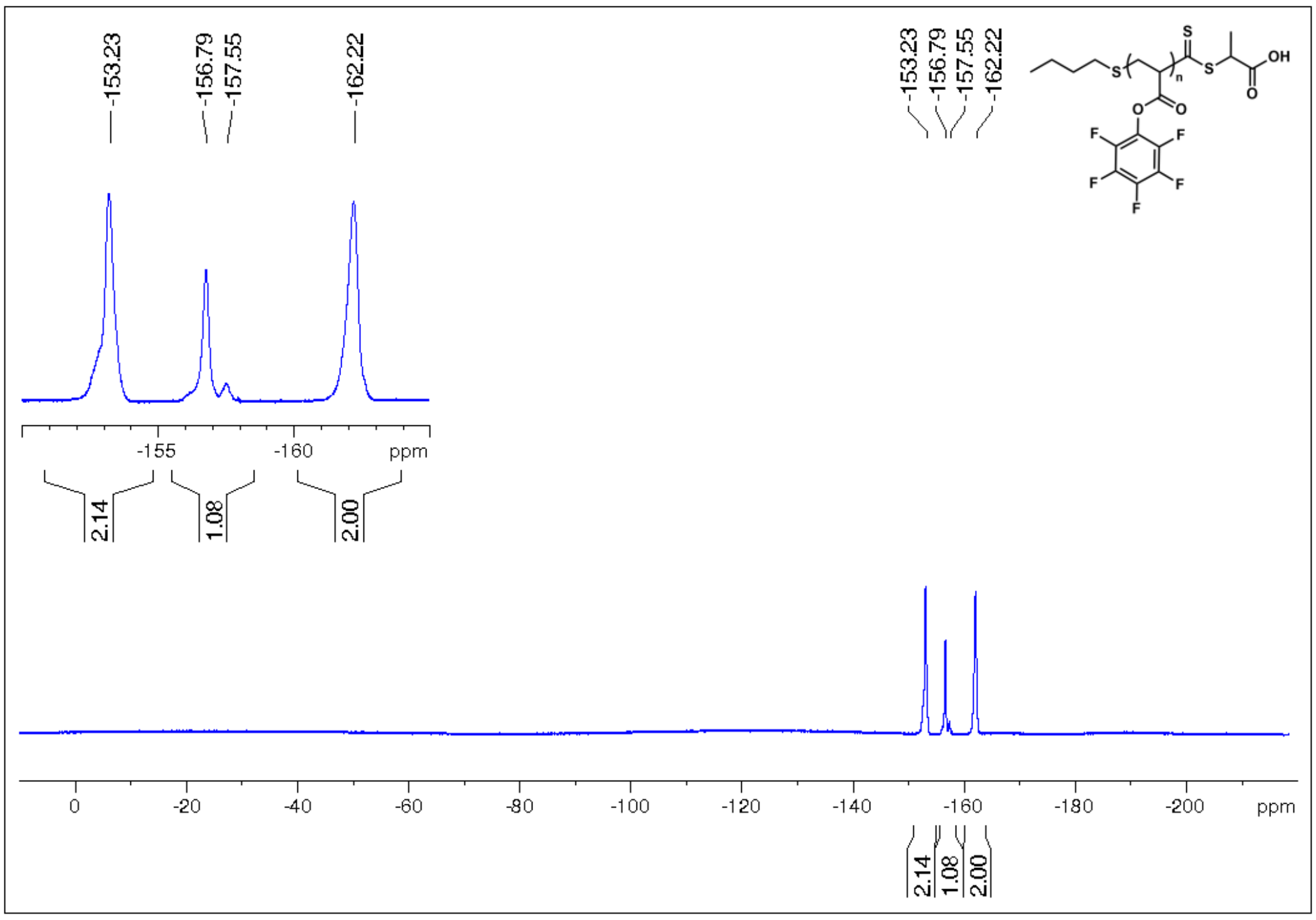

Figure S10: ^{19}F NMR (565 MHz, $CDCl_3$) of **P2**.

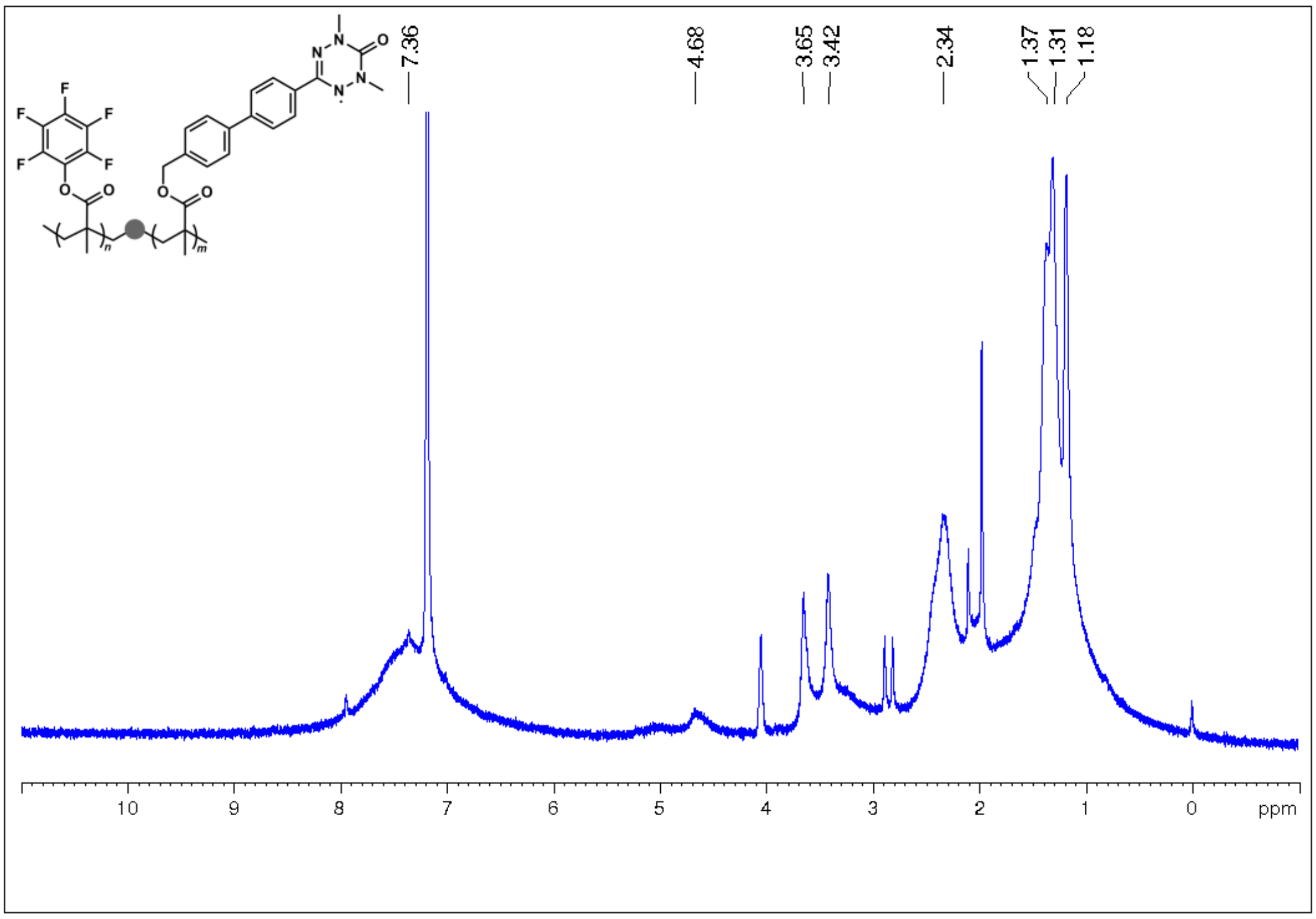

Figure S11: ^{1}H NMR (600 MHz, $CDCl_3$) of **P1-3**.

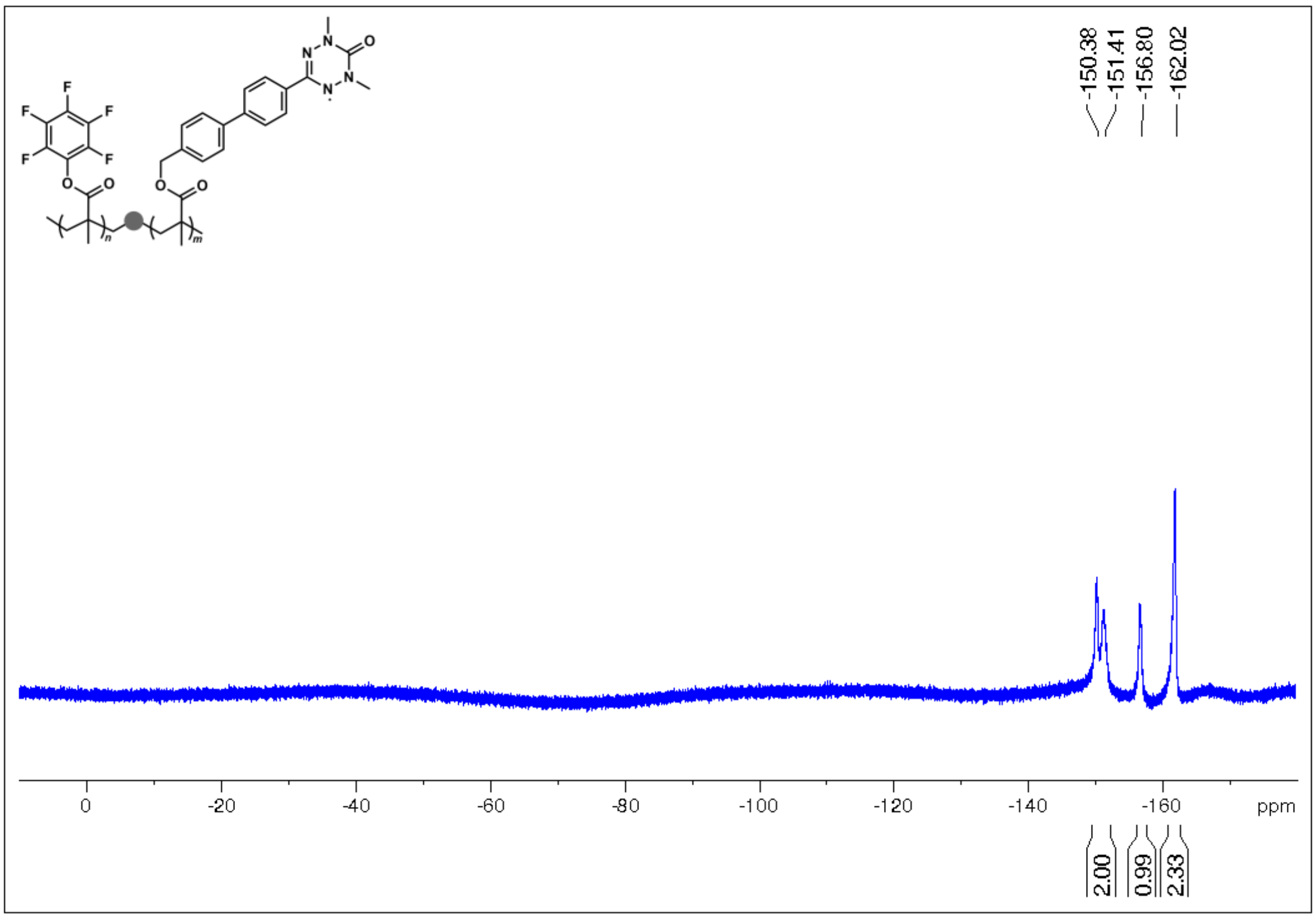

Figure S12: ^{19}F NMR (600 MHz, $CDCl_3$) of **P1-3**.

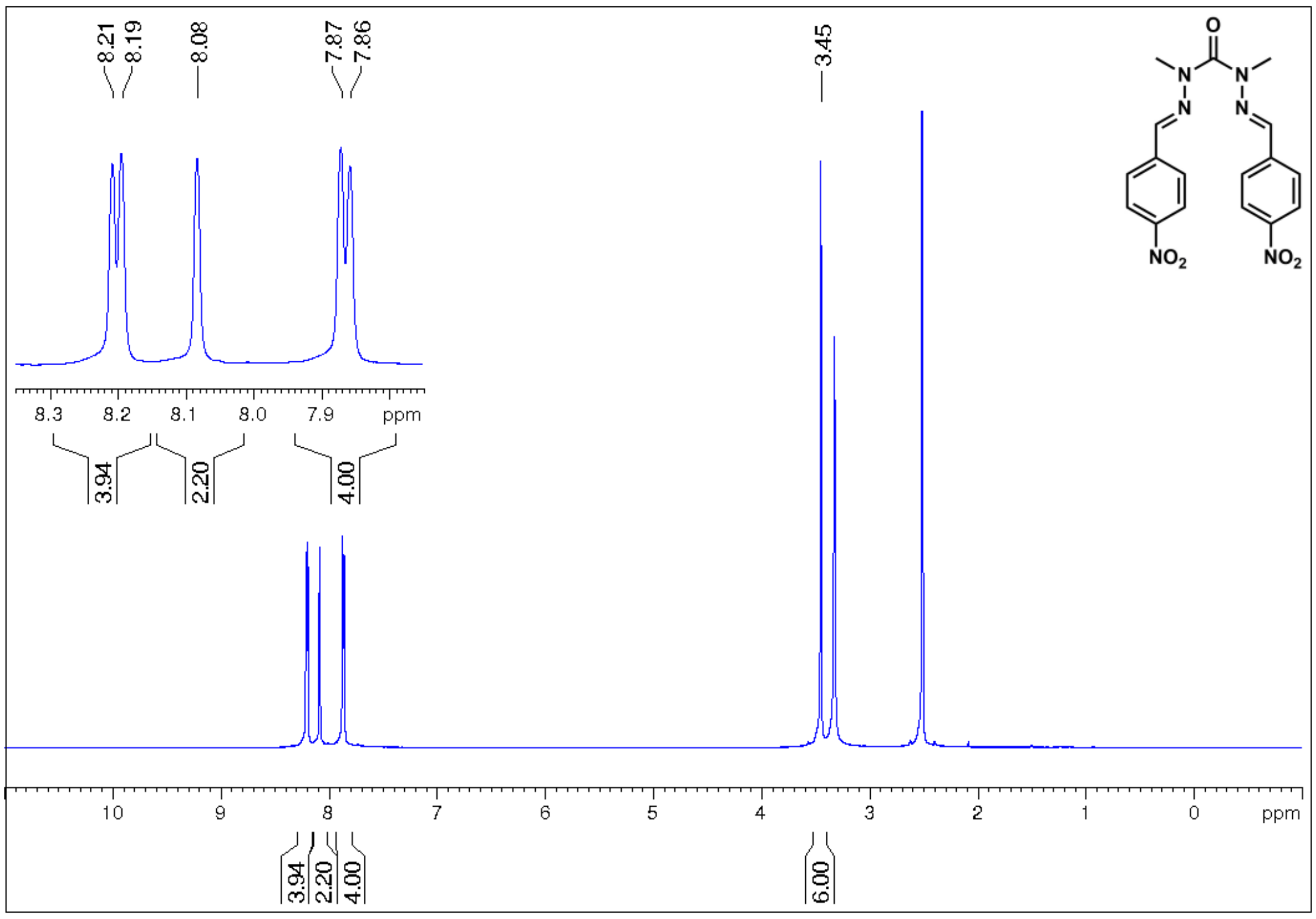

Figure S13: ^{1}H NMR (600 MHz, DMSO-d_6) of carbo-di(*N'*-4-nitrobenzylidene-*N*-methylhydrazide).

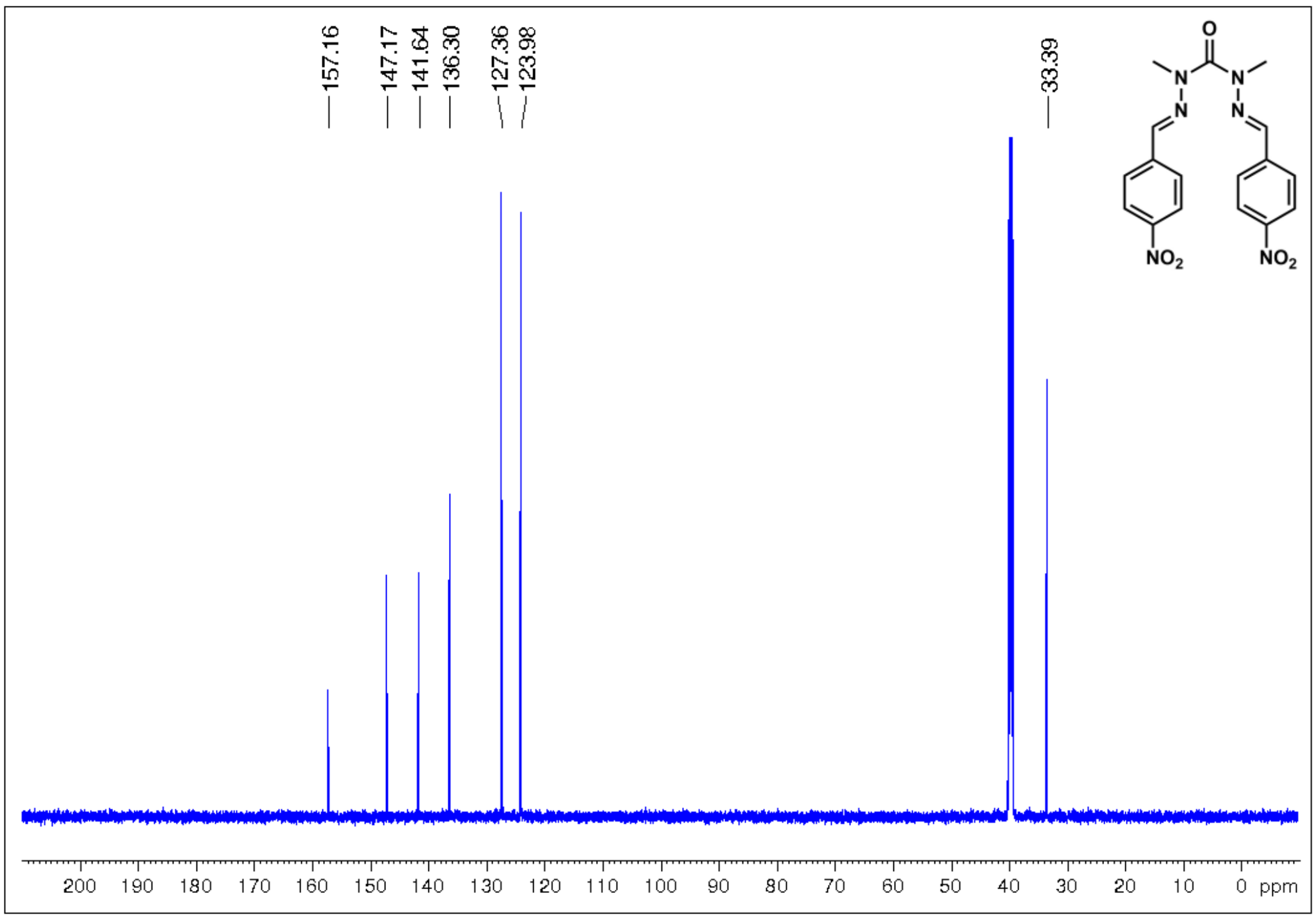

Figure S14: ^{13}C{^{1}H} NMR (150 MHz, DMSO-d_6) of carbo-di(*N'*-4-nitrobenzylidene-*N*-methylhydrazide).

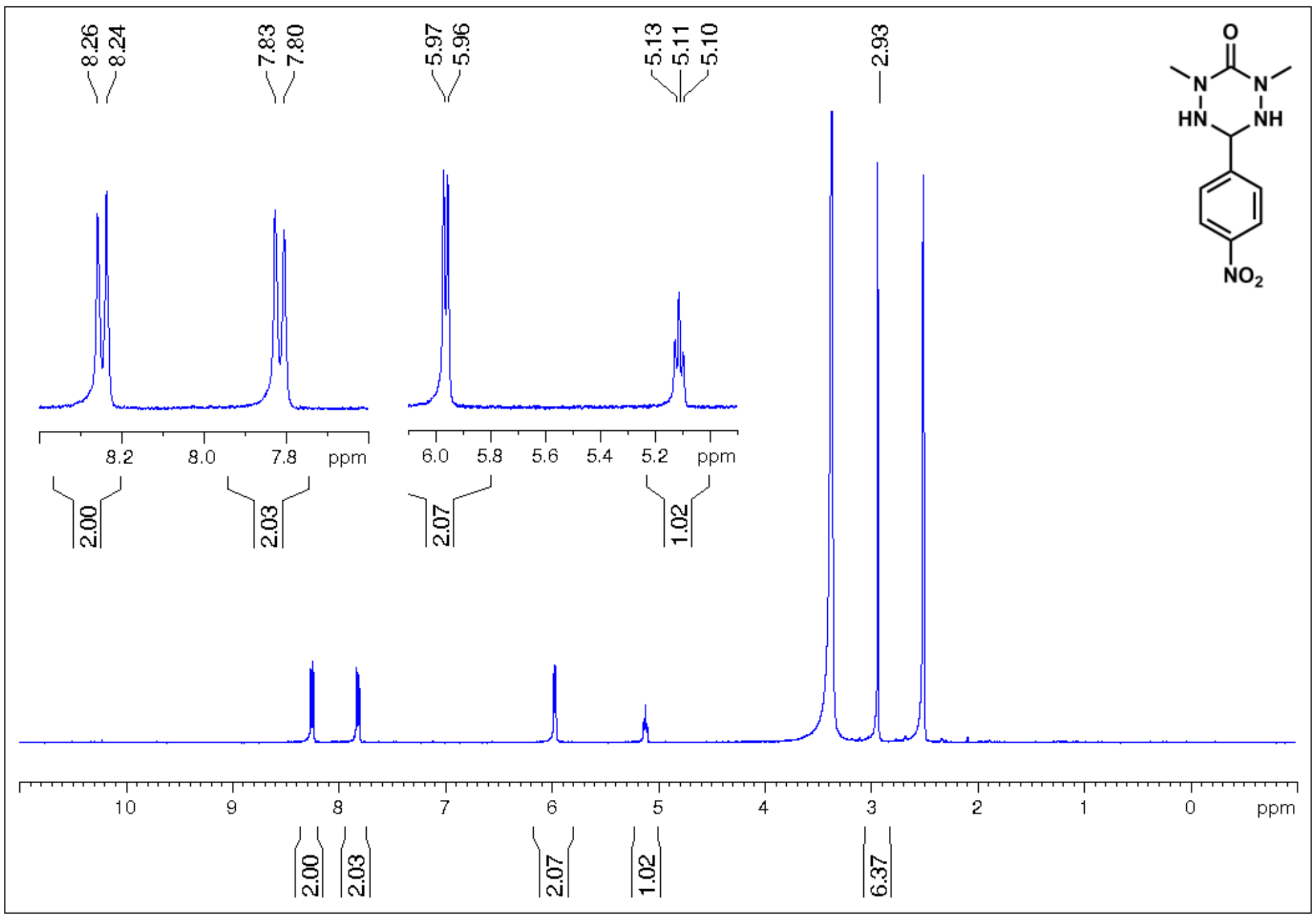

Figure S15: ^{1}H NMR (600 MHz, DMSO-d_6) of 2,4-dimethyl-6-(4-nitrophenyl)-1,2,4,5-tetrazinan-3-one.

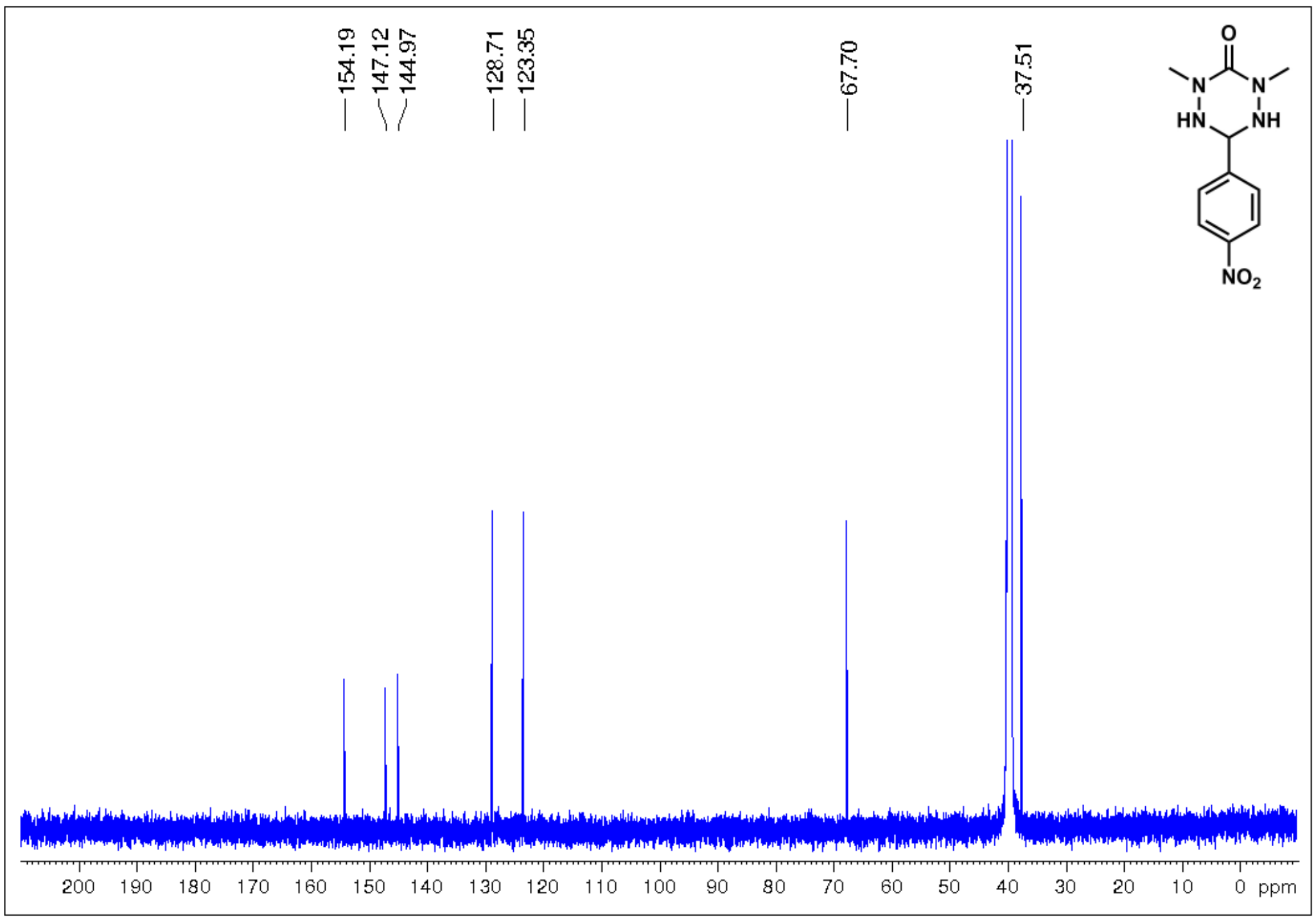

Figure S16: $^{13}C\{^{1}H\}$ NMR (150 MHz, DMSO-d_6) of 2,4-dimethyl-6-(4-nitrophenyl)-1,2,4,5-tetrazinan-3-one.

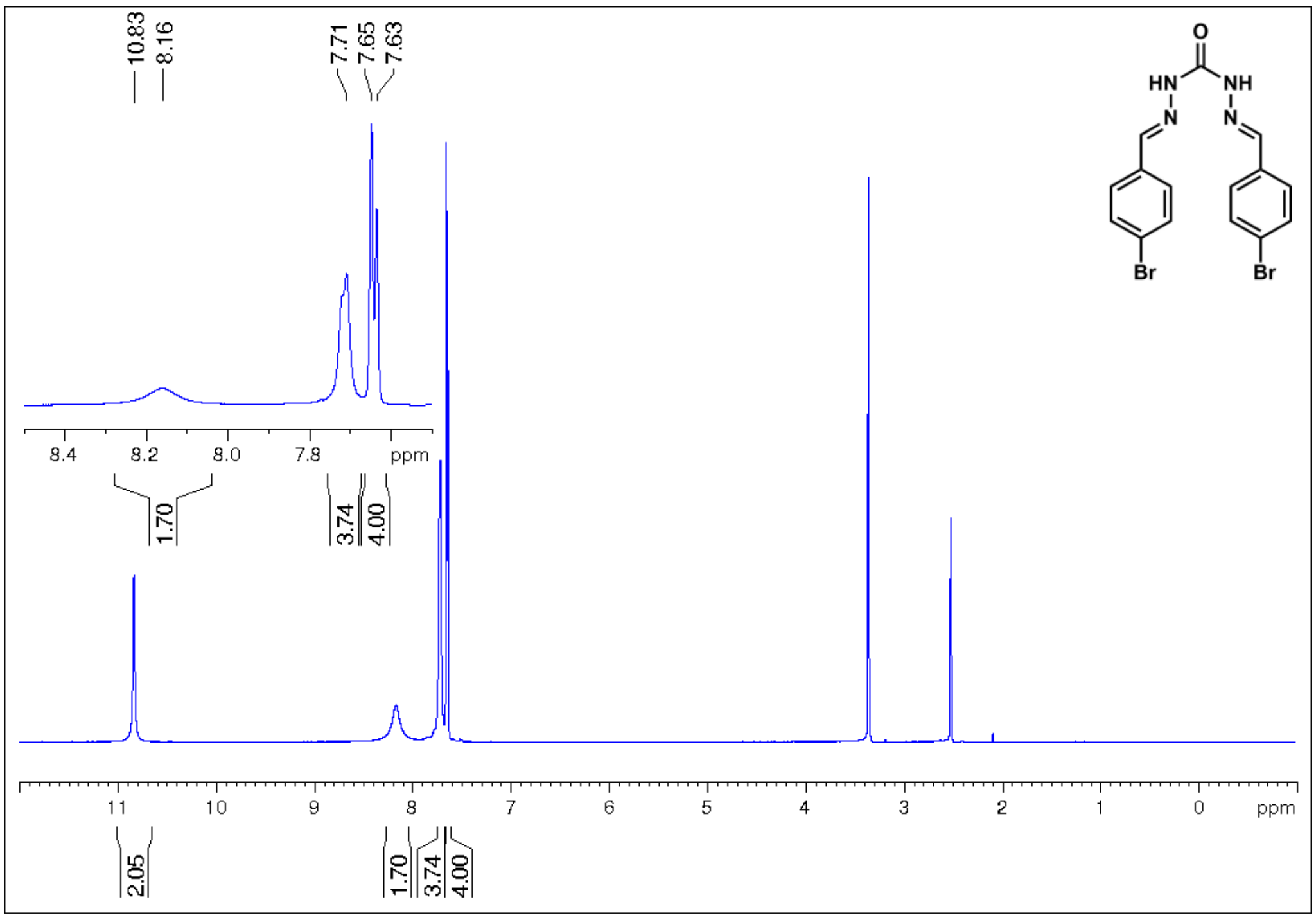

Figure S17: ^{1}H NMR (600 MHz, DMSO-d_6) of carbo-di-(*N'*-4-bromophenylhydrazide).

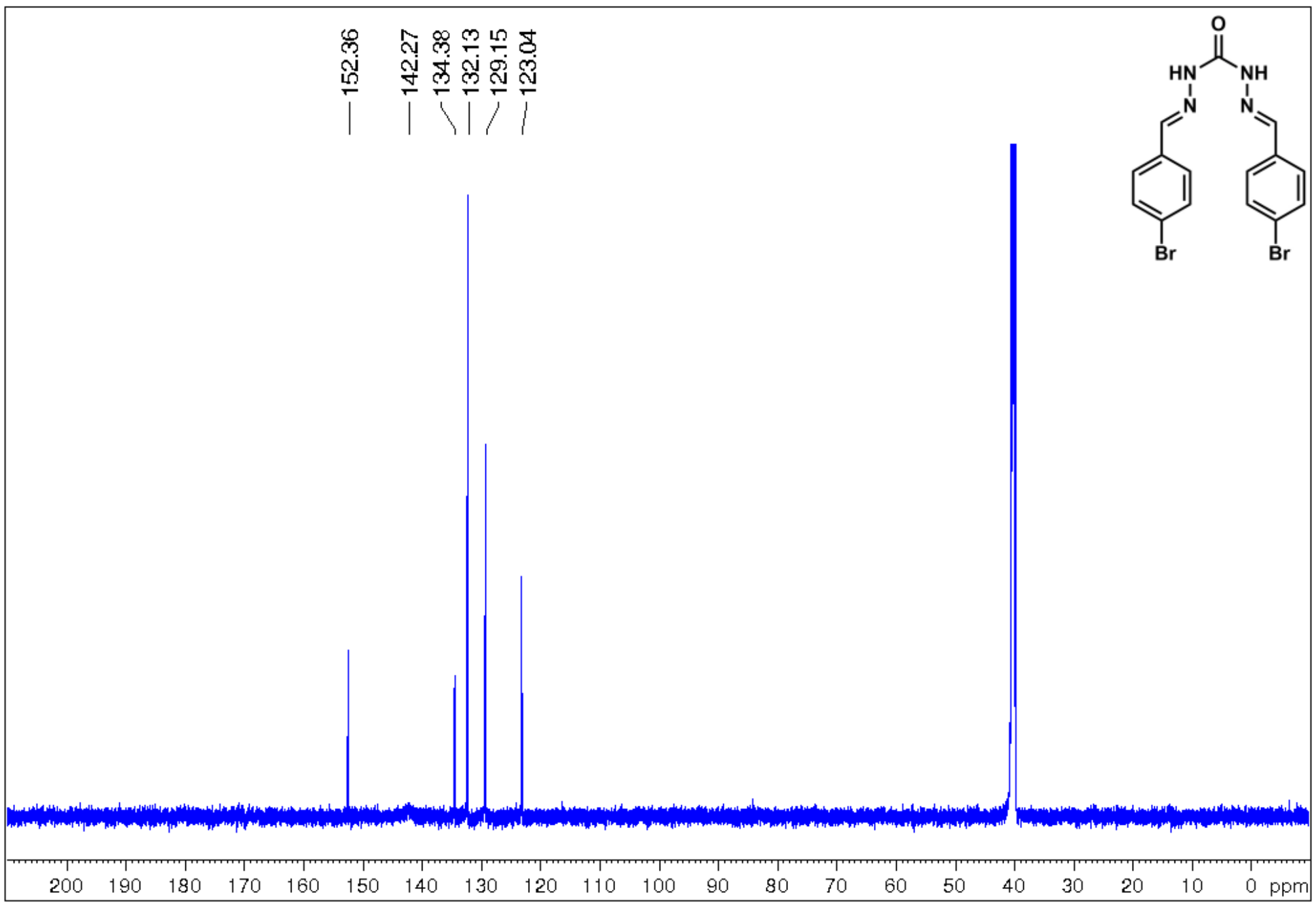

Figure S18: ^{13}C{^{1}H} NMR (150 MHz, DMSO-d_6) of carbo-di-(N'-4-bromophenylhydrazide).

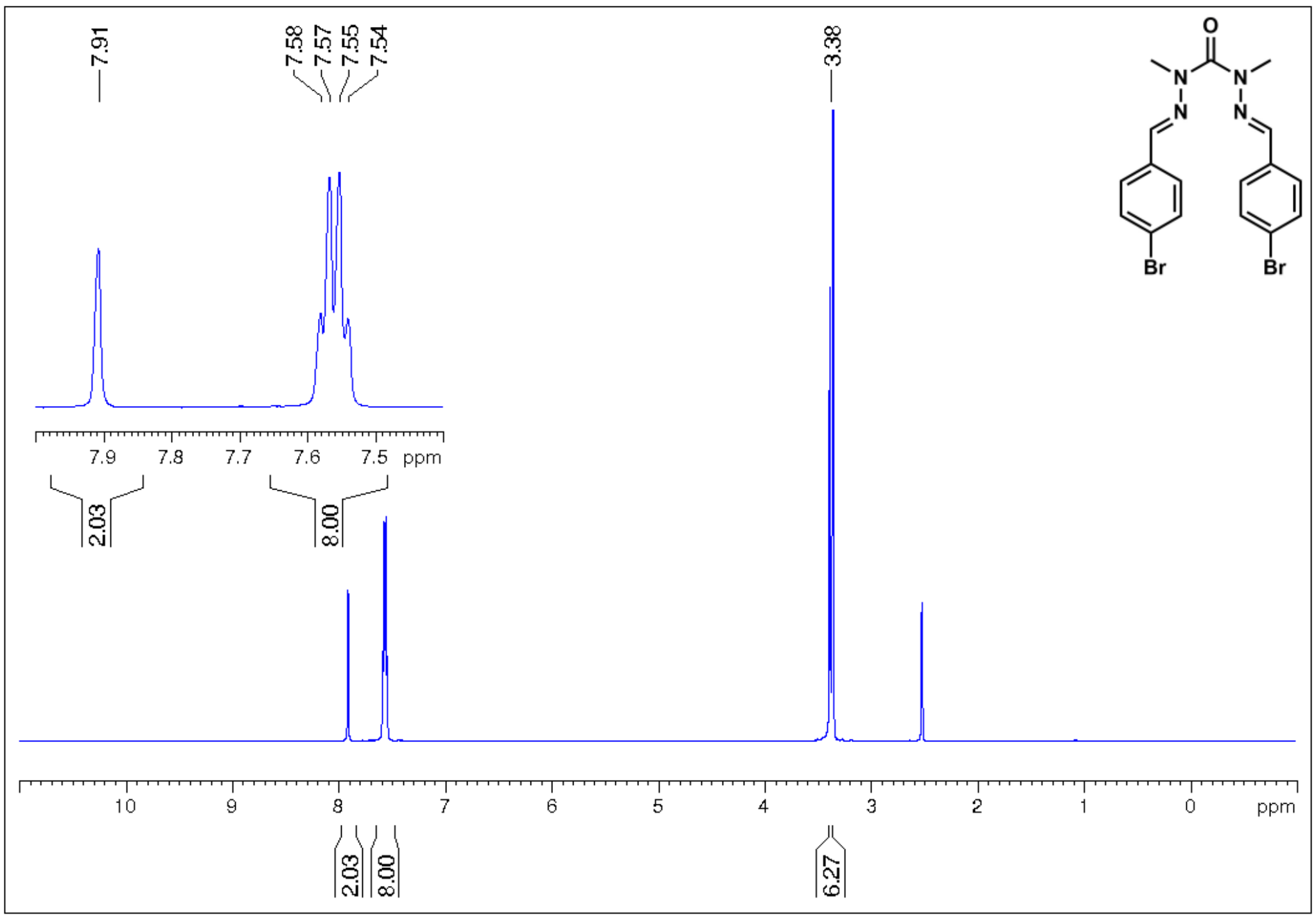

Figure S19: ^{1}H NMR (600 MHz, DMSO-d_6) of carbo-di(*N'*-4-bromophenylidene-*N*-methylhydrazide).

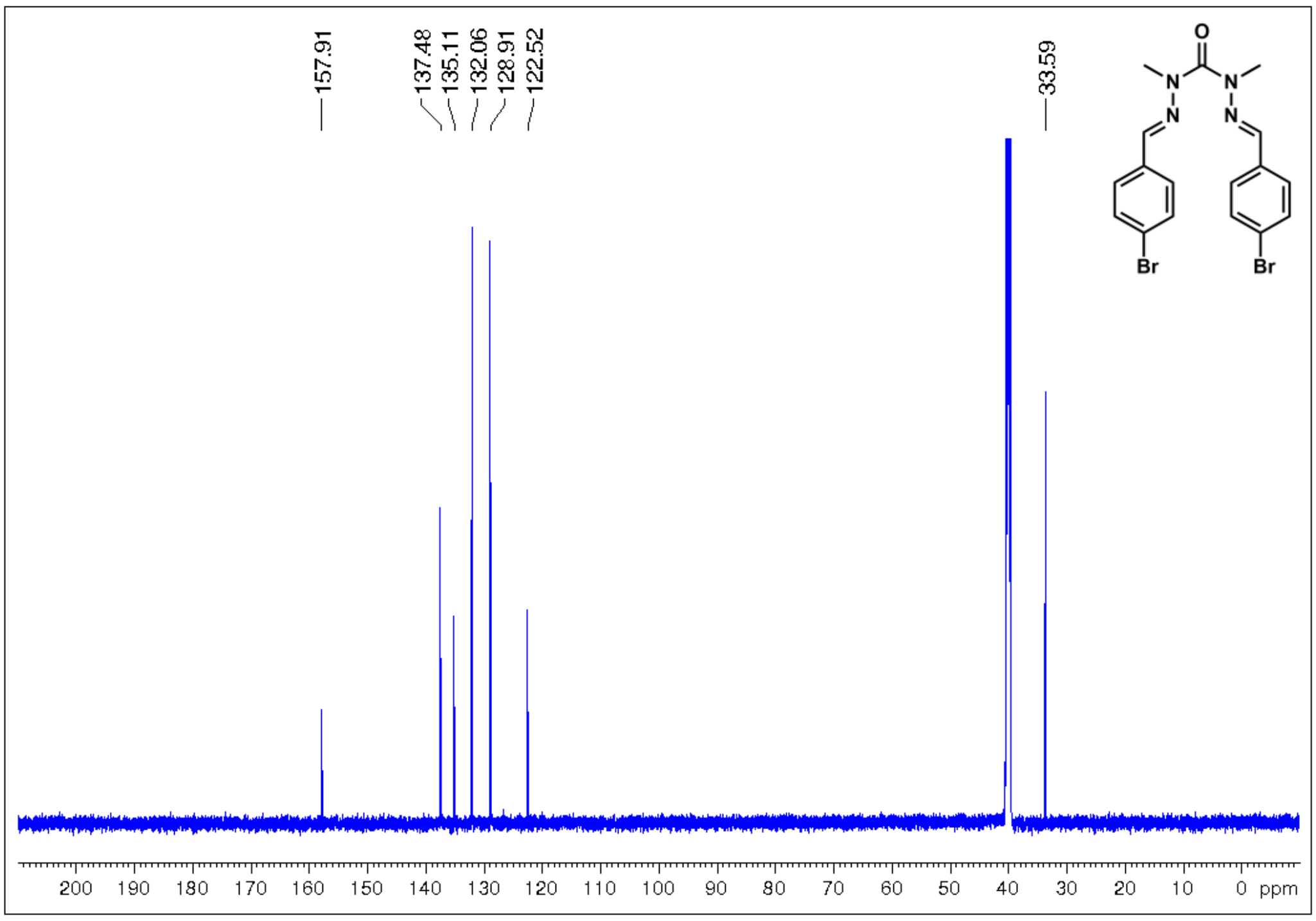

Figure S20: ^{13}C{^{1}H} NMR (150 MHz, DMSO-d_6) of carbo-di(*N'*-4-bromophenylidene-*N*-methylhydrazide).

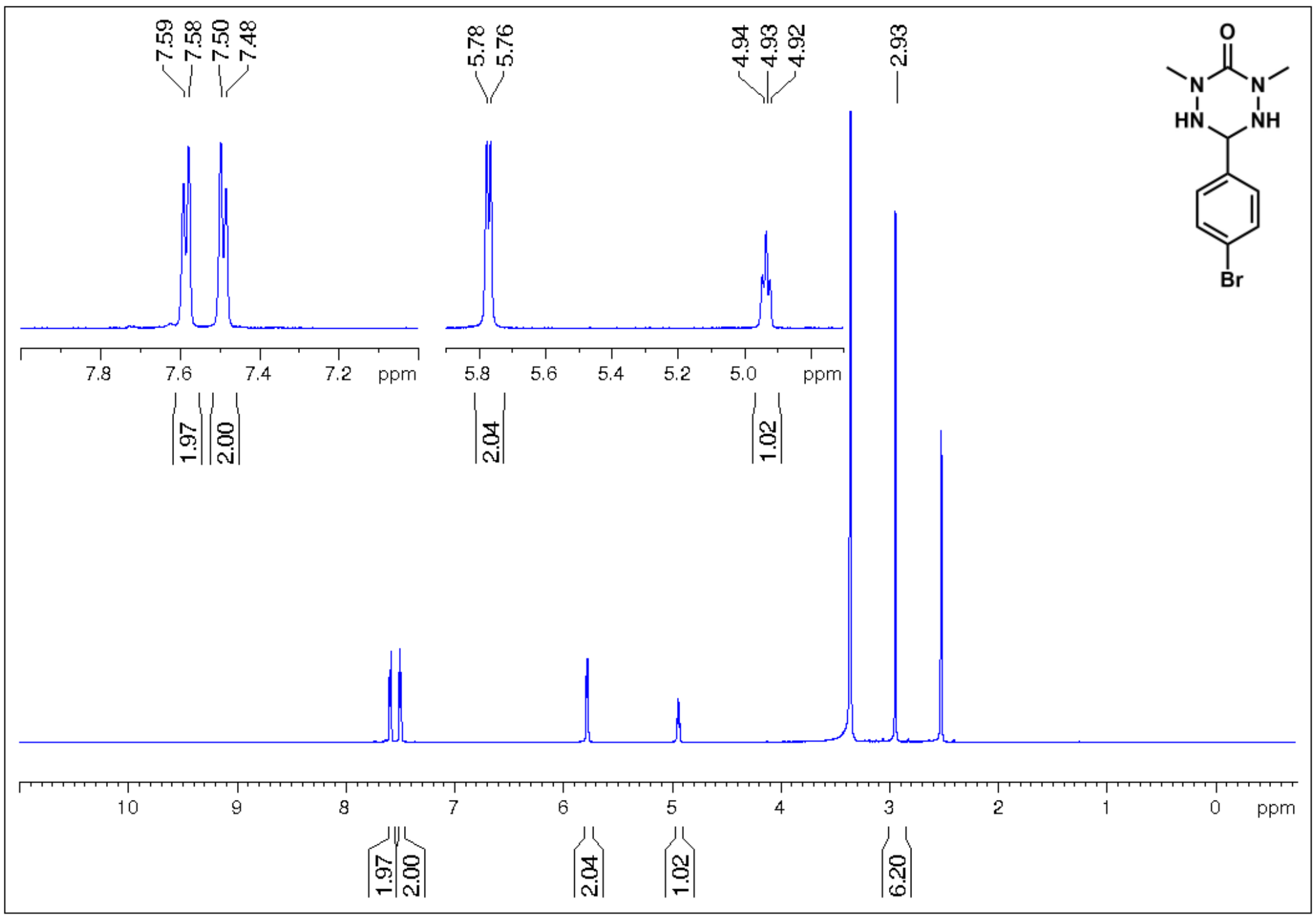

Figure S21: ^{1}H NMR (600 MHz, DMSO-d_6) of 6-(4-bromophenyl)-2,4-dimethyl-1,2,4,5-tetrazinan-3-one.

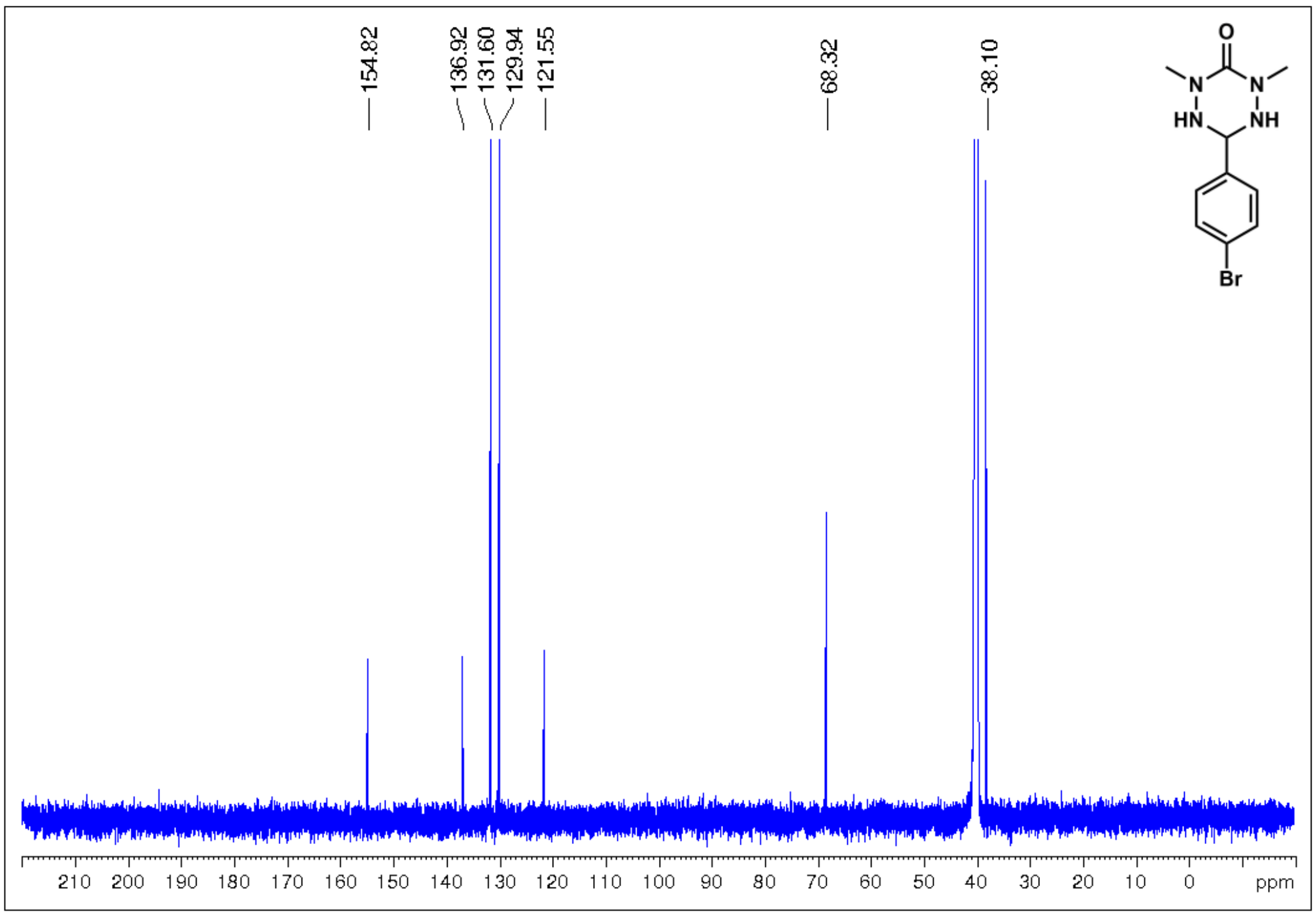

Figure S22: $^{13}C\{^{1}H\}$ NMR (150 MHz, DMSO-d_6) of 6-(4-bromophenyl)-2,4-dimethyl-1,2,4,5-tetrazinan-3-one.

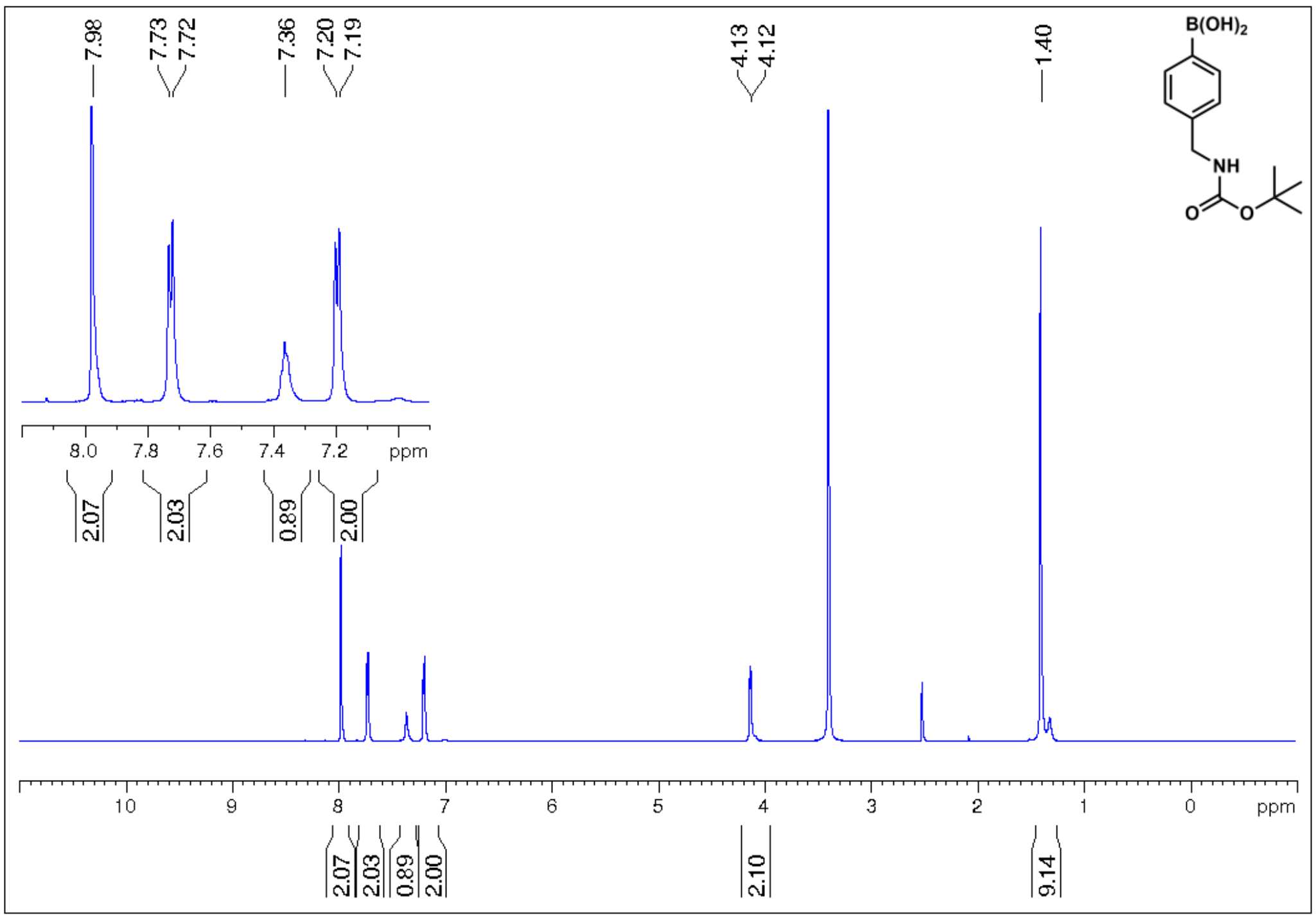

Figure S23: ^{1}H NMR (600 MHz, DMSO-d$_6$) of (4-(pivalamidomethyl)phenyl)boronic acid.

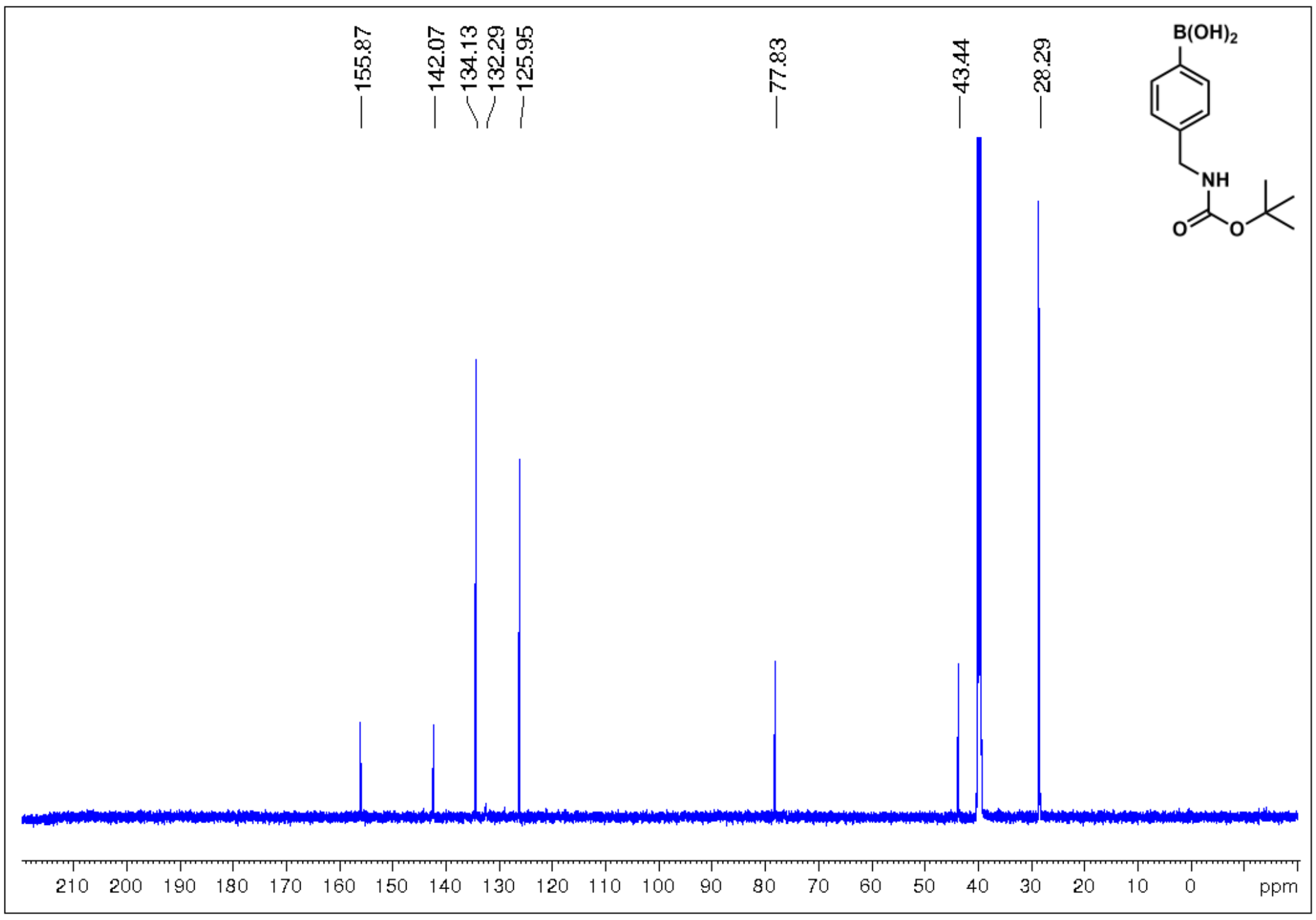

Figure S24: ^{13}C{^{1}H} NMR (150 MHz, DMSO-d$_6$) of (4-(pivalamidomethyl)phenyl)boronic acid.

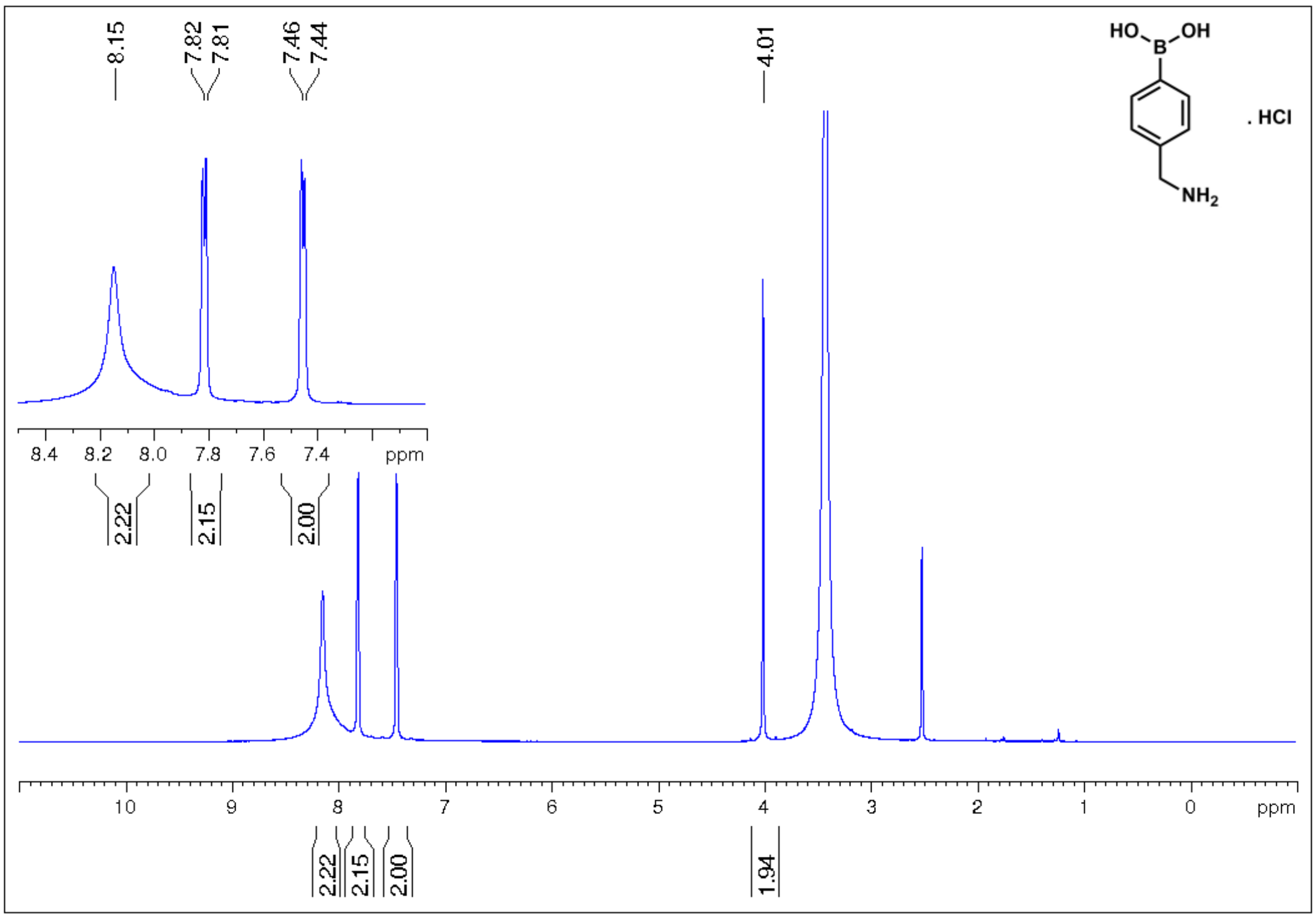

Figure S25: ^{1}H NMR (600 MHz, DMSO-d_6) of (4-(Aminomethyl)phenyl)boronic acid hydrochloride.

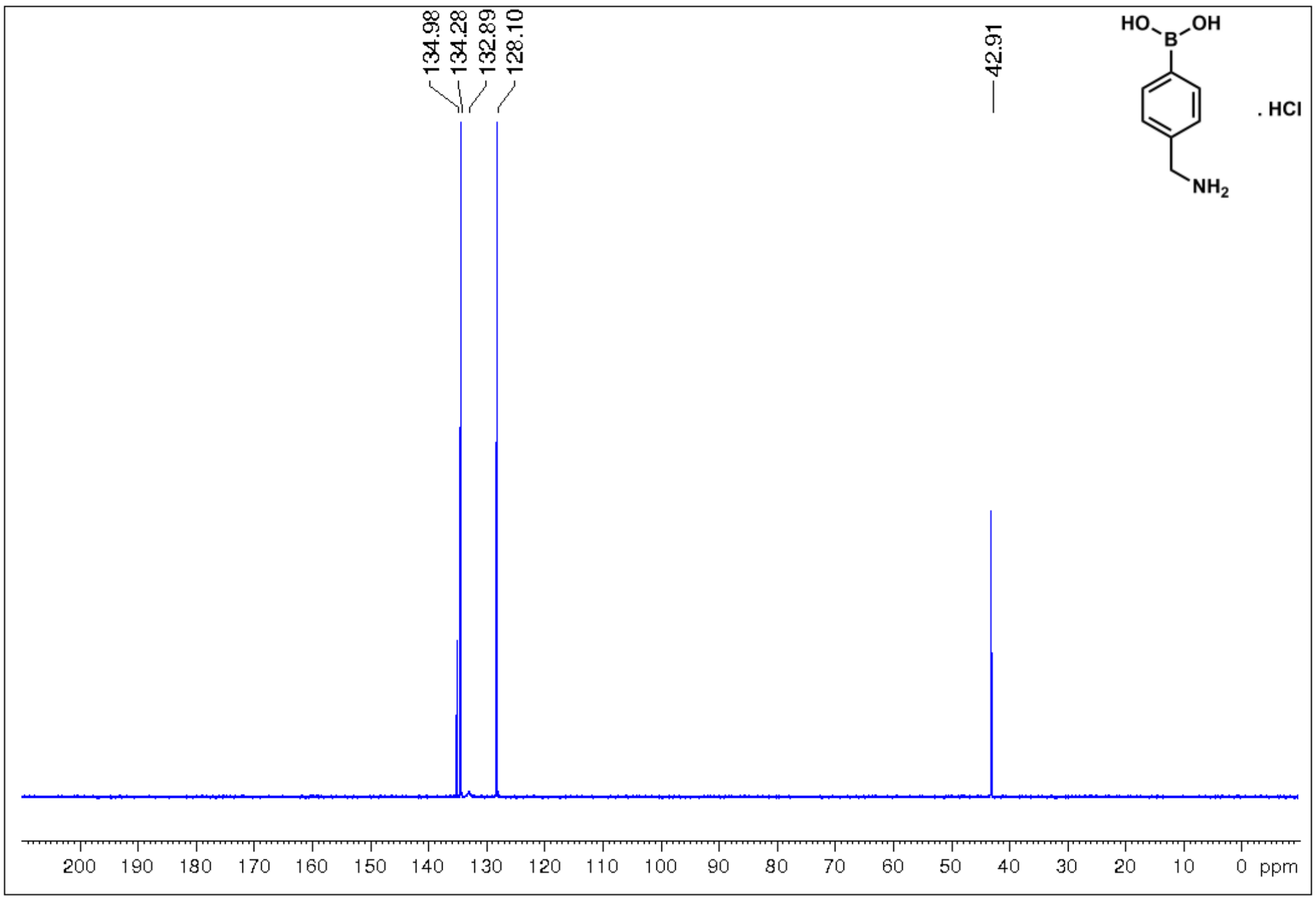

Figure S26: $^{13}C\{^{1}H\}$ NMR (150 MHz, D_2O) of (4-(Aminomethyl)phenyl)boronic acid hydrochloride.

III Electron paramagnetic resonance spectroscopy

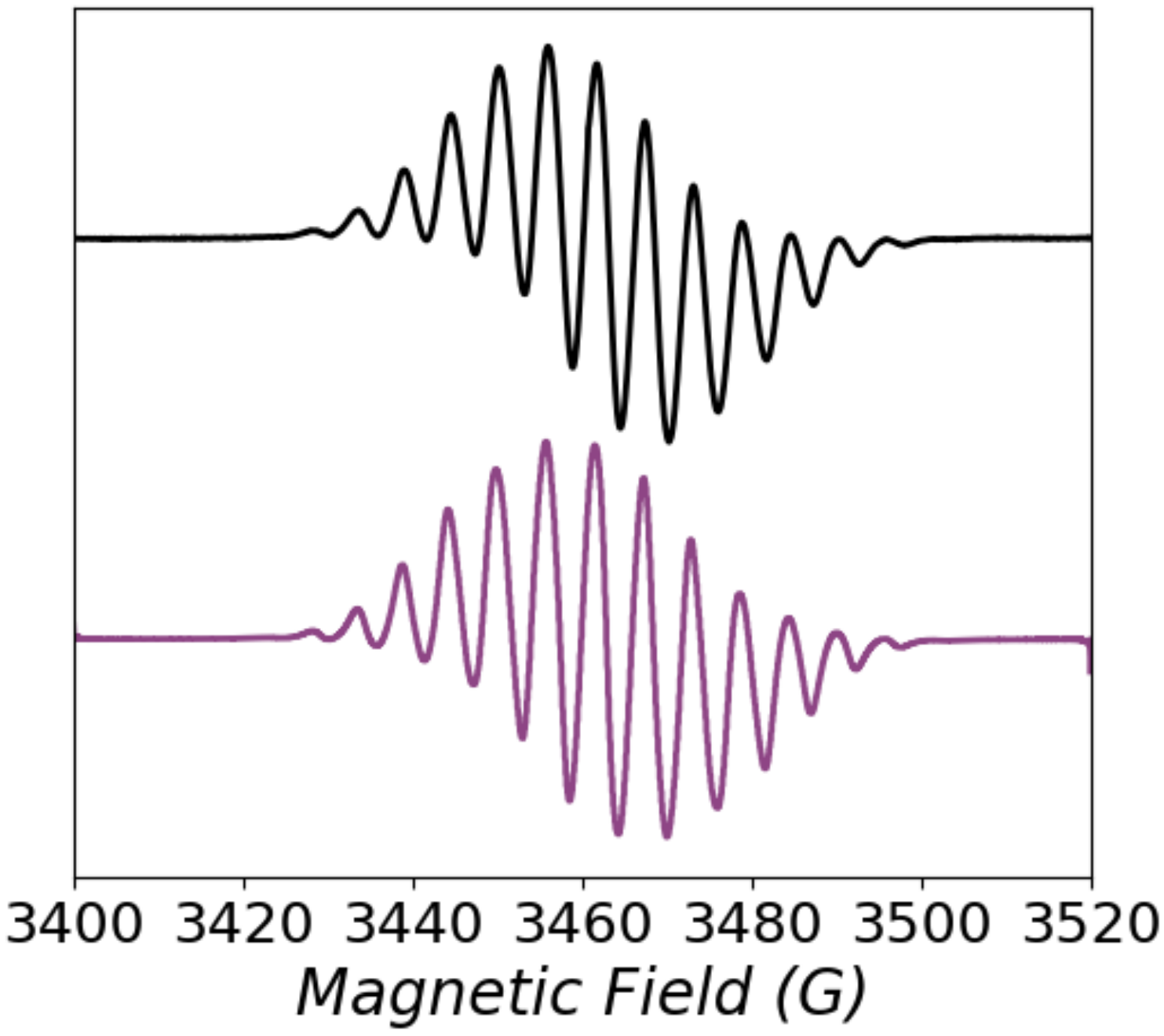

Figure S27. The first derivative X-band (CW) EPR spectrum (n = 9.69 GHz) of **1** in degassed toluene at 298 K (black) together with simulation (purple) using EasySpin[7] garlic package in MATLAB. The spectrum was recorded with a field modulation of 2.1 G and microwave power of 10 mW.

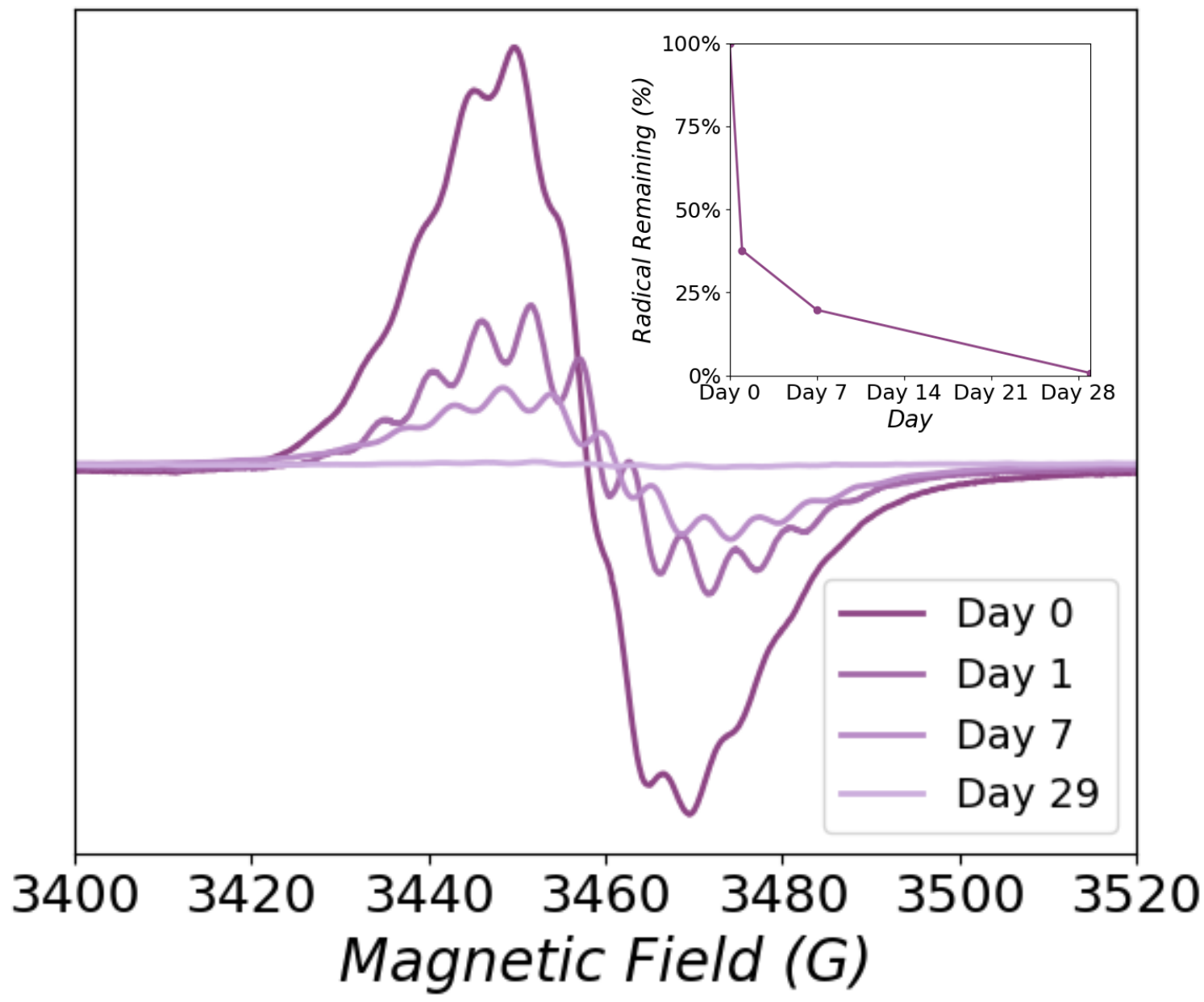

Figure S28. The first derivative X-band (CW) (n = 9.69 GHz) EPR spectrum of **1** in degassed toluene at 298 K. The spectrum was recorded with a field modulation of 2.1 G and microwave power of 10 mW. Further spectra are collected over 1 month using similar experimental. Radical **1** has been stored as a solid under ambient conditions, to ensure disporoptionation is minimised. **1** is and added to degassed toluene, at the same concentration, for measurements to be performed. Inset contains decomposition analysis the peak-to-peak amplitude as a percentage of the original peak-to-peak amplitude of the signal.

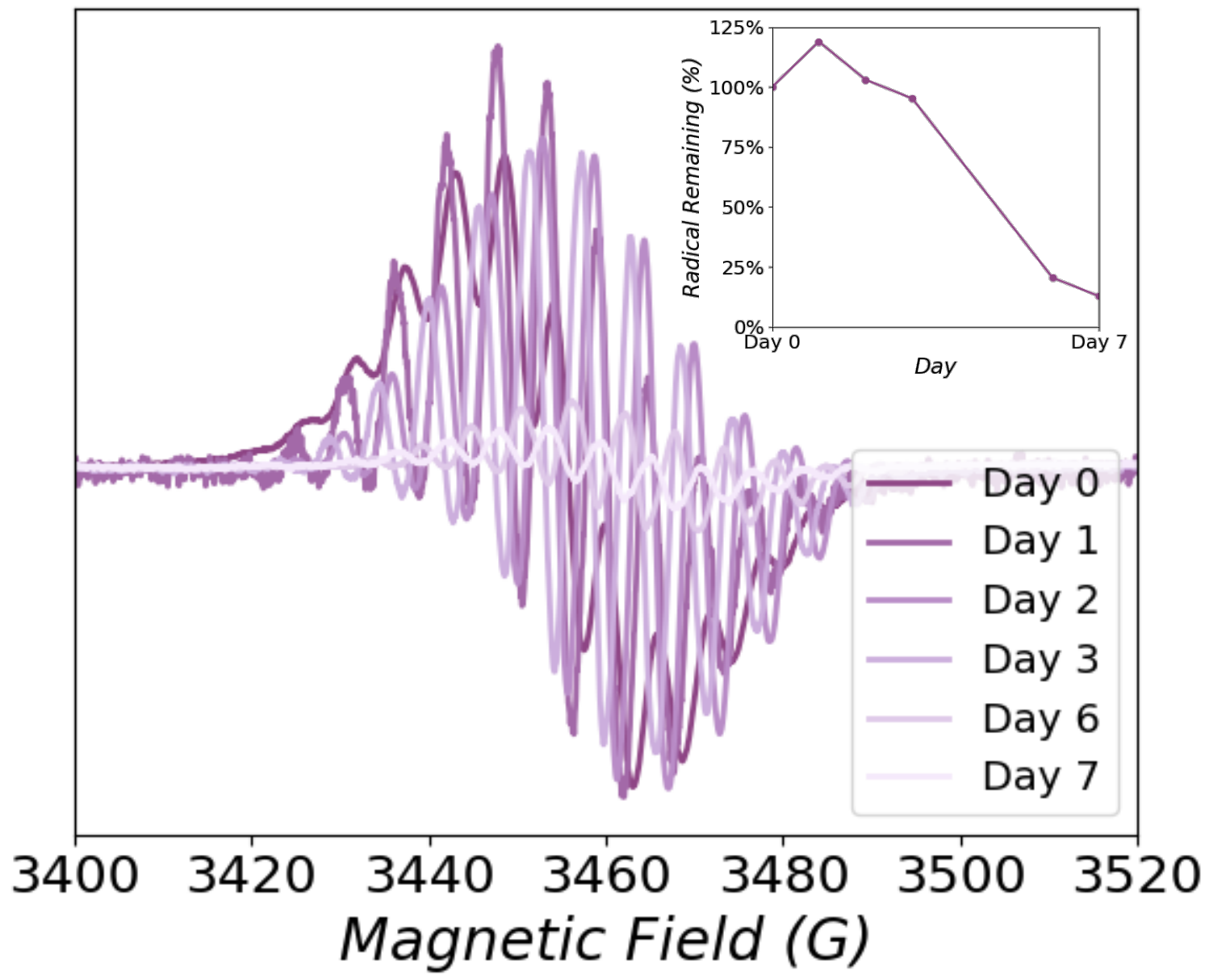

Figure S29. The first derivative X-band (CW) (*n* = 9.69 GHz) EPR spectrum of **2** in tetrahydrofuran at 298 K. The spectrum was recorded with a field modulation of 2.1 G and microwave power of 10 mW. Further spectra are collected over 1 month using similar experimental. Radical **2** has been stored in solution, inside a sealed capillary. Inset contains decomposition analysis the peak-to-peak amplitude as a percentage of the original peak-to-peak amplitude of the signal.

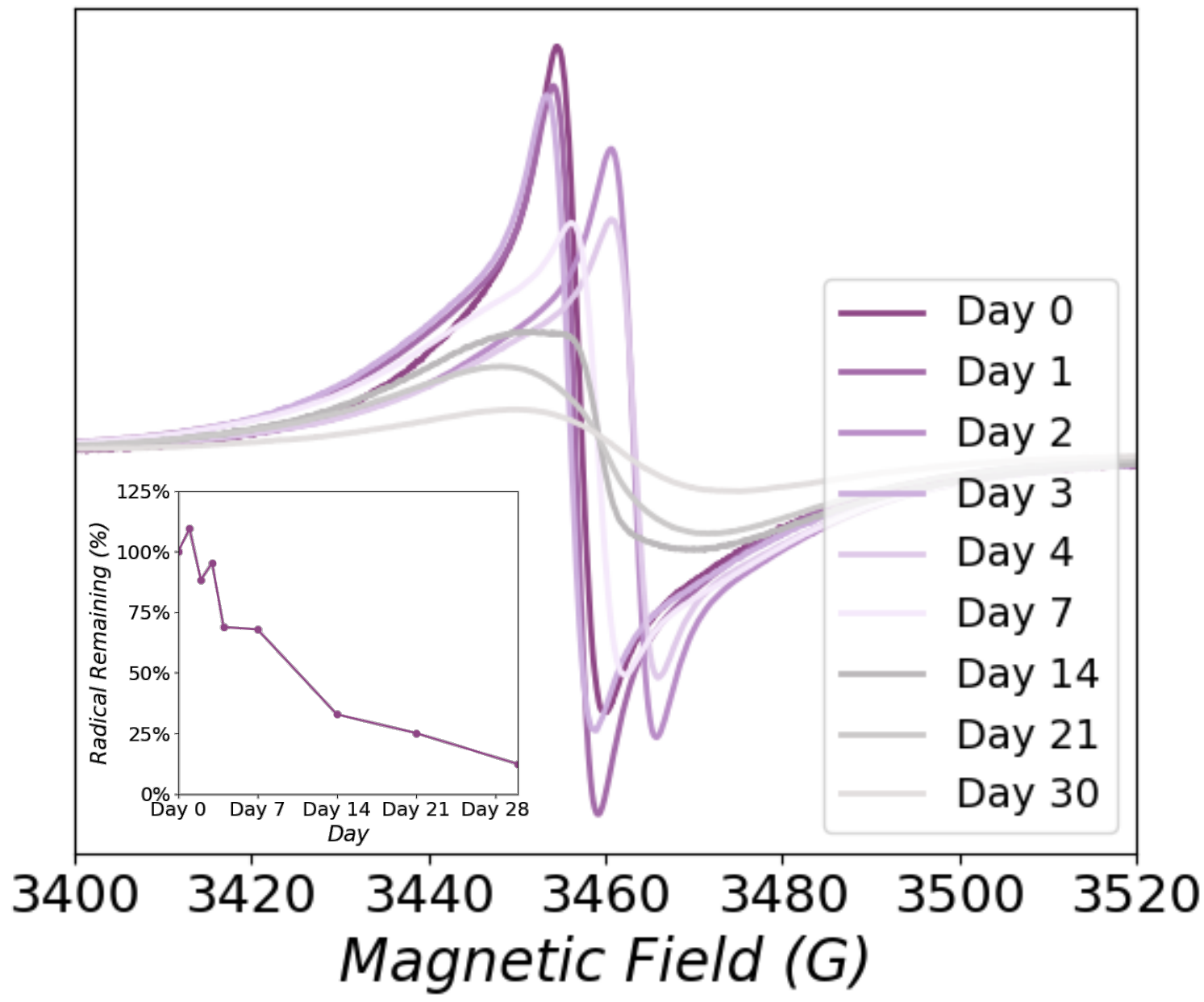

Figure S30. The first derivative X-band (CW) (*n* = 9.69 GHz) EPR spectrum of solid **2** within a glass capillary at 298 K. The spectrum was recorded with a field modulation of 2.1 G and microwave power of 10 mW. Radical **2** has been stored as a solid, inside a sealed capillary. Inset contains decomposition analysis the peak-to-peak amplitude as a percentage of the original peak-to-peak amplitude of the signal.

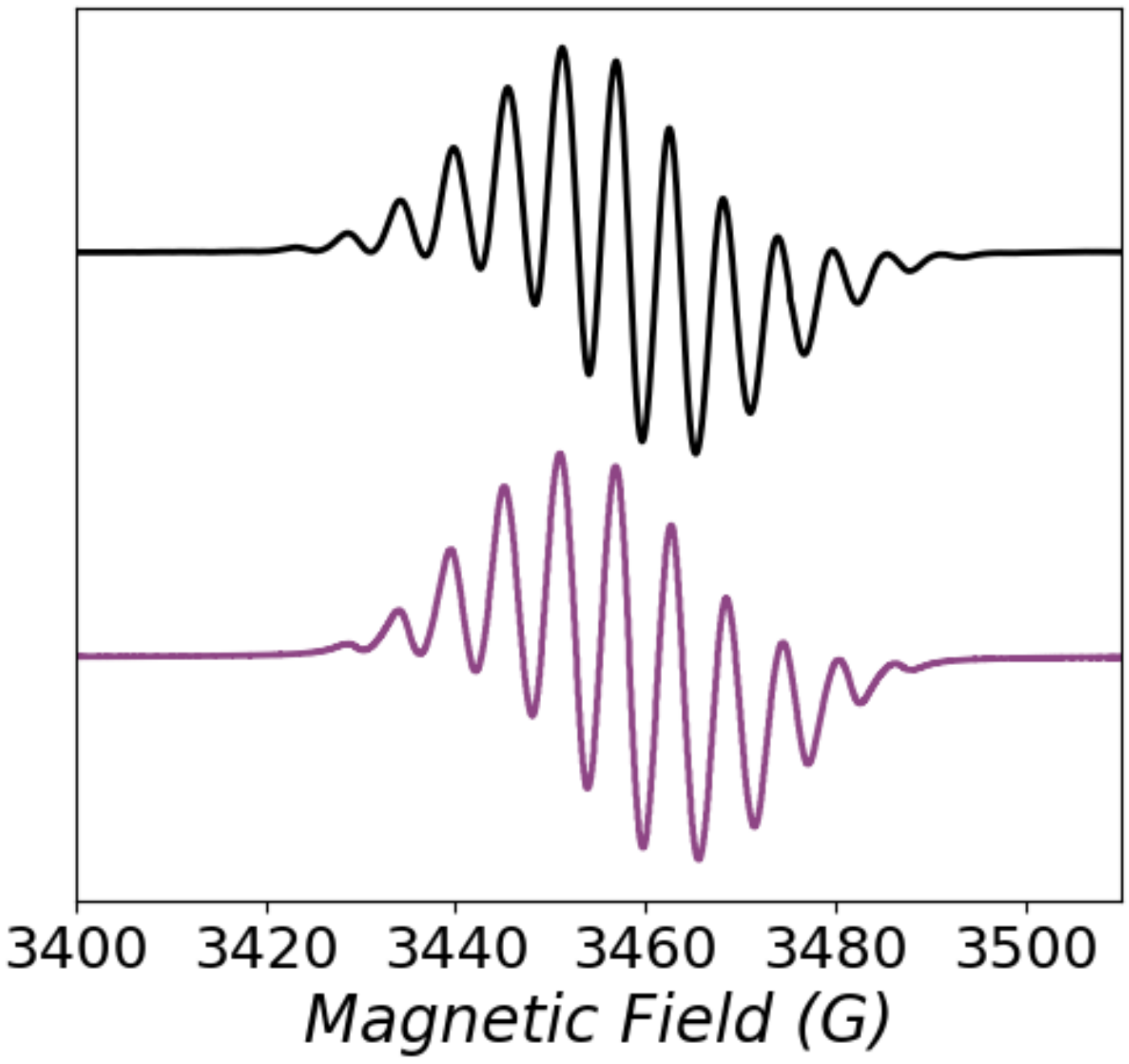

Figure S31. The first derivative X-band (CW) EPR spectrum (n = 9.69 GHz) of **2** in degassed chloroform at 298 K (upper) together with simulation (lower) using EasySpin[7] garlic package in MATLAB. The spectrum was recorded using a field modulation of 2.1 G and microwave power of 6 mW.

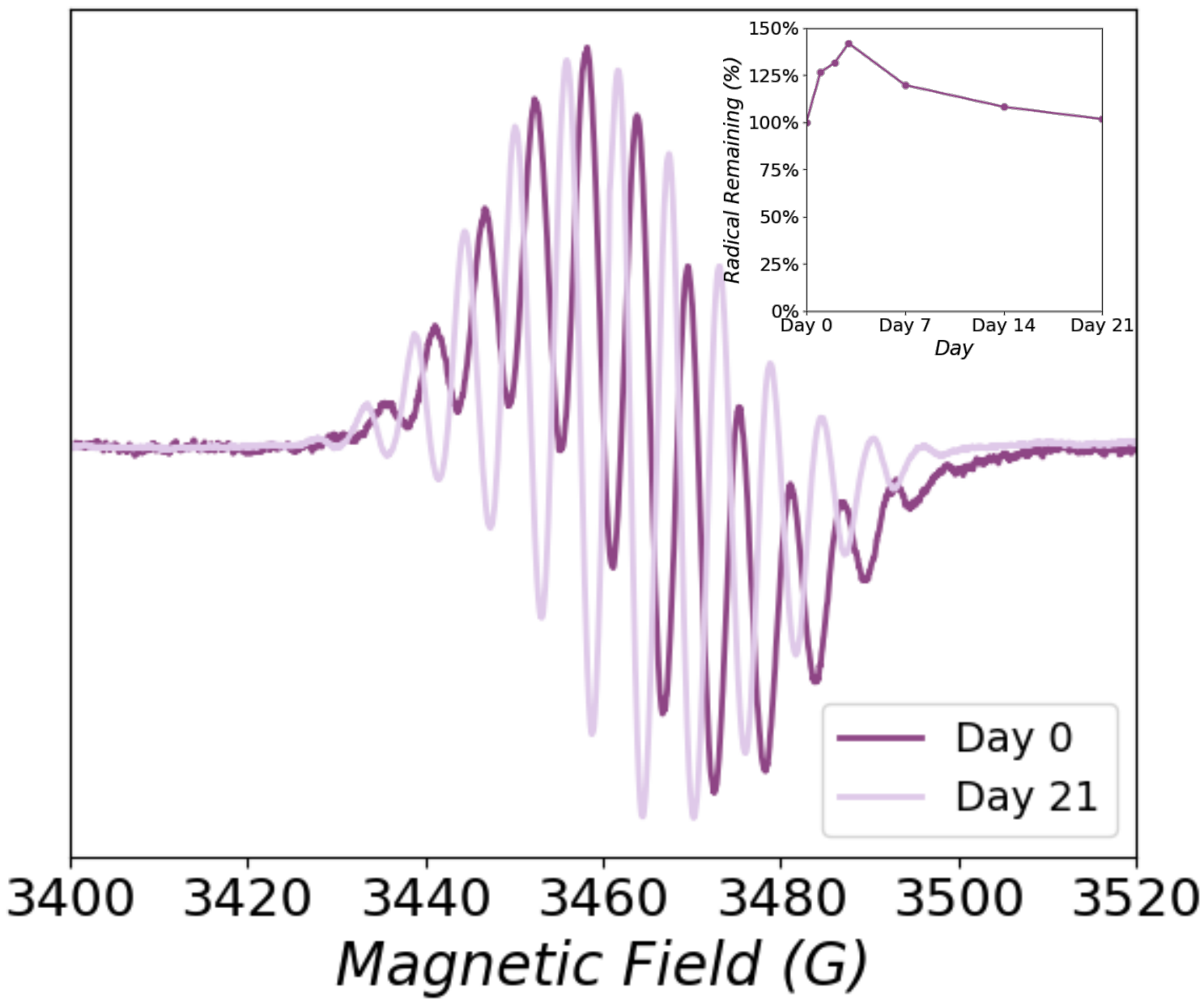

Figure S32. The first derivative X-band (CW) (n = 9.69 GHz) EPR spectrum of **3** in chloroform at 298 K. The spectrum was recorded with a field modulation of 2.1 G and microwave power of 10 mW. Further spectra are collected over 1 month using similar experimental. Radical **3** has been stored in solution, inside a sealed capillary. Inset contains decomposition analysis the peak-to-peak amplitude as a percentage of the original peak-to-peak amplitude of the signal.

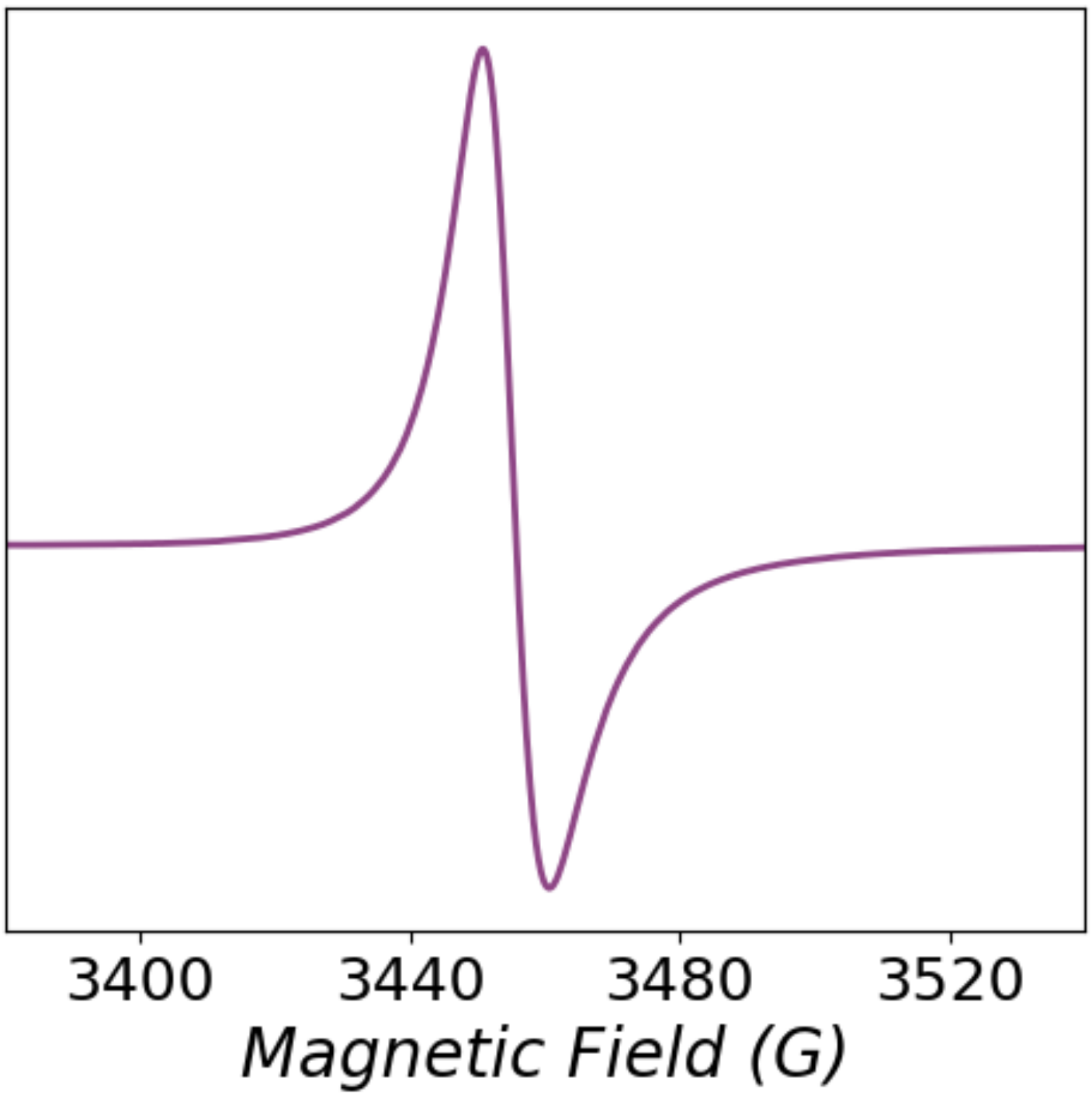

Figure S33. The first derivative X-band (CW) EPR spectrum (*n* = 9.69 GHz) of **3** as a solid at 298 K. The spectrum was recorded with a field modulation of 2.1 G and microwave power of 10 mW.

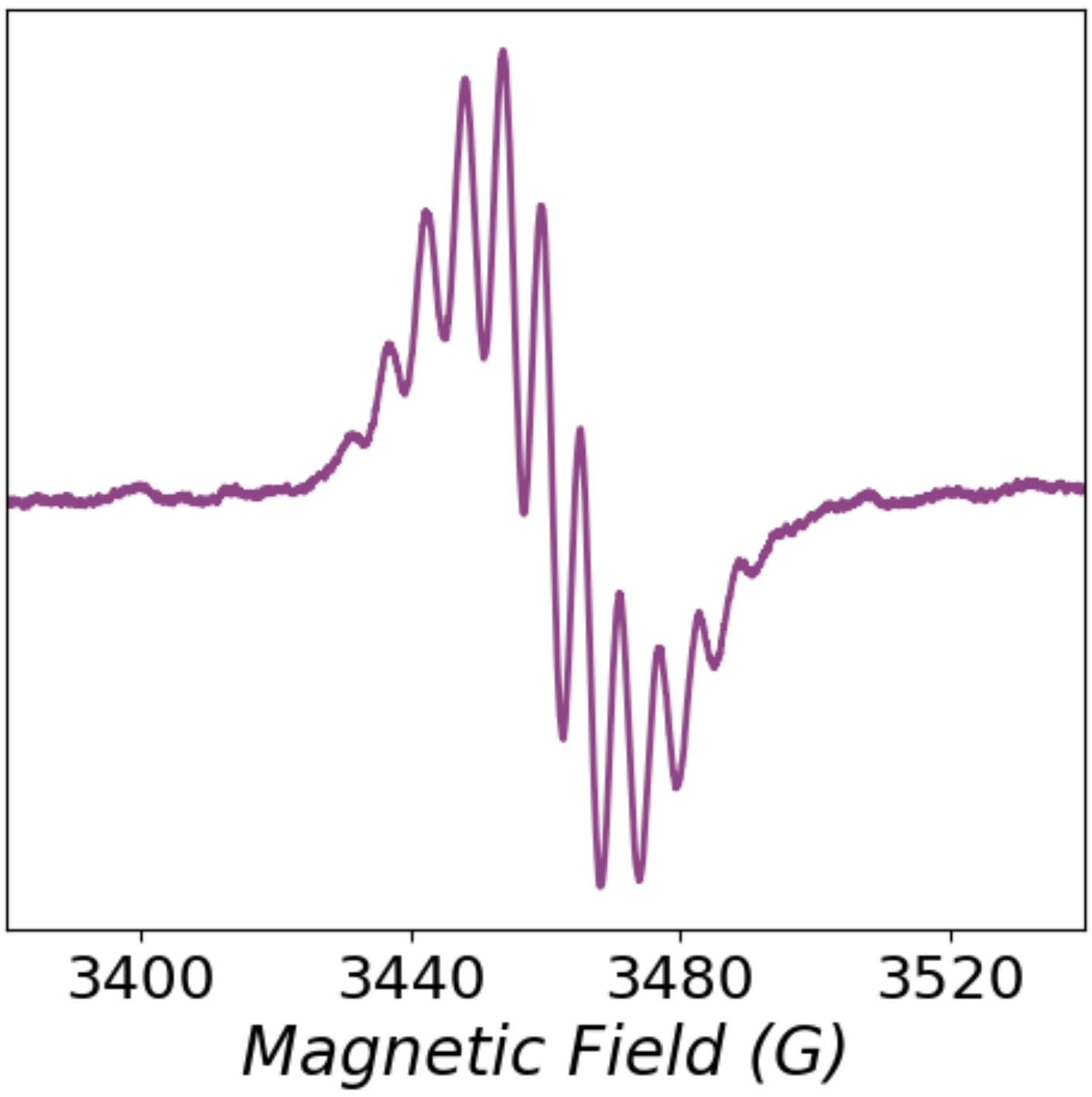

Figure S34. *The first derivative X-band (CW) EPR spectrum (v = 9.69 GHz) of the soluble portion of* ***P2-3*** *in THF within a glass capillary at 298 K. A field modulaton of 2.1 G and power of 40 mW (12 dB attenuation) was used.*

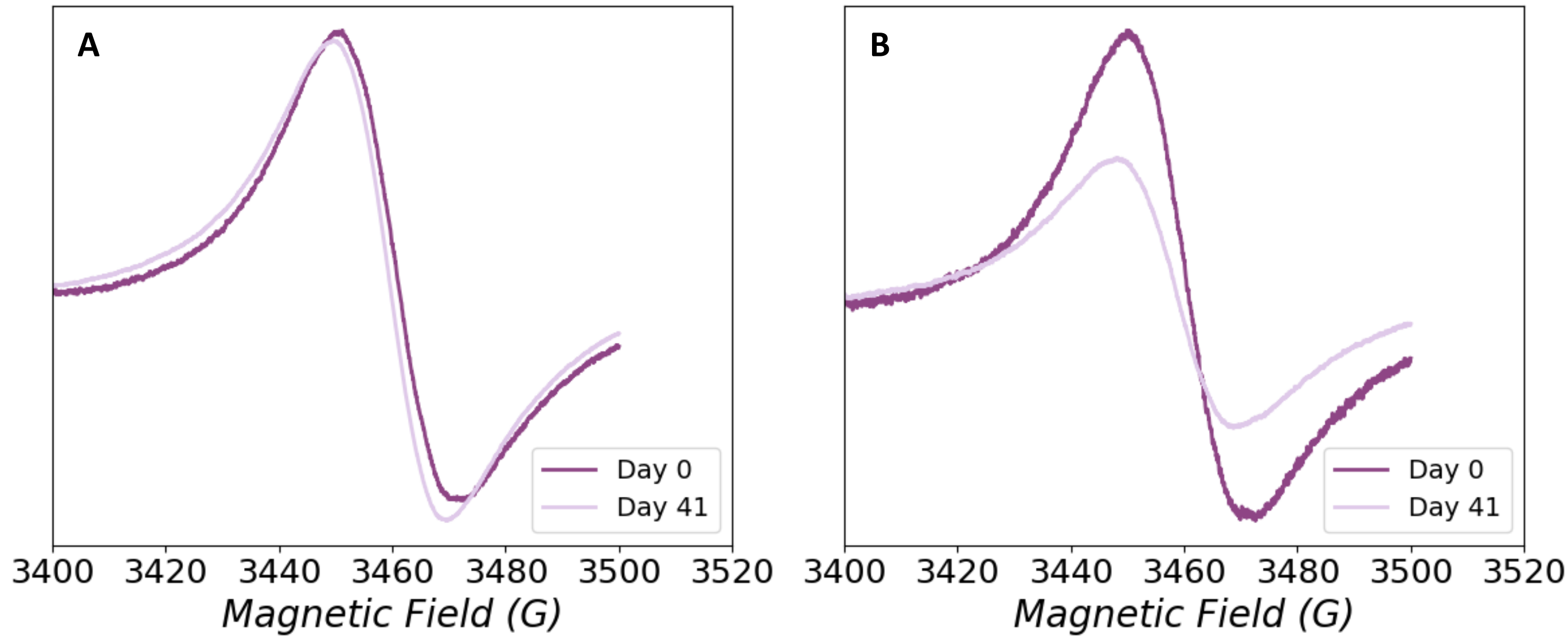

Figure S35. The first derivative X-band (CW) EPR spectrum (*n* = 9.69 GHz) of **P2-2** as a solid at 298 K, in A) a sealed and B) unsealed glass capillary, after 41 days. The spectrum was recorded with a field modulation of 2.1 G and microwave power of 15 mW.

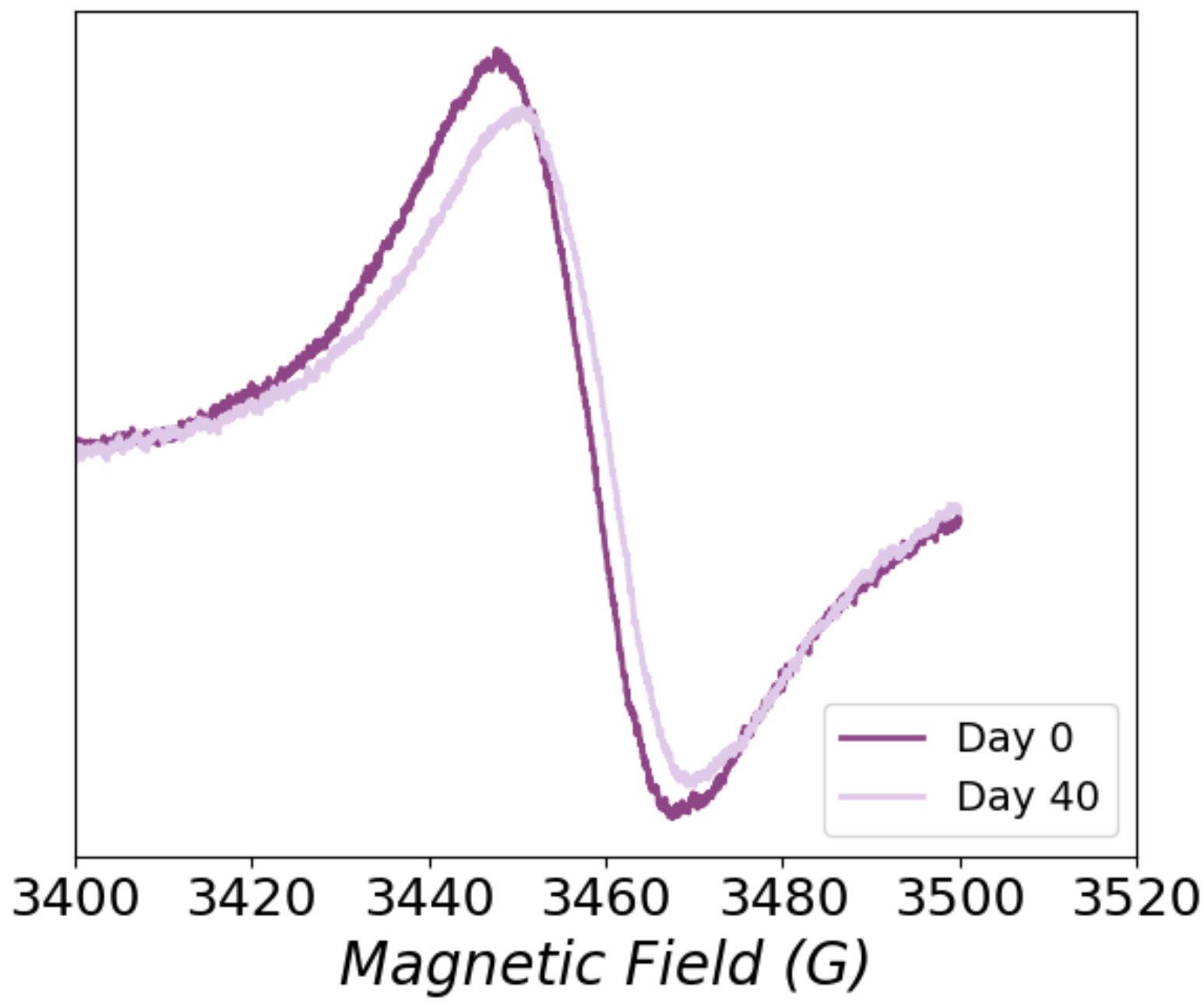

Figure S36. The first derivative X-band (CW) EPR spectrum (*n* = 9.69 GHz) of **P2-2** as a solid at 298 K, in a sealed glass capillary after 40 days. The spectrum was recorded with a field modulation of 2.1 G and microwave power of 15 mW.

IV) Additional Characterisation

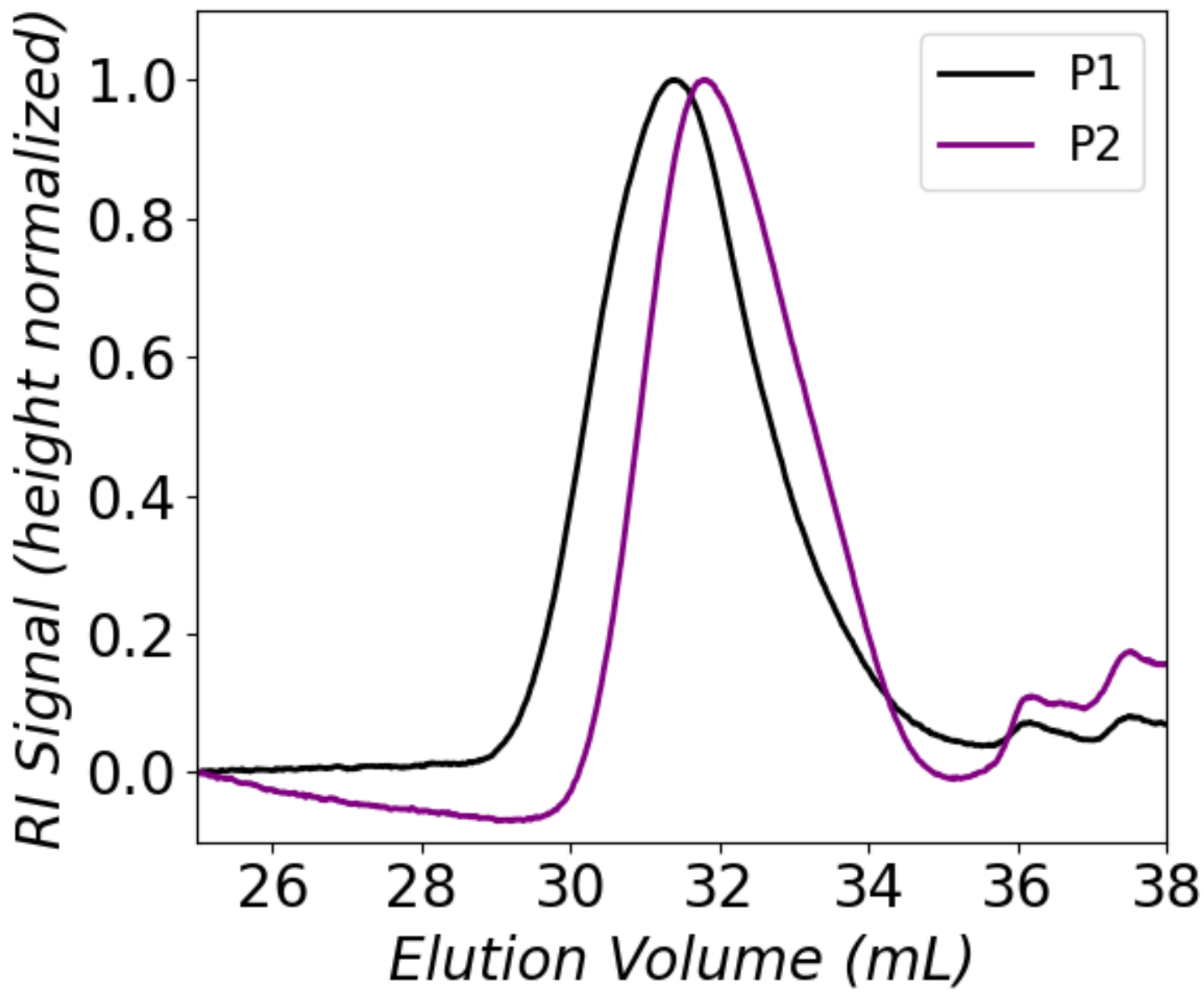

Figure S37. GPC chromatograms of **P1** (black), **P2** (purple).

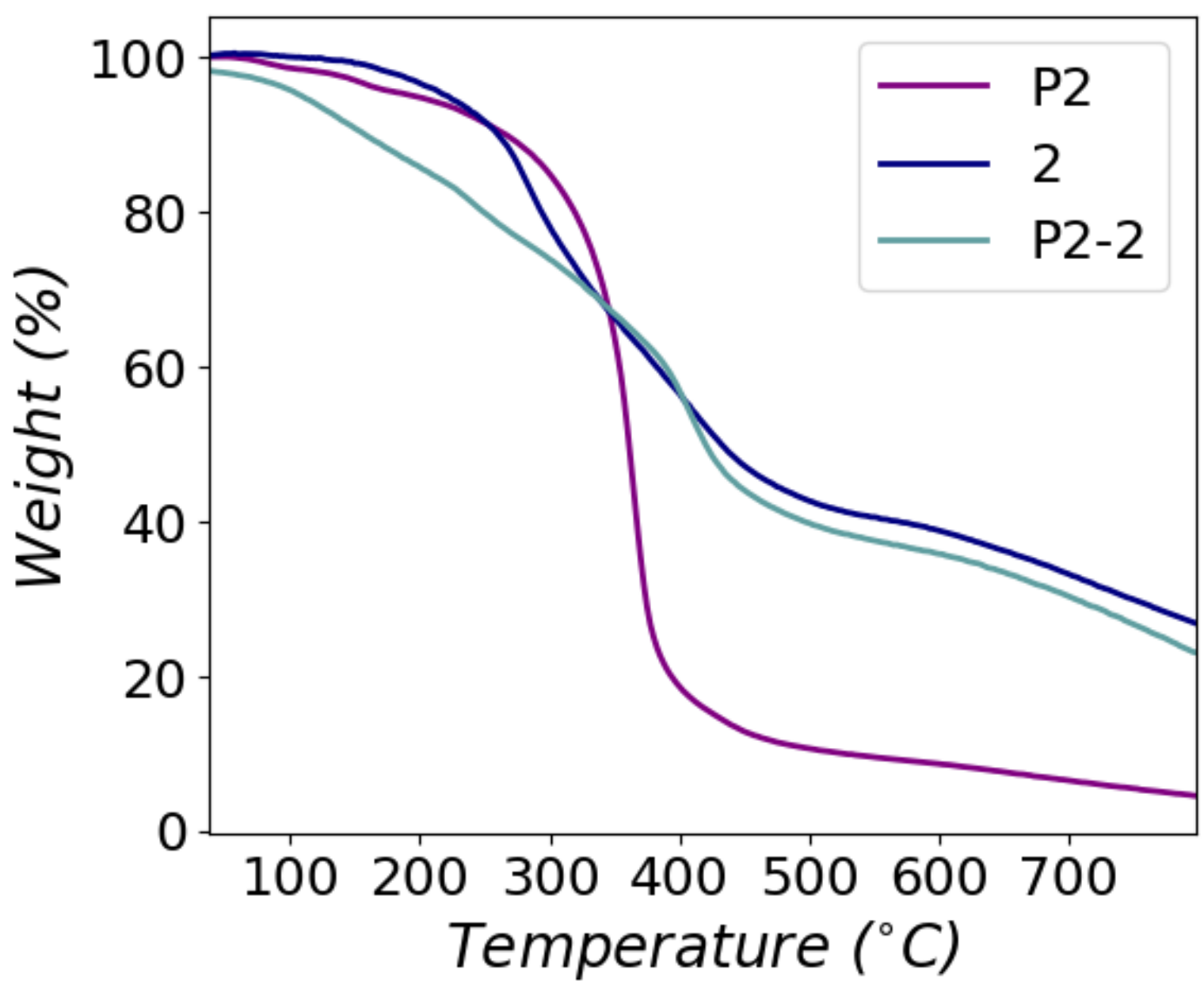

Figure S38. The thermogravimetric analysis curves of weight loss over temperature for **P2**, **2**, and **P2-2**.

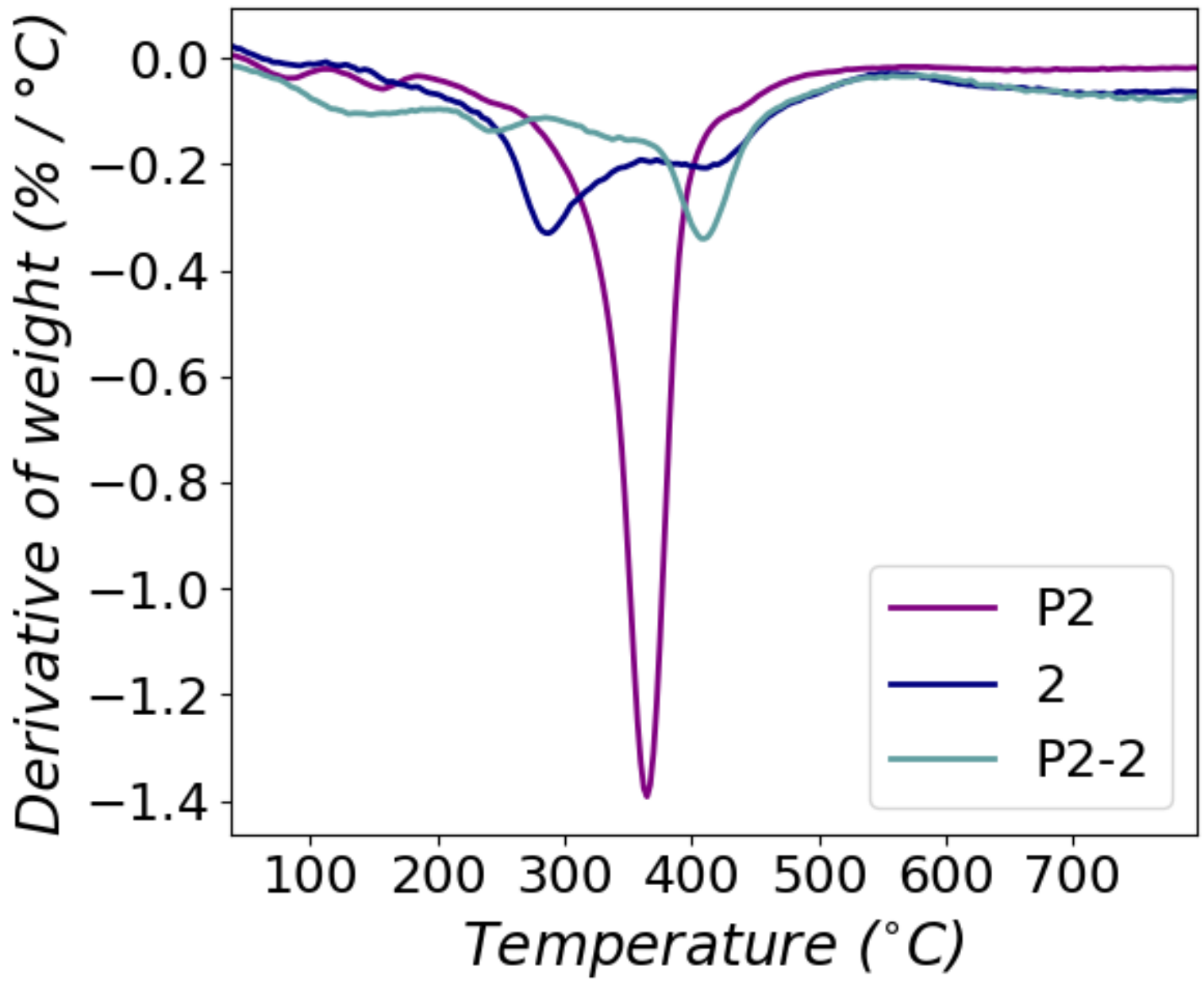

Figure S39. The first derivative of thermogravimetric analysis curves in Figure S38.

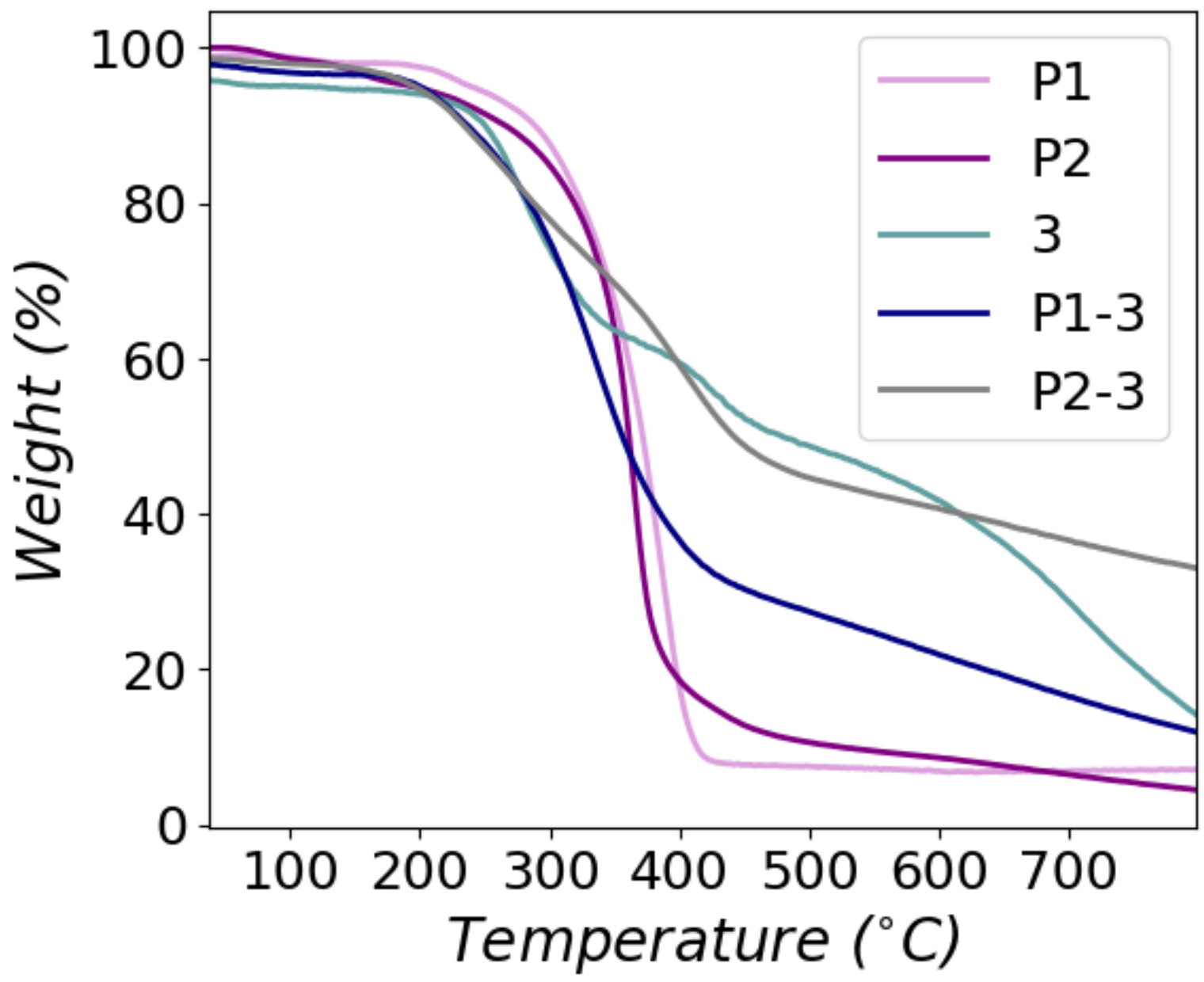

Figure S40. The thermogravimetric analysis curves of weight loss over temperature for **P1**, **P2**, **3**, **P1-3**, and **P2-3**.

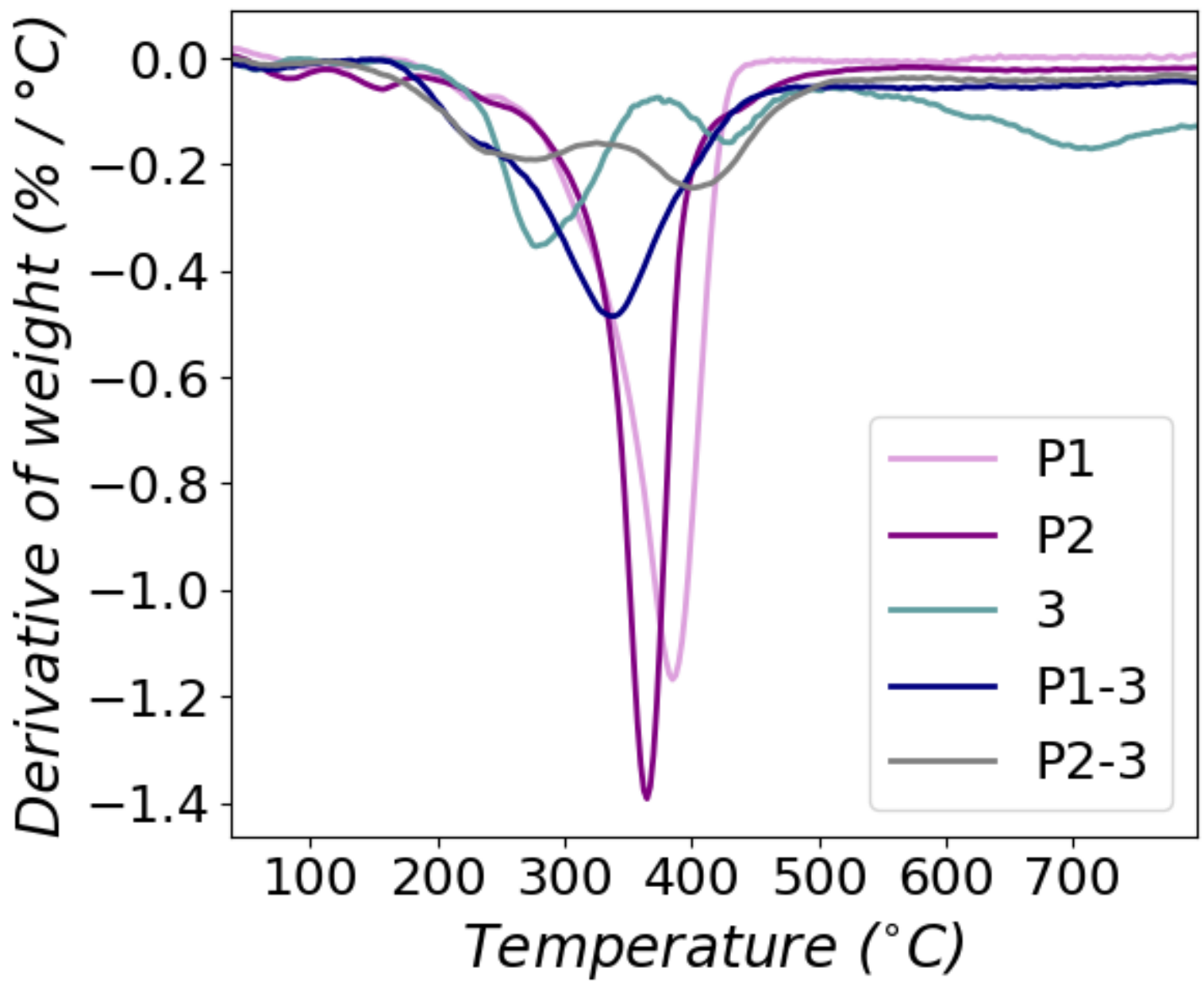

Figure S41. The first derivative of the thermogravimetric analysis curves in Figure S40.

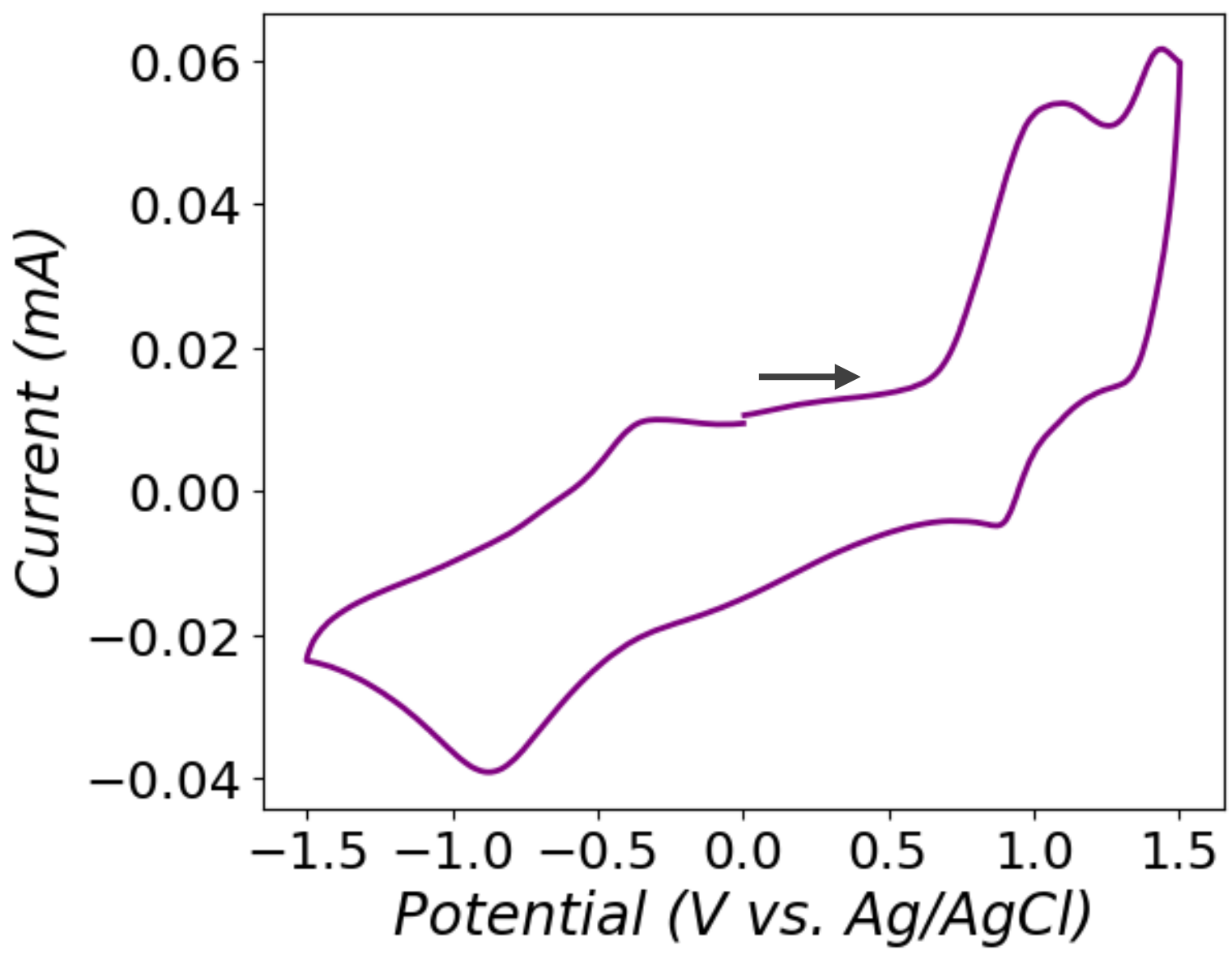

Figure S42. Cyclic voltammogram showing the oxidation and reduction of **1** (10 mM) in 10 mM $nBuNPF_6$ /acetonitrile solution at a scan rate of at 0.5 V/s. Solutions are plotted (IUPAC) vs Ag/AgCl, with a CH Instruments glassy carbon working electrode, Pt wire counter electrode and a leakless Ag/AgCl reference electrode.

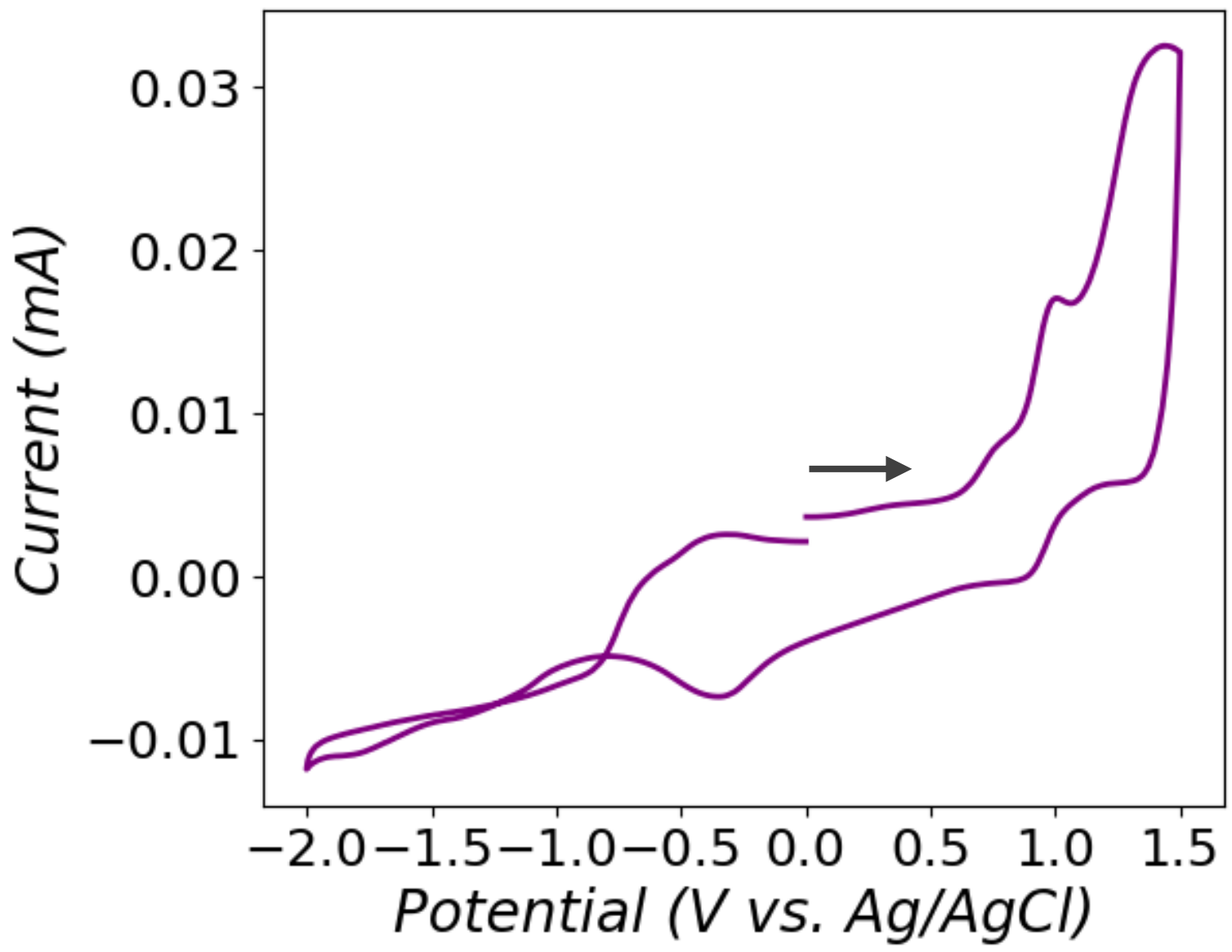

Figure S43. Cyclic voltammogram of **1** (10 mM) after 3 cycles in 10 mM nBuNPF$_6$ /acetonitrile solution at a scan rate of 0.1 V/s. The reduction is irreversible. The oxidation process remains reversible but current has decreased following radical decomposition. Solutions are plotted (IUPAC) vs Ag/AgCl, with a CH Instruments glassy carbon working electrode, Pt wire counter electrode and a leakless Ag/AgCl reference electrode.

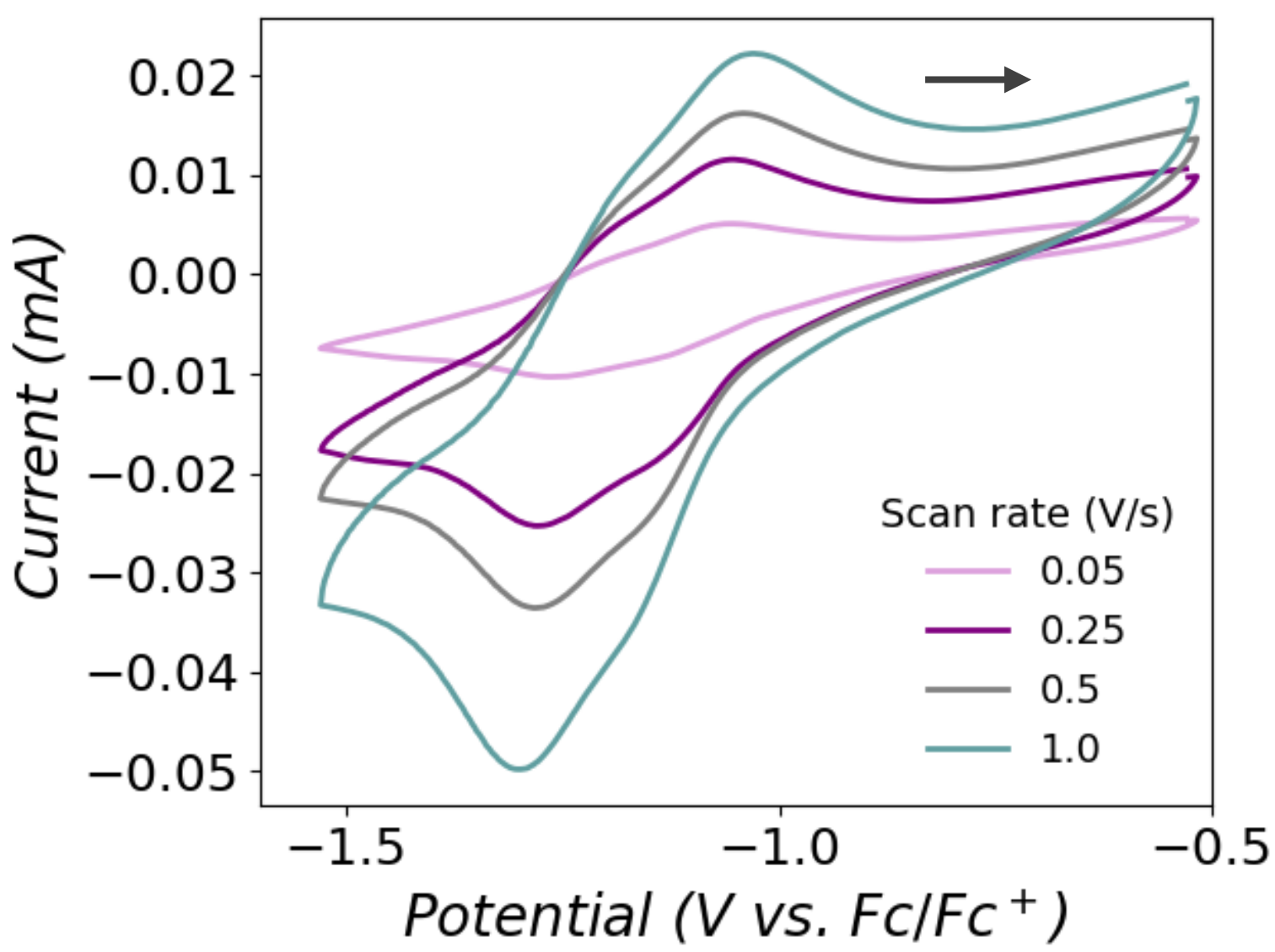

Figure S44. Cyclic voltammogram showing the reduction of **2** (15 mM) in 10 mM nBuNPF$_6$ /DMSO solution at variable scan rate. Solutions are plotted (IUPAC) vs ferrocene/ferrocenium (E1/2 = 0 V), with a CH Instruments glassy carbon working electrode, Pt wire counter electrode and a leakless Ag/AgCl reference electrode.

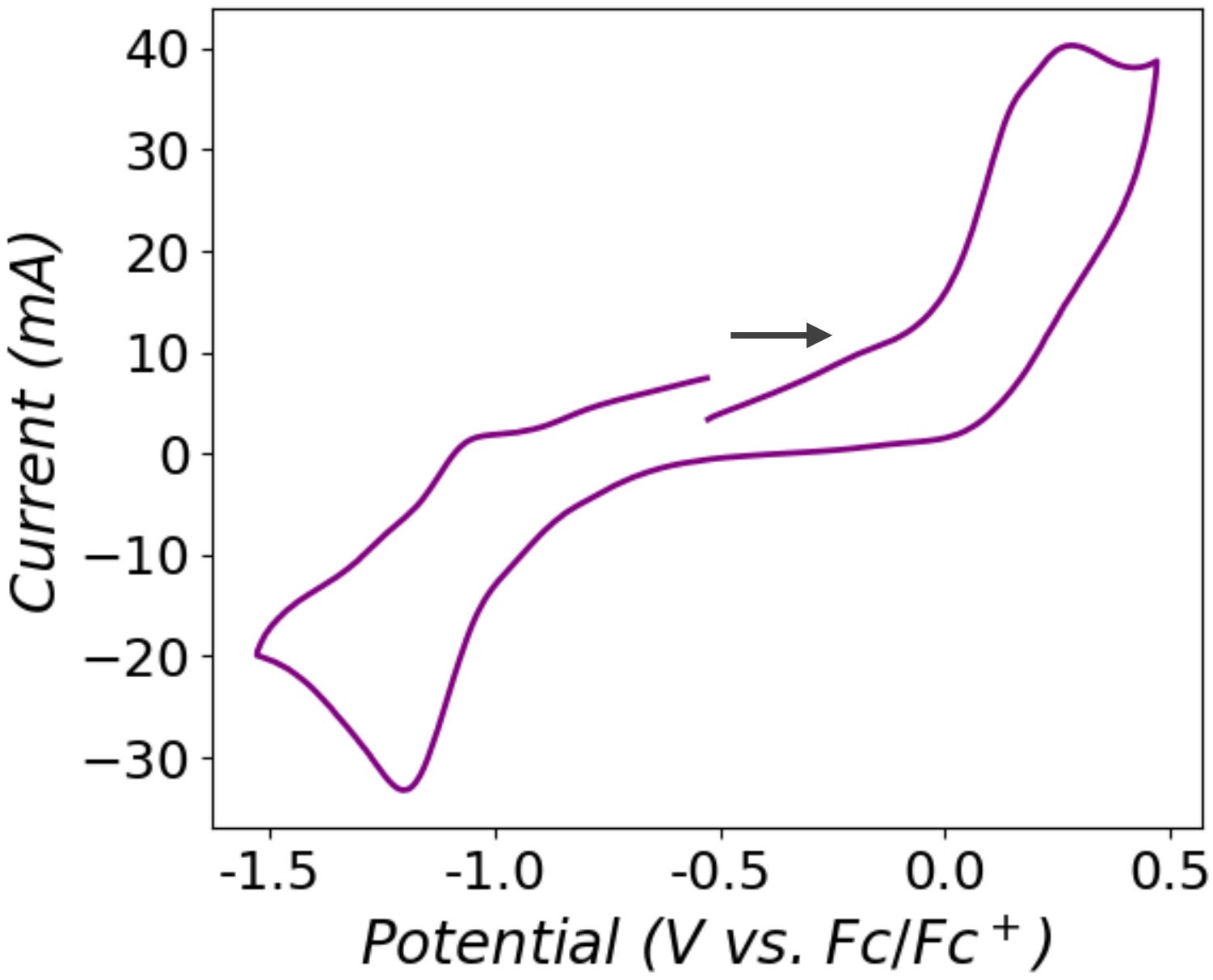

Figure S45. Cyclic voltammogram of **2** (15 mM) in 10 mM nBuNPF_6 /DMSO solution at a scan rate of 0.25 mV/s. Solutions are plotted (IUPAC) vs ferrocene/ferrocenium (E1/2 = 0 V), with a CH Instruments glassy carbon working electrode, Pt wire counter electrode and a leakless Ag/AgCl reference electrode.

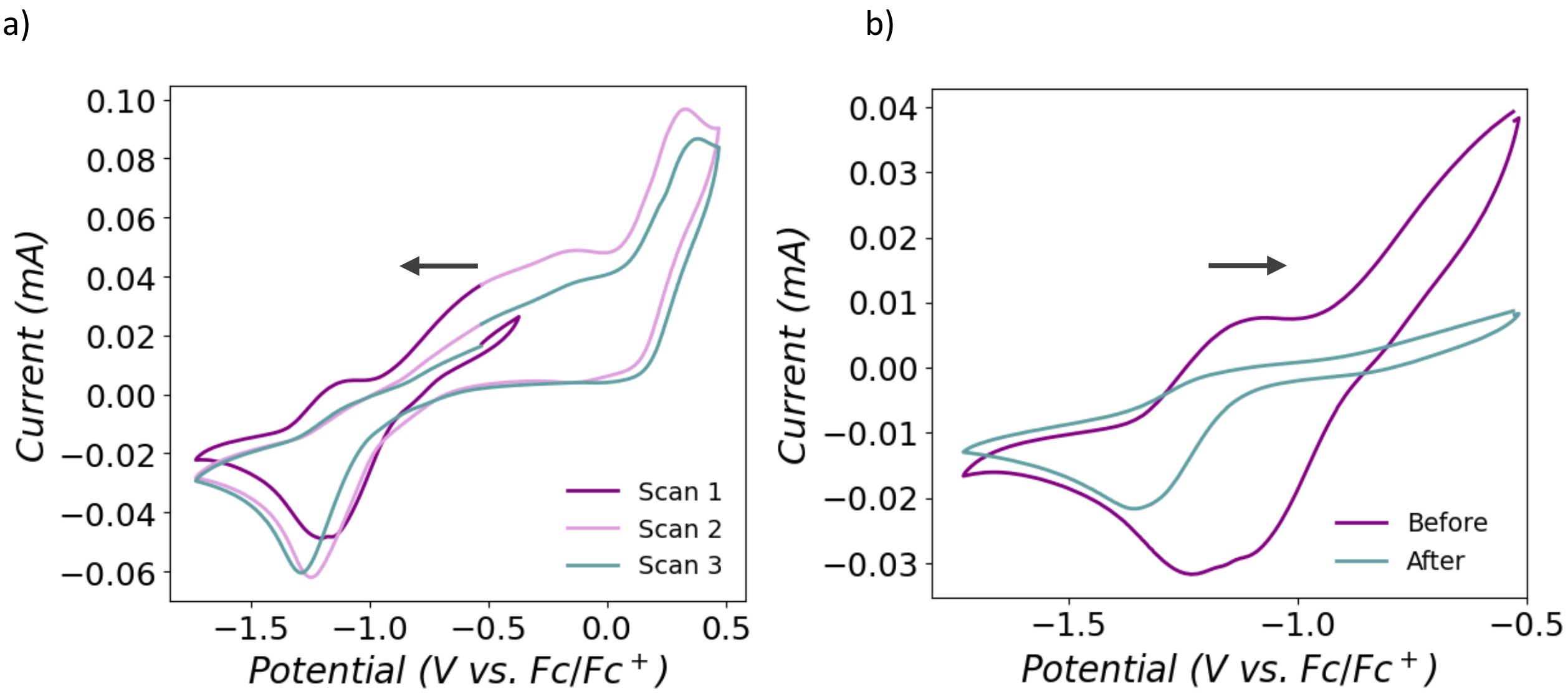

Figure S46. – a) Cyclic voltammogram of **2** (10 mM) in 10 mMnBuNPF_6 /DMSO solution at a scan rate of 0.25 mV/s. The reduction becomes irreversible after the oxidation is scanned. b) Cyclic voltammogram of **3** (10 mM) in10 mM nBuNPF_6 /DMSO solution at a scan rate of 0.25 mV/s. The reduction becomes irreversible after the oxidation is scanned. Solutions are plotted (IUPAC) vs ferrocene/ferrocenium (E1/2 = 0 V), with a CH Instruments glassy carbon working electrode, Pt wire counter electrode and a leakless Ag/AgCl reference electrode.

Hansen Solubility

The solubility parameters of **P2** and **P2-2** (of various degrees of radical functionalisation) were calculated from Hansen solubility parameters, using a group-contribution method.[8] This prediction method is exclusively based on the various bond types and functional groups present within the given molecule. The total solubility parameter, σ_t, is made up of partial/Hansen solubility parameters σ_d, the dispersion solubility parameter, σ_p, the polar solubility parameter, and σ_{hb}, the hydrogen bonding solubility parameter. σ_t is calculated using:

$$\sigma_t = \sqrt{\sigma_d^2 + \sigma_p^2 + \sigma_{hb}^2}$$

σ_t, σ_p and σ_{hb} are calculated through grouping bond types and functional groups, then adding the dispersion, polar and hydrogen bonding contributions respectively. Estimations may vary based on the way in which the functionalities of the repeating unit are grouped. Only first order contributions were considered in the calculations.

$$\sigma_d = (\sum_i N_i C_i + W \sum_j M_j D + 17.3231)$$

$$\sigma_p = (\sum_i N_i C_i + W \sum_j M_j D + 7.3548)$$

$$\sigma_{hb} = (\sum_i N_i C_i + W \sum_j M_j D + 7.9793)$$

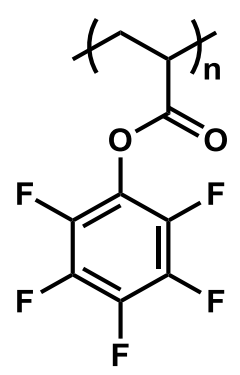

Figure S47. The **P2** repeating unit used for the group contribution Hansen solubility parameter calculation.

Table S2. First-order group contributions to the dispersion, polar partial and hydrogen-bonding partial solubility parameters for the identified functional groups of **P2** units. Using these values, σ_t is calculated to be 23.85 $MPa^{0.5}$

Functional Group	Count	σ_d	σ_p	σ_{hb}
$-CH_2$	1	-0.03	-0.30	-0.41
-CH<	1	0.65	0.65	-0.20
COO	1	0.2039	3.4637	1.1389
AC	1	0.8446	0.6187	0.0084
ACF	5	0.117	0.1856	-0.7182
**Total**		19.57	12.71	4.9

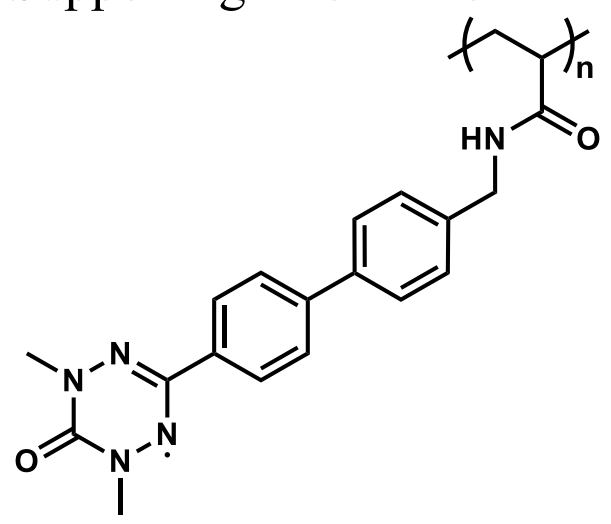

Figure S48. The **P2-2** repeating unit used for the group contribution Hansen solubility parameter calculation.

Table S3. First-order group contributions to the dispersion, polar partial and hydrogen-bonding partial solubility parameters for the identified functional groups of **P2-2** units. Using these values, σ_t is calculated to be 29.57 $MPa^{0.5}$

Functional Group	Count	σ_d	σ_p	σ_{hb}
$-CH_2$	1	-0.03	-0.30	-0.41
-CH<	1	0.65	0.65	-0.20
C=O	2	-0.4343	0.7905	1.8147
CH_2NH	1	0.8116	0.9412	1.34
AC	4	0.8446	0.6187	0.0084
ACH	8	0.1105	-0.5303	-0.4305
-C=N-	1	-0.3074	-0.0012	-5.3956
CH_3N	2	0.8769	1.2046	1.6062
N	1	1.5438	2.578	1.1189
**Total**		25.14	13.44	7.86

For a statistical copolymer, each of the solubility parameters can be calculated based on the composition of the polymer, e.g. where the functionalisation of **P2-2** is *n* (%)**.** Each proceeding calculation can be performed as above using the new solubility parameter values for a statistical copolymer.

$$\sigma_{dt} = \frac{n}{100}\sigma_{d1} + (100 - \frac{n}{100})\sigma_{d2}$$

Hansen solubility data[9] for various solvents can be used to calculate their distance, R_a, from **P2** and **P2-2**. The lower the R_a value for a solvent, the more likely it is to be an effective solvent for the polymer.

$$R_a = \sqrt{4\Delta\sigma_d^2 + \Delta\sigma_p^2 + \Delta\sigma_{hb}^2}$$

Table S4. R_a values for common solvents at various levels of functionalisation of **P2-2**. Calculations are assuming a statistical copolymer made up **P2-2** and **P2** units, varying based on the functionalisation

	Calculated R_a at different levels of P2-2 radical functionalisation				
**Solvent**	**P2 (0%)**	**25%**	**50%**	**75%**	**100%**
DMSO	6.86	7.70	9.39	11.56	13.99
DMAc	8.62	10.23	12.32	14.67	17.19
DMF	7.78	9.13	11.08	13.37	15.85
THF	9.46	11.25	13.43	15.84	18.38
Chloroform	7.20	8.71	10.79	13.17	15.73

VI) Verdazyl ORP electrode characterisation

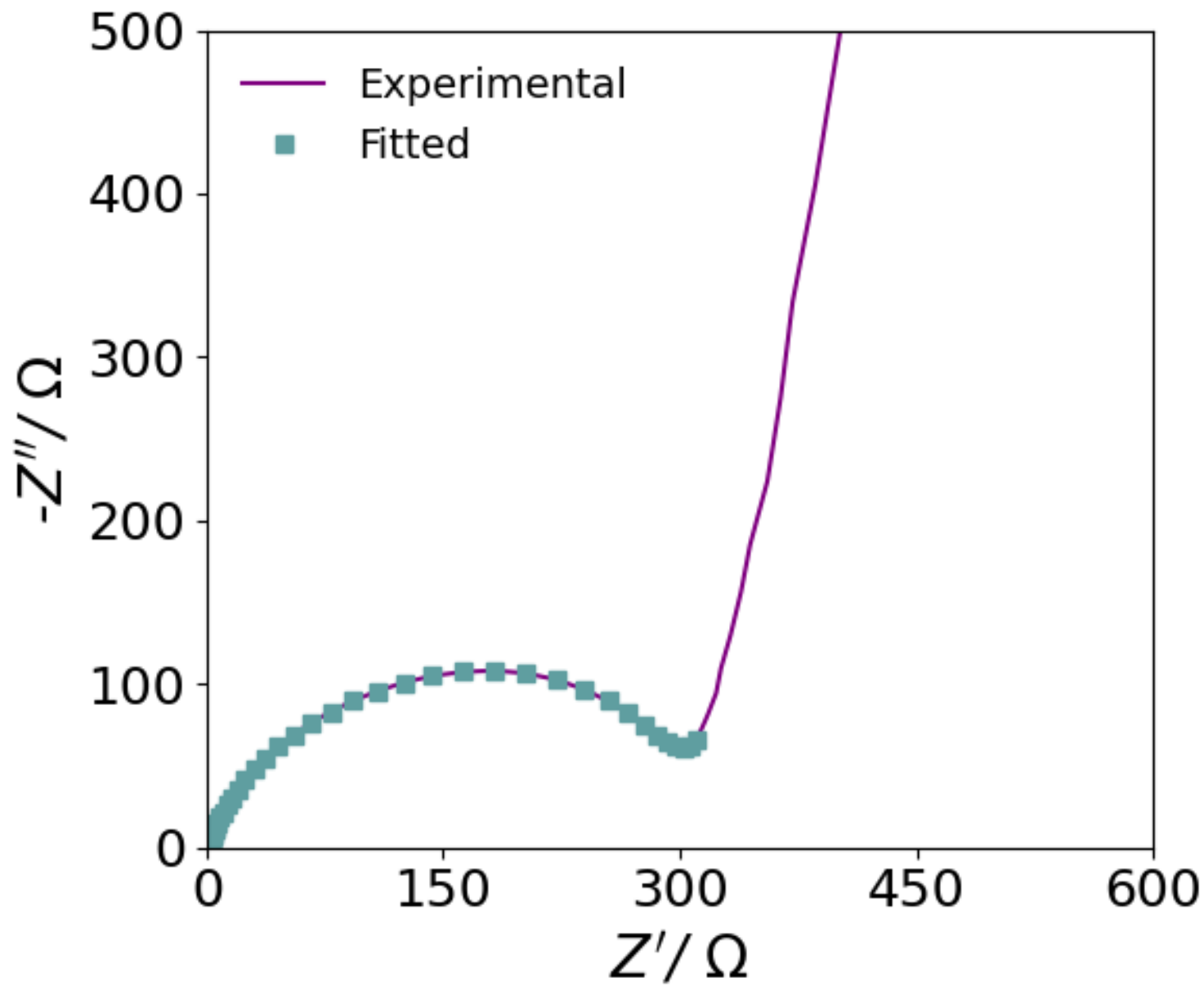

Figure S49. Enlarged view of the mid-frequency region of **P2-2** the Nyquist plot showing the experimental impedance response (purple) and equivalent-circuit fit (blue squares). The close agreement highlights the quality of the fit across the resolved semicircular feature.

Table S5. *Equivalent circuit parameters obtained from fitting the impedance spectrums using the model R1 + (R2||C2) + (R3||C3). R1 represents the series resistance, while R2 and R3 correspond to resolved interfacial processes with associated capacitances C2 and C3*

Polymer	R1	C2	R2	C3	R3
**P2-2**	1.604	7.07E-06	301.3	6.85E-06	26.15

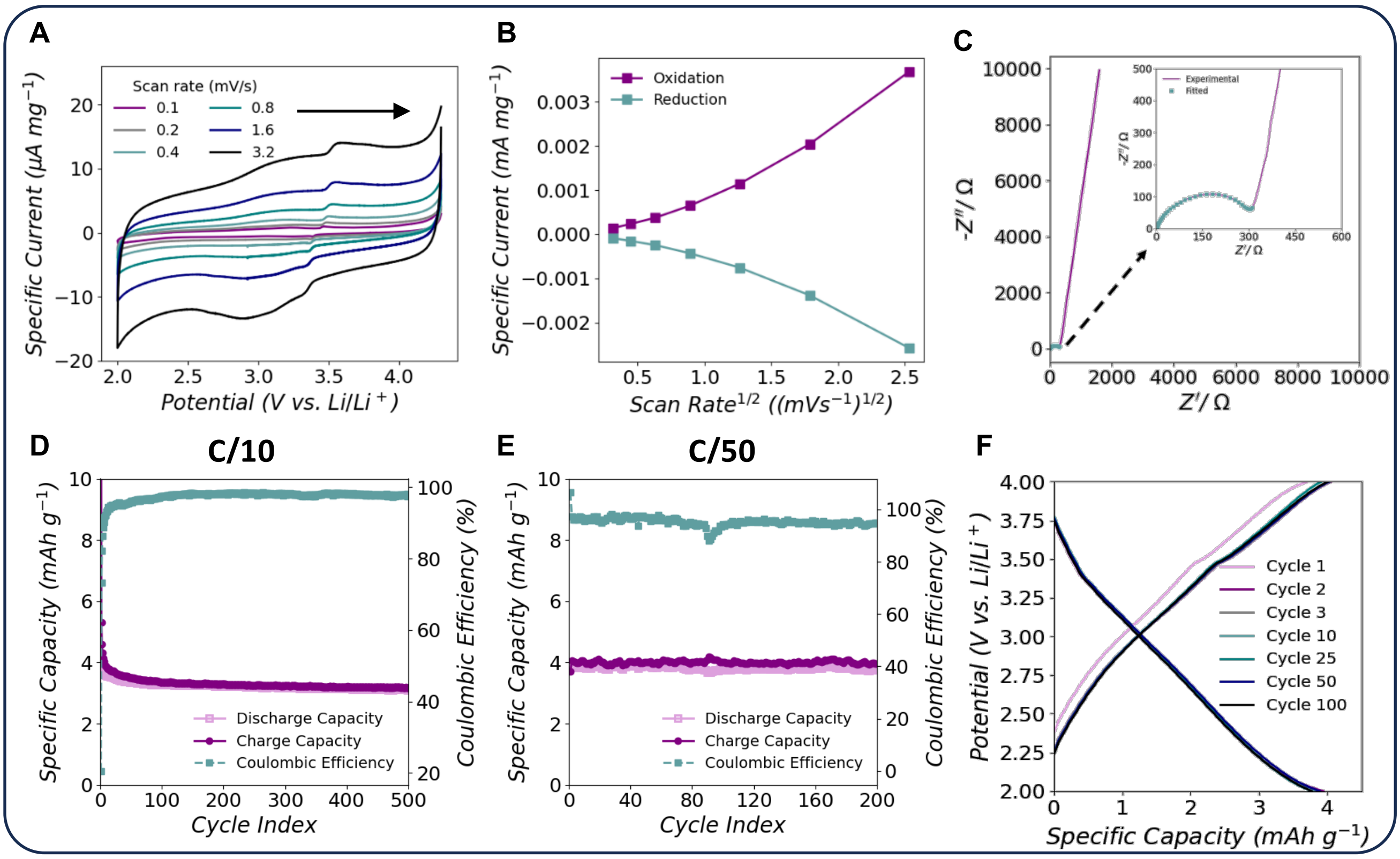

Figure S50. (A) A cyclic voltammogram of the **P2-2** fabricated coin cell at different scan rates. Solutions are plotted (IUPAC) vs Li/Li^+ ($E_{1/2}$ = 0 V). **(B)** The corresponding plot of peak current at different scan rates. **(C)** The Nyquist plot showing the experimental impedance response (purple) and equivalent-circuit fit (blue squares). The close agreement highlights the quality of the fit across the resolved semicircular feature. **(D)** Cycling performance of the **P2-2** coin cell at C/10. **(E)** Cycling performance at C/50. **(F)** Voltage profiles over 100 cycles, showing minimal variation.

References

[1] M. Duggin, W. J. Olivier, A. J. Canty, L. F. Lim, N. Cox, G. F. Turner, S. A. Moggach, S. C. Thickett, A. C. Bissember, R. O. Fuller, *J. Org. Chem.* **2024**, *89*, 9405–9419.

[2] F. A. Neugebauer, H. Fischer, R. Siegel, *Chem. Ber.* **1988**, *121*, 815–822.

[3] G. Rayner, T. Smith, W. Barton, M. Newton, R. J. Deeth, I. Prokes, G. J. Clarkson, D. M. Haddleton, *Polym. Chem.* **2012**, *3*, 2254.

[4] S. Iqbal, M. Saleem, M. K. Azim, M. Taha, U. Salar, K. M. Khan, S. Perveen, M. I. Choudhary, *Bioorg. Chem.* **2017**, *72*, 89–101.

[5] M. R. Golder, H. V.-T. Nguyen, N. J. Oldenhuis, J. Grundler, E. J. Park, J. A. Johnson, *Macromolecules* **2018**, *51*, 9861–9870.

[6] T. A. Ellingsen, S. C. Thickett, R. O. Fuller, *Aust. J. Chem.* **2024**, *77*, CH24085.

[7] S. Stoll, A. Schweiger, *J. Magn. Reson.* **2006**, *178*, 42–55.

[8] E. Stefanis, C. Panayiotou, *Int. J. Thermophys.* **2008**, *29*, 568–585.

[9] C. M. Hansen, *Hansen Solubility Parameters: A User's Handbook* **2007** (CRC Press: Boca Raton).
